# A meta-analysis reveals the protein profile associated with malignant transformation of oral leukoplakia

**DOI:** 10.3389/froh.2023.1088022

**Published:** 2023-02-27

**Authors:** Ana Gabriela Costa Normando, Erison Santana dos Santos, Jamile de Oliveira Sá, Ariane Fidelis Busso-Lopes, Tatiane De Rossi, Fábio Malta de Sá Patroni, Daniela Campos Granato, Eliete Neves Silva Guerra, Alan Roger Santos-Silva, Márcio Ajudarte Lopes, Adriana Franco Paes Leme

**Affiliations:** ^1^ Oral Diagnosis Department, Piracicaba Dental School, University of Campinas, Piracicaba, Brazil; ^2^ Brazilian Biosciences National Laboratory, Brazilian Center for Research in Energy and Materials, Campinas, Brazil; ^3^ Molecular Biology and Genetic Engineering Center, University of Campinas, Campinas, Brazil; ^4^ Laboratory of Oral Histopathology, Health Sciences Faculty, University of Brasilia, Brasilia, Brazil

**Keywords:** oral cancer, oral leukoplakia, malignant transformation, proteins, systematic review, meta-analysis

## Abstract

The search for biomarkers associated with oral leukoplakia malignant transformation is critical for early diagnosis and improved prognosis of oral cancer patients. This systematic review and meta-analysis aimed to assess protein-based markers potentially associated with malignant transformation of oral leukoplakia. Five database and the grey literature were searched. In total, 142 studies were included for qualitative synthesis, where 173 proteins were investigated due to their potential role in malignant progression from oral leukoplakia (OL) to oral squamous cell carcinoma (OSCC). The abundance of these proteins was analyzed in fixed tissues and/or biofluid samples, mainly by immunohistochemistry and ELISA, and 12 were shared by both samples. Enrichment analysis revealed that the differential abundant proteins are mostly involved with regulation of cell death, regulation of cell proliferation, and regulation of apoptotic process. Also, these proteins are mainly expressed in the extracellular region (55.5%), cell surface (24.8%), and vesicles (49.1%). The meta-analysis revealed that the proteins related to tumor progression, PD-L1, Mdm2, and Mucin-4 were significantly associated with greater abundance in OSCC patients, with an Odds Ratio (OR) of 0.12 (95% CI: 0.04–0.40), 0.44 (95% CI: 0.24–0.81), and 0.18 (95% CI: 0.04–0.86), respectively, with a moderate certainty of evidence. The results indicate a set of proteins that have been investigated across OSCC initiation and progression, and whose transcriptional expression is associated with clinical characteristics relevant to the prognosis and aggressiveness. Further verification and validation of this biomarkers set are strongly recommended for future clinical application.

## 1. Introduction

Oral cancer is among the most prevalent cancers worldwide, with over 377,000 new cases occurring in 2020 and more than 177,000 deaths in the same year (1). Despite the large number of cases, it is still a disease frequently diagnosed in advanced stages (2). Late presentation, aggressive local invasion, and metastasis usually result in high relapse rates, contributing to the static 5-year survival rate of 50% around the world (3). Oral squamous cell carcinoma (OSCC) may arise from mucosal disorders collectively grouped as oral potentially malignant disorders (OPMD), which present different rates of malignant transformation.

Oral leukoplakia (OL) is one of the most prevalent and most studied OPMD observed in clinical practice, with an estimated pooled global prevalence of 4.11% (4, 5). OL is defined as a predominantly white plaque of questionable risk having excluded other known lesions (4). The main etiological factors of OL include tobacco and alcohol, although betel quid/areca nut chewing habits also represent an important risk factor especially in South Asian populations (5). Human papillomavirus (HPV) may also play a role in dysplastic leukoplakias (6). Approximately 9.5% of OL lesions evolve into cancer, with an annual transformation rate of 1.56% (7). The significant malignant transformation imposes close follow up measures and appropriate management strategies.

Predicting the risk of malignant transformation remains a significant clinical challenge and it is of ultimate importance since early diagnosis may prevent oral cancer rise (8). Currently, early diagnosis and risk screening may be accomplished by an oral biopsy followed by histopathological analysis. Epithelial dysplasia is the main indicator of malignant transformation risk of OL and increasing grades of dysplasia indicates higher risk of progression to carcinoma (9). However, the histological grading of oral epithelial dysplasia is considered subjective, with both intra and inter-examiner variations, raising the concern of reproducibility (9). Also, the decision of following or surgically treating an OPMD is challenging and, even after surgical excision, recurrence or malignant transformation may occur (10). Thus, more precise methods for assessment of malignant potential of a given disorder are imperative and could assist in the early diagnosis of OSCC (11).

Molecular biomarkers approach emerges as a useful way of identifying and characterizing alterations indicative of malignant transformation that would be valuable for clinicians to precociously intervene and prevent OSCC emergence. Proteins are the biomolecules that directly execute most biological processes, suggesting they are valuable predictors of disease progression (12, 13). Different studies have suggested the potential of proteomics to reveal prognostic biomarkers of OSCC in tissue and biofluids such as serum and saliva (14–16). Therefore, the search for proteins is not only necessary but also may reveal new targets to objectively and precisely determine the OPMD risk of malignant transformation.

In this context, this systematic review aimed to determine the protein profile potentially associated with malignant transformation of OL, and that could indicate susceptibility to disease progression. Previous reviews have already assessed general prognostic biomarkers in OPMDs, in oral dysplasia, and in tissues of OL (8, 10, 17). However, no systematic reviews have assessed with a meta-analytical approach the protein expression associated with malignant transformation risk in oral leukoplakia lesions that could assist in future studies.

## 2. Methods

### 2.1. Eligibility criteria

The focused research question and the inclusion criteria were based on the acronym PECOS (Population, Exposure, Comparison, Outcomes, Studies) of which: (P) patients diagnosed with oral leukoplakia; (E) presence of proteins that indicate or suggest malignant transformation; (C) patients with oral cancer; (O) association of protein expression and malignant transformation of OL into oral cancer; (S) observational studies (cross-sectional, cohort, or case-control) and clinical trials.

Studies presenting any of the following criteria were excluded: (1) studies assessing biomarkers after any intervention; (2) studies with no comparison group; (3) studies with no individualized data for oral leukoplakia; (4) studies which do not specify the potentially malignant disorder; (5) studies assessing virus as biomarkers (HPV, HIV, EBV, HSV); (6) studies assessing hairy leukoplakia; (7) studies evaluating non-oral leukoplakia; (8) studies that do not assess possible progression/transformation; (9) reviews, letters to the editor, conference abstracts, personal opinions, book chapter, *in vitro* or *in vivo* animal studies, and case reports; (10) full text copy not available; (11) language restriction [studies not published in Latin (Roman) alphabet]; (12) studies containing data already reported in other studies; (13) No protein profile assessment.

### 2.2. Information sources and search strategies

Individualized search strategies were performed for each of the following databases: EMBASE, LILACS, PubMed, Scopus, and Web of Science (Supplementary Appendix S1). A further search of the grey literature was carried out on Google Scholar and ProQuest. In addition, the references of studies included in the systematic review were manually reviewed for potential additional papers. Searches in all databases were carried out on September 4th, 2021. The studies retrieved from different databases were imported into a reference manager software (Endnote Web, Clarivate Analytics, Philadelphia, PA), in which duplicated references were automatically removed. No limits in terms of publication date were applied to the search strategy.

### 2.3. Selection process

The study selection was performed in two phases. In the first phase, two reviewers (AGCN and ESS) independently read the titles and abstracts of retrieved studies and applied the eligibility criteria. The first phase was performed on Rayyan® software (18). The remaining duplicates were manually removed, and afterwards all references were read. Those studies that seemed to accomplish all inclusion criteria were moved forward to the second phase where full texts were read, and the selection criteria were applied to confirm whether the studies fulfilled all eligibility criteria. The same two reviewers were independently involved in phase 2. References of all included studies were assessed for possible missing studies that could be included. Disagreements in either phase were resolved by discussion and mutual agreement between the reviewers.

### 2.4. Data collection process and data items

The most relevant data from included studies were collected by one reviewer (AGCN) and crosschecked by a second reviewer (ESS). Any disagreements were also resolved by discussion and mutual agreement among the authors. The experts were involved if required for a final decision. Extracted data included: first author, year and country of publication, study design, sample size, gender, specimens and method of assessment, protein names, main results, and conclusions. If relevant data were not reported or incomplete, attempts were made to contact the corresponding authors by e-mail to retrieve the missing information.

### 2.5. Risk of bias assessment

The risk of bias of individual studies was independently assessed by two calibrated reviewers (AGCN and ESS) using the Joanna Briggs Institute (JBI) Critical Appraisal Tools for Cohort and for Analytical Cross-sectional studies (19). Briefly, methodological quality of cohort and cross-sectional studies was assessed by 11 and 8 questions, respectively, to address the possibility of bias in its design, conduct, and analysis. Risk of bias score was calculated dividing the frequency of “yes” answers above the total number of questions. Studies were characterized as having a high risk of bias when it reached up to 49% score “yes”, as moderate when the study reached 50% to 69% score “yes”; and as a low risk of bias when the study reached more than 70% score “yes”.

### 2.6. Effect measures

The main outcome was the association between the abundance of a given protein biomarker and the potential of malignant transformation of OL. Malignancy potential was considered when there was a difference in protein expression between two or more groups (*p* < 0.05). Secondary outcomes were given by the scores of relative abundance among the groups, IHC staining intensity values, or concentration of a given protein and their differences among the comparison groups (OSCC vs. OL). The effect measure considered was the odds ratio (OR) which was calculated based on the total number of included patients in each group (OSCC and OL) and the number of patients expressing positivity for a particular protein.

### 2.7. Synthesis methods

A qualitative analysis was performed grouping the proteins by type of sample, tissue or biofluids, and by comparing data reported in included studies regarding primary and secondary outcomes. The association meta-analyses were performed following the appropriate Cochrane Guidelines (20). Review Manager 5.4 software (RevMan 5.4, The Nordic Cochrane Centre, Copenhagen, Denmark) was used to perform the association meta-analysis, with the OR and 95% confidence intervals (CI) determined at a significance level of 5%. Statistical heterogeneity was calculated using an inconsistency index (*I*^2^), and a random effect model was used among all analysis.

### 2.8. Certainty assessment

The certainty of the cumulative evidence was assessed by the Grading of Recommendation, Assessment, Development, and Evaluation (GRADE) instrument (21). The assessment was applied to the different outcomes evaluated in the present review and it was based on study design, risk of bias, inconsistency, indirectness, imprecision, and other considerations. The certainty of evidence was scored as high, moderate, low, or very low. A GRADE evidence profile was performed using the online software GRADEpro (22).

### 2.9. Functional enrichment analysis of assessed proteins

Functional enrichment analysis for Gene Ontology (GO) terms (biological processes, molecular functions, and cellular components) was conducted through the PANTHER Classification System (http://pantherdb.org/) considering the GO Ontology database (http://geneontology.org/; DOI: 10.5281/zenodo.5228828 Released 2021-08-18), and binomial test with Bonferroni correction for multiple testing. The retrieved data were further plotted on GraphPad Prism version 9.2.0 (GraphPad; https://www.graphpad.com).

### 2.10. Association of protein-based markers with OSCC clinical characteristics using public database

Association of gene expression levels of all assessed proteins with OSCC clinical characteristics was performed using The Cancer Genome Atlas (TCGA). Firstly, transcript levels were retrieved from the public repository TCGA available in the Genomic Data Commons Data Portal (GDC) (https://portal.gdc.cancer.gov) for OSCC. The association with clinical and pathological features was performed using gene expression information from primary tumors and clinical data retrieved from OSCC patients in TCGA repository, totalizing 398 individuals included from the following oral areas: (i) alveolar ridge, (ii) base of tongue, (iii) buccal mucosa, (iv) floor of mouth, (v) hard palate, (vi) oral cavity, (v) oral tongue, (vi) oropharynx, and (vii) tonsil. The selected targets were evaluated according to different clinical categories, as follows: (i) recurrence, (ii) death status, (iii) lymph and vascular invasion, (iv) margin status, (v) tumor histological grade, (vi) lymph node status, (vii) pathologic stage, (viii) tumor size, (ix) perineural invasion, (x) extracapsular nodal spread, (xi) alcohol and tobacco history, (xii) HPV status, and (xiii) primary therapy outcome. For unbiased group assignment, we used mclust package (23) under R environment. Data were tested for normality and homogeneity of variance using Shapiro-Wilk test (*p*-value ≤ 0.05) to drive decisions of parametric or non-parametric tests for group comparison with the clinical categories (24).

### 2.11. Analysis of driver genes

HNSCC-driver mutated genes were retrieved from the Integrative OncoGenomics (IntOGen) pipeline (25). The author compiled significant mutations reported for 4 cohorts of HNSCC: TCGA (502 HNSCC primary tumors), cBioPortal (70 HNSCC primary tumors), Hartwig Medical Foundation (63 HNSCC metastasis), and Pan-Cancer Analysis of Whole Genomes (PCAWG) (56 HNSCC primary tumors). Sixty-two driver mutations were reported for HNSCC and manually compared with our list of identified proteins in the included studies of the present systematic review.

## 3. Results

### 3.1. Study selection

**Figure 1:**
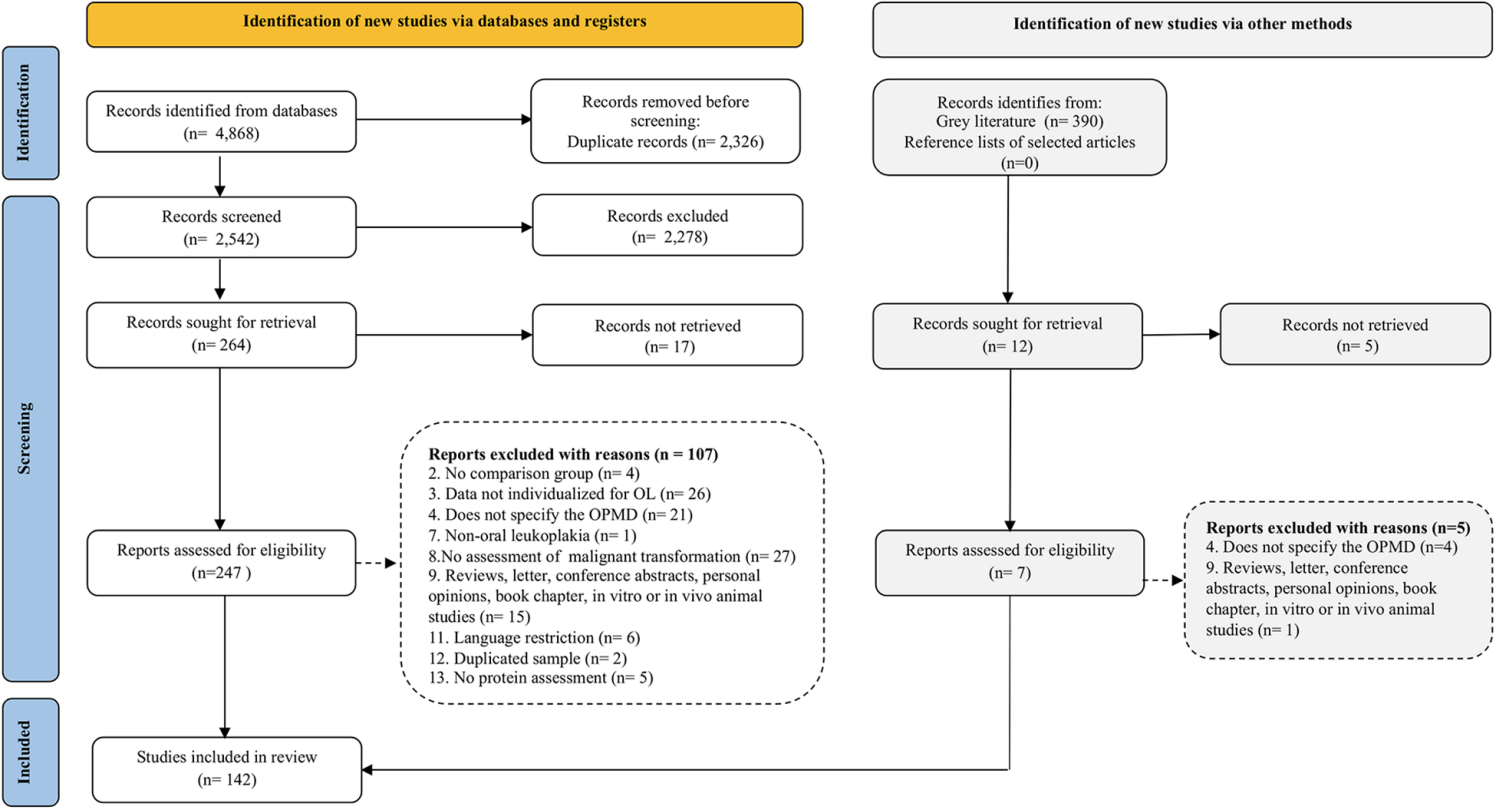
Flow diagram of literature search and selection criteria adapted from PRISMA 2020 (26).

By performing database searches, 4,868 records were identified. After removing the duplicates, 2,542 references remained and had their titles and abstracts screened. In total, 264 references met the eligibility criteria and were retrieved for detailed evaluation. Also, 12 additional studies retrieved from grey literature were included, totalizing 276 articles that were considered for full-text assessment. The eligibility criteria were confirmed, and 112 articles were excluded, in addition to other 22 studies that could not be retrieved as full-text copy was not available (Supplementary Appendix S2). Lastly, 142 studies were included for qualitative synthesis and had their data extracted. The study selection process is described in the flow diagram (Figure 1) and the reference list of the included studies is presented in Supplementary Appendix S3.

### 3.2. Study characteristics

Most of the included papers were cross-sectional studies (*n* = 130; 91.5%) while the remaining were retrospective cohorts (*n* = 12; 8.5%). Most studies were conducted in Asia, especially India and China (42.3% and 19.4%, respectively), followed by Europe. All the studies were published in the English language between 1993 and 2021, with the majority having been published in the last decade (*n* = 106; 75%). The total sample comprised 15,248 patients, of which 6,984 patients with OSCC, 5,767 OL patients, and 2,497 healthy controls. Of the total studies, 135 studies confirmed the OL diagnosis by histopathological analysis, two performed clinical diagnosis according to predetermined diagnostic and staging criteria (27), and five did not clearly report how the diagnosis was performed.

Among OSCC patients, 43.4% were male and 20.4% were female, whilst the remaining patients had no gender reported by these studies. Similarly, most OL patients were male (35.2%) and 18.7% were represented by female patients, although most OL patients have not had their gender data reported. Some studies (28/142) assessed the expression of specific proteins in OL compared only to OSCC, whereas most of the included studies (116/142) compared OL, OSCC, and healthy individuals, which allows for a more accurate assessment of protein abundance according to the progression of the disease.

Among the included studies, there were 173 distinct assessed proteins which were analyzed from both formalin-fixed paraffin embedded (FFPE) tissues and biofluid samples (saliva and serum). The most frequently used technique to assess protein expression was immunohistochemistry (84.7%), followed by ELISA (11.1%), Western Blotting (2.8%), and Mass Spectrometry (2.1%). Supplementary Appendix S4 presents the qualitative synthesis of the expression profile of all proteins in the process of malignancy from control normal epithelium (C) evolving to OL without dysplasia, to dysplastic OL, culminating in OSCC. Some proteins showed progressive overexpression according to disease progression (C < OL < OSCC), other proteins were downregulated according to the course of the disease (C > OL > OSCC), and other proteins had contrasting results between studies. The detailed qualitative synthesis of the included studies assessing proteins in FFPE tissues is presented in Supplementary Appendix S5 and of the studies assessing proteins in biofluids is shown in Supplementary Appendix S6.

### 3.3. Risk of bias in the studies

Among the cross-sectional studies, 27 studies were assessed as low risk of bias, 57 presented moderate risk of bias, whereas 46 studies were scored as having a high risk of bias. Most studies described the sample subjects in detail, measured the proteins in a valid and reliable way, and used objective, standard criteria for measurement of the conditions. On the other hand, most of cross-sectional studies failed in clearly defining the sample inclusion criteria, did not identify confounding factors nor stated strategies to deal with them, and did not measure the OL and OSCC conditions in a reliable way.

**Figure 2:**
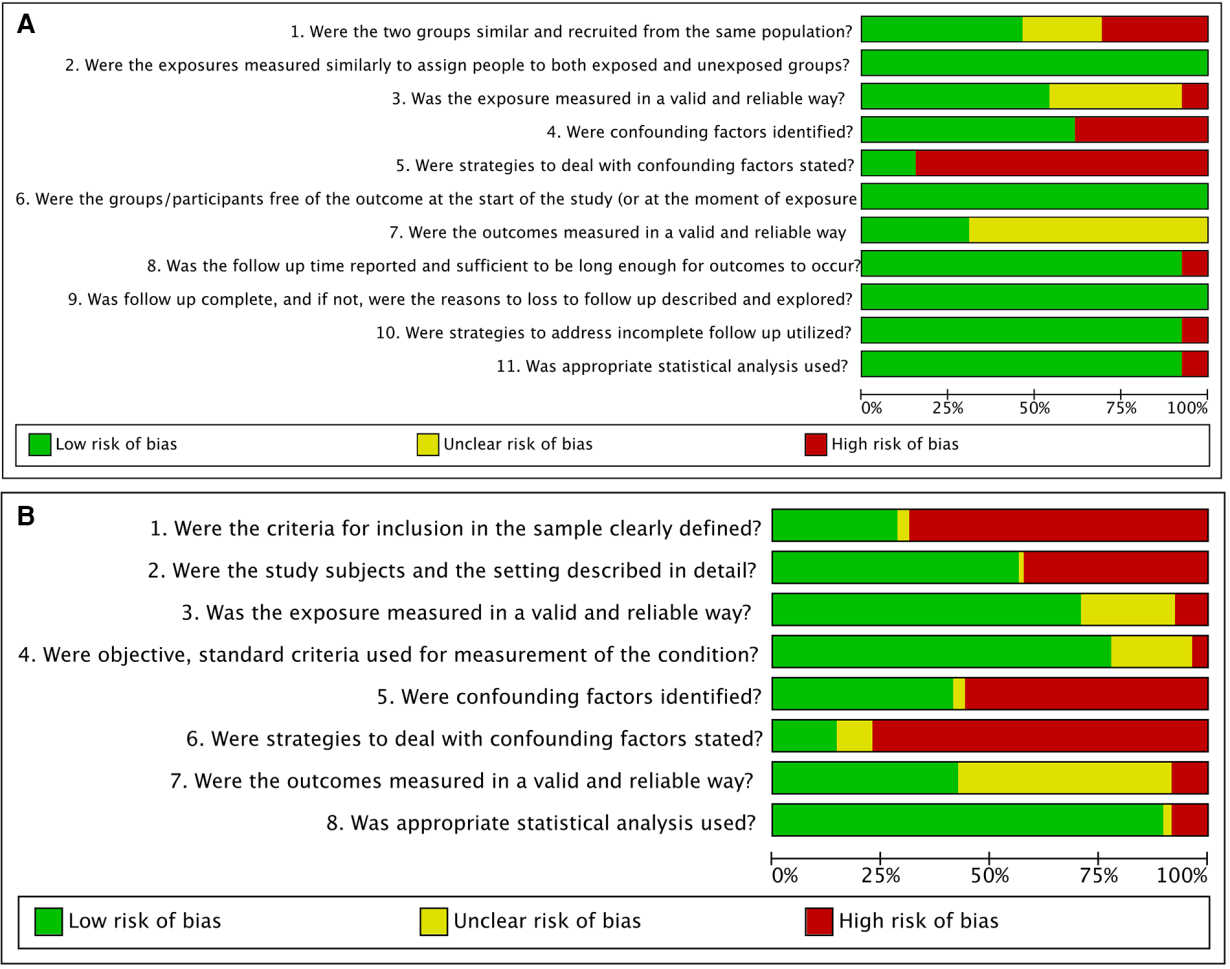
Risk of bias summary: review authors’ judgements about each risk of bias item presented as percentages across cohorts (**A**) and cross-sectional studies (**B**).

Regarding retrospective cohorts, four of them were rated as moderate risk of bias and the other 8 were graded as low risk of bias. Most studies successfully measured the conditions to assign people for the groups (Control, OL, and OSCC), assured that the participants had not undergone malignant transformation at the start of the study, and performed a complete follow-up. Conversely, most cohorts did not state the strategies to deal with confounding factors and did not measure the outcomes in a reliable way. Assessment of risk of bias in cohort and cross-sectional studies are summarized in Figure 2 and detailed in Supplementary Appendix S7.

### 3.4. Results of individual studies

#### 3.4.1. Markers of tumor progression, cell proliferation, immune and cell death regulation are among the most frequently assessed proteins

**Figure 3:**
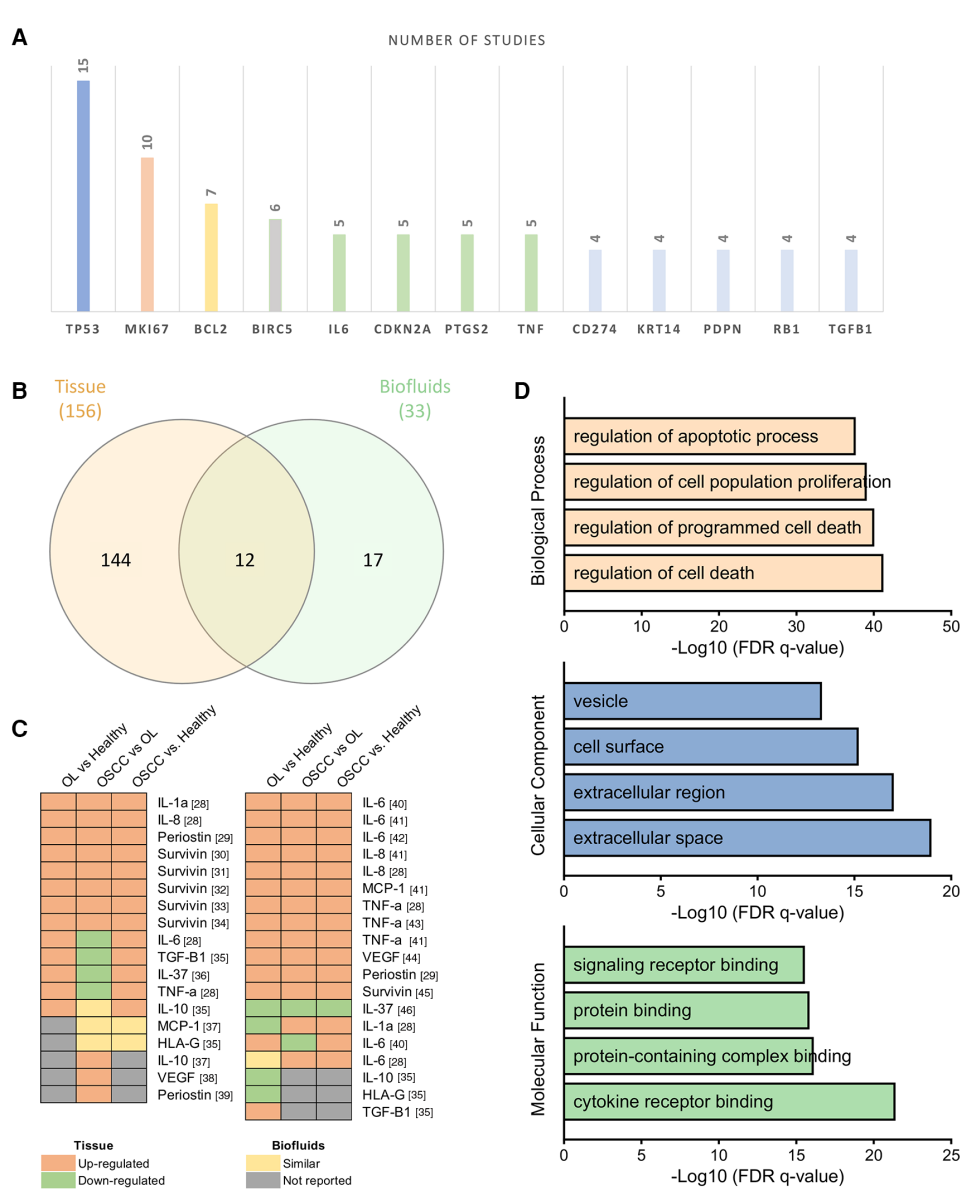
Analysis of assessed proteins among all included studies. (**A**) Top 13 genes evaluated in at least four distinct studies in descending order of frequency. (**B**) Venn diagram evidencing proteins exclusively assessed in tissue and biofluids samples, as well as in 12 shared proteins. (**C**) Heatmaps of the 12 shared proteins among tissues and biofluids representing the abundance pattern among the comparison pairs. In brackets the references. (**D**) Gene Ontology analysis performed by PANTHER highlights cell death regulation, cell proliferation, and apoptotic process among proteins mainly enriched in the extracellular space.

Most of the analyzed proteins (*n* = 116; 67%) were described in only one of the included studies. The proteins that were most frequently investigated were p53 and Ki-67, in 15 and 10 studies respectively (Figure 3A). Among tumor suppressors, p53, p16, and pRb were the most frequently assessed. The expression of protein p53 progressively increased from normal mucosa to OL and OSCC, suggesting that OL patients overexpressing p53 may have a higher risk of developing OSCC than those not expressing p53 (37, 47–51). On the other hand, p16 demonstrated linear decreased expression towards malignancy (47, 52, 53). Regarding pRb, which is also a tumor suppressor protein, some studies reported that most of the lesions stained positively (47, 49), whilst a reduction of pRb expression could be observed in transition from hyperplasia to dysplasia (*p* = 0.005) and from OL to OSCC (OR = 2.938, 95% CI: 1.568–5.504; *p* = 0.001) (52, 53).

Markers of cell proliferation (Ki-67, Survivin, and Keratin 14) were also frequently investigated as potential markers of malignant transformation. The expression of Ki-67 progressively increased from hyperplasia to dysplasia and OSCC, and in OSCC increased according to loss of differentiation (36, 47, 49, 54–56). The proteins survivin and Keratin 14 also seemed statistically different among OSCC, OL, and normal mucosa, usually overexpressed in OSCC (*p* < 0.01) (28, 41, 42, 56–58).

Immune system regulators (PD-L1, COX-2, and IL-6), have also been constantly analyzed in the process of initiation and progression of OSCC. Regarding PD-L1, IHC density was significantly stronger in both OSCC, and OL compared with normal control (*p* < 0.0001) (59). Also, whether compared to healthy controls, transformed OL presented a 60-fold PD-L1 overexpression (*p* = 0.04) that was related to malignant transformation (*p* = 0.03) (60). Similarly, a significant increase in COX-2 expression could be observed from normal epithelium through OL to OSCC (*p* < 0.001) in tissue samples (61–64). The expression of IL-6, on the other hand, was mainly assessed in biofluids, revealing that salivary concentration of IL-6 was higher in OSCC patients than in controls and in non-dysplastic OL (*p* = 0.0012) (45, 65, 66).

Finally, cell death regulators (Bcl-2 and TNF-α), growth and differentiation regulator (TGF-β1), and cell migration and adhesion marker (Podoplanin) were also repeatedly evaluated, in at least four studies. A detailed characterization of each assessed protein is presented in Supplementary Appendix S8.

#### 3.4.2. Biofluids and tissue samples share potential markers related to immune regulation, cell proliferation, and tumor progression

Among the 142 included studies, 124 evaluated protein abundance in formalin-fixed paraffin-embedded tissues whereas 21 evaluated protein expression in biofluid samples, including 9 from serum, 11 from saliva, and 2 from both. Some studies evaluated the same proteins both in tissue and biofluid samples (45, 67, 68).

The profile 144 proteins were exclusively assessed on tissue samples, and 17 proteins were exclusive from biofluids samples (serum and saliva), whereas 12 proteins were evaluated in both tissue and biofluid samples (Figure 3B). Shared proteins were cytokines involved in immune system regulation (IL-1A, IL-6, IL-8, IL-10, IL-37, MCP-1, and TNF-α), defense/immunity proteins (HLA-G), growth factors (VEGF-A and TGF-β1), as well as cell adhesion molecules (Periostin), and cell proliferation promoters (Survivin).

Interestingly, IL-1A, IL-8, TNF-α, VEGF-A, Periostin, and Survivin presented similar abundance pattern by comparing the IHC analysis and the biofluids assessment. In contrast, IL-37 tissue abundance was higher in OL and OSCC than in normal tissue (*p* < 0.001), but serum concentration was decreased in OSCC patients than in OL and controls (*p* < 0.0001) (69, 70). On the other hand, IL-6 and MCP-1 presented significant differences among the groups only on saliva assessment by a sensitive bead-based multiplex immunoassay, whereas IL-10, HLA-G, and TGF-β1 presented significant differences among the groups only by IHC analysis (Figure 3C).

#### 3.4.3. Assessed proteins are mainly expressed in the extracellular region and are involved in regulation of cell death, cell proliferation, and apoptosis

Gene Ontology (GO) enrichment analysis performed on PANTHER (71) demonstrated that the biological processes mainly involved with the set of 173 proteins assessed on this review were regulation of cell death (*q*-value = 5.8 × 10^−42^) and regulation of cell proliferation (*q*-value = 8.31 × 10^−40^) (Figure 3D). The top four biological processes ranked by adjusted *p*-value may be modulated by 110 of the 173 evaluated proteins and 54 genes were present in all top four biological processes (Supplementary Appendix S9).

Assessment of the cellular components revealed that the assessed proteins are mainly expressed in the extracellular space and extracellular region (*q*-value = 1.04 × 10^−19^ and 9.20 × 10^−19^, respectively), cell surface (*q*-value = 5.98 × 10^−16^), and vesicles (*q*-value = 4.70 × 10^−14^), demonstrating that most of these proteins may be secreted and possibly enriched in secretome. In terms of molecular function, all identified proteins were mainly involved in cytokine receptor binding (*q*-value = 3.91 × 10^−22^), protein and protein-containing complex binding (*q*-value = 7.50 × 10^−17^) and signaling receptor binding (*q*-value = 2.80 × 10^−16^) (Figure 3D).

#### 3.4.4. Several evaluated genes present clinical correlation in TCGA and are HNSCC driver genes

Analysis of the association between mRNA expression of the evaluated proteins and clinicopathological features was performed using public information retrieved from TCGA. The analysis was performed with the list of 173 assessed genes in all included studies, and seventy-seven genes (44.5%) presented at least one significant association between protein abundance with TCGA clinical data (*p*-value ≤ 0.05) (Supplementary Appendix S10). The clinical characteristics that presented the greatest number of associated genes were neoplasm histological grade (19/173), pathological T grouped (14/173), and pathological N Status (11/173).

By searching for head and neck cancer driver genes, there was a total of 62 driver genes which were identified throughout 4 cohorts and 691 samples (25). When comparing the list of 62 driver genes for head and neck cancer with the list of 173 proteins identified in present systematic review, it was observed that 10 genes are common for both sets, namely: *B2M*, *CDKN2A, HRAS, MAPK1, NOTCH1, RB1, SMAD4, TGFBR2, TP53,* and *TP63*. Crosschecking the results of biological process enrichment with the TCGA analysis as well as driver genes assessment, it could be observed that all ten head and neck cancer driver genes are among the top four biological enriched processes, and also presented clinical association in TCGA data, revealing their potential as biomarkers.

### 3.5. Results of syntheses

**Figure 4:**
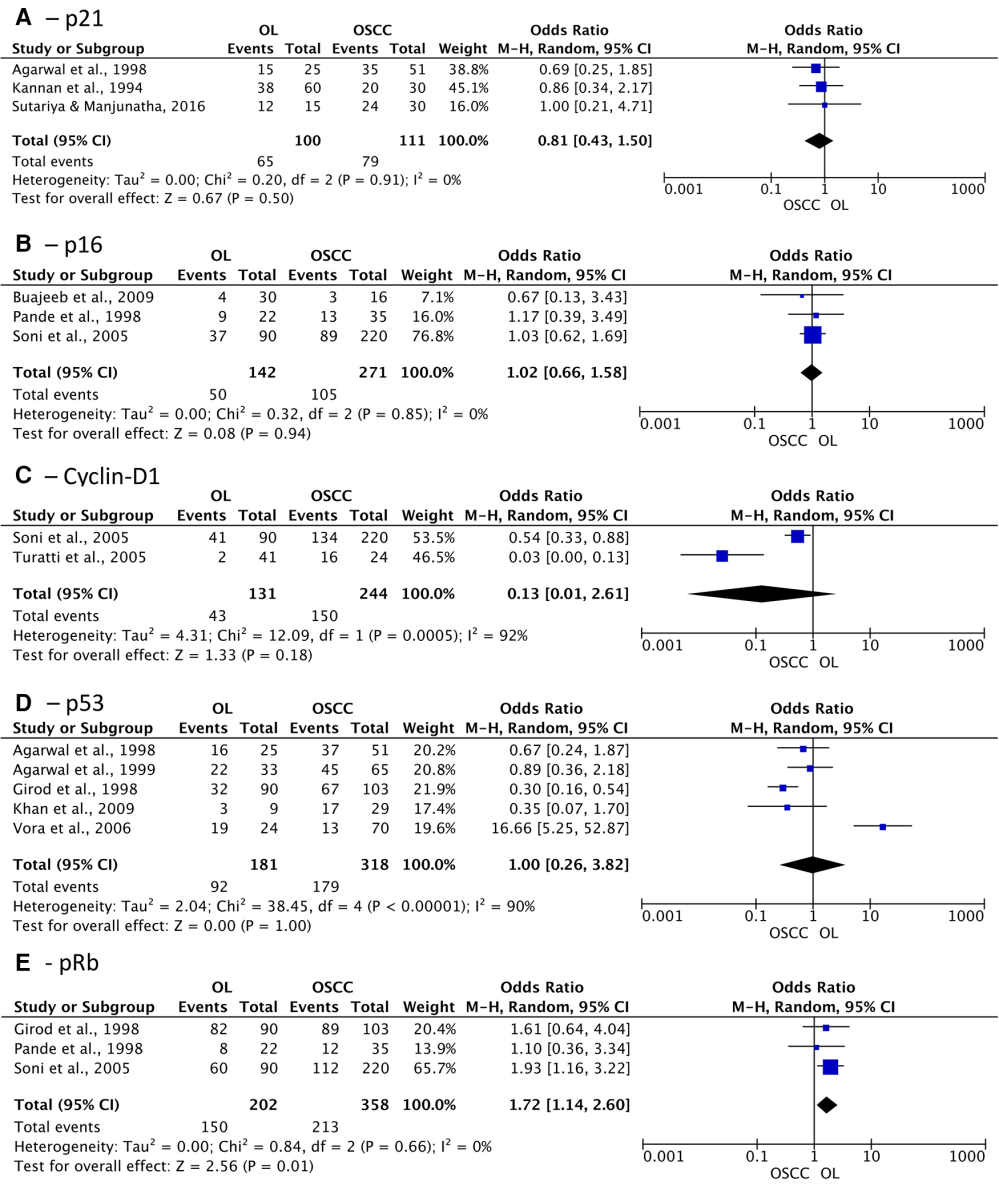
Association of expression of tumor suppressor proteins in oral leukoplakia (OL) compared to oral squamous cell carcinoma (OSCC). (**A**) p21; (**B**) p16; (**C**) Cyclin-D1; (**D**) p53; (**E**) pRb.

Among the proteins identified in the included studies, 18 proteins could be combined in association meta-analysis according to their main tumoral function. A total of 35 studies were included in the quantitative analysis, compiling the data of 1,714 OSCC patients and 1,369 OL individuals. The odds ratio (OR) analysis showed no difference between OL and OSCC groups in the abundance of tumor suppressor proteins p21, p16, Cyclin-D1, and p53 (Figures 4A–D). On the other hand, significant difference was observed among OL and OSCC patients in terms of positivity of pRb (OR: 1.72; 95% CI: 1.14 to 2.60; *I*^2^ = 0%; *p* = 0.010) (Figure 4E).

**Figure 5:**
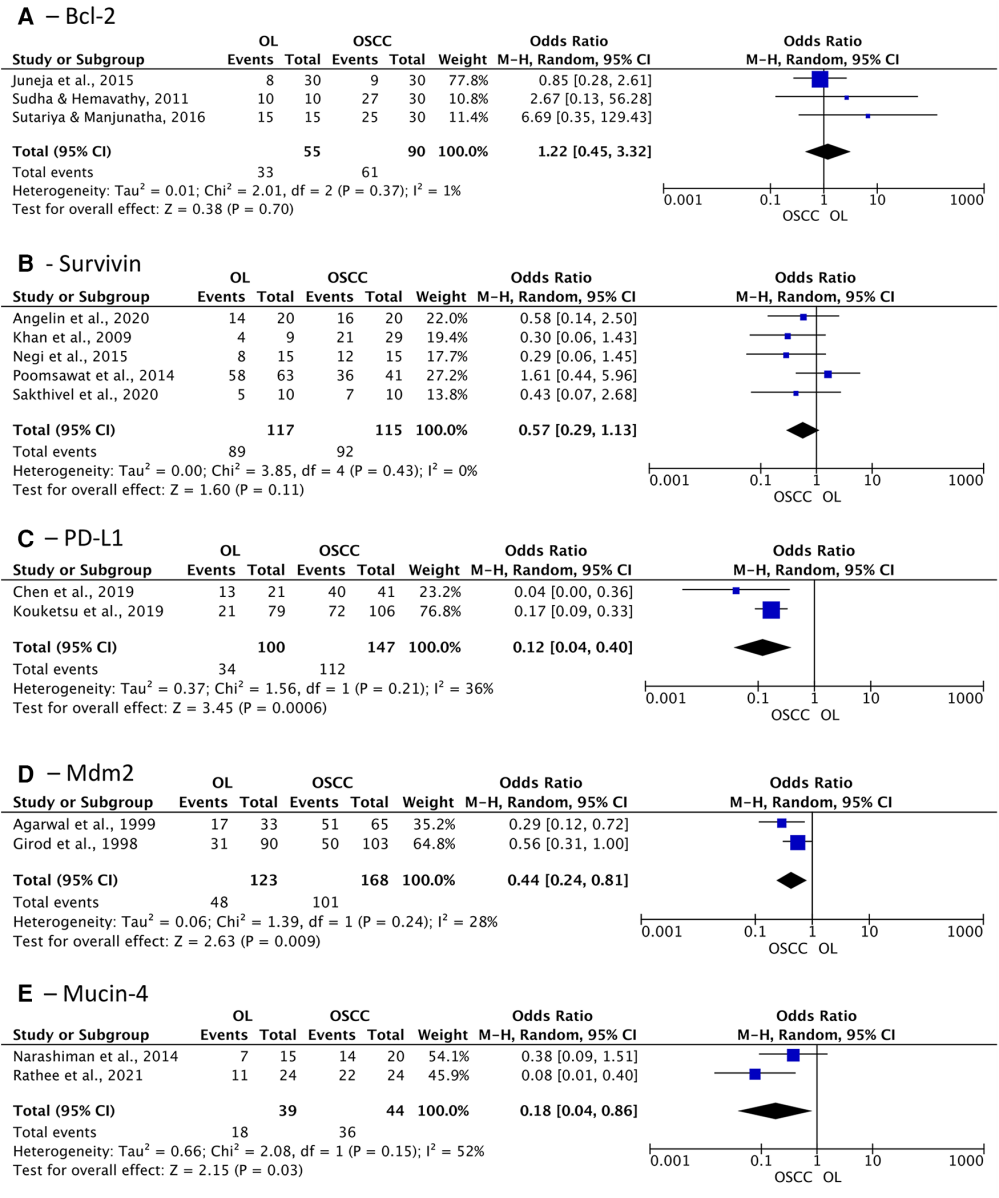
Association of expression of apoptosis suppressor proteins and tumor progression related proteins in oral leukoplakia (OL) compared to oral squamous cell carcinoma (OSCC). (**A**) Bcl-2; (**B**) Survivin; (**C**) PD-L1; (**D**) Mdm2; (**E**) Mucin-4.

Regarding apoptosis suppressor proteins, Bcl-2 and survivin did not show differences among the groups as well (OR: 1.22 (95% CI: 0.45 to 3.32; *p* = 0.70) and 0.57 (95% CI: 0.29 to 1.13; *p* = 0.15), respectively) (Figures 5A,B). Nonetheless, the expression of proteins related to tumor progression, PD-L1, Mdm2, and Mucin-4 demonstrated significant association with greater positivity in patients diagnosed with OSCC OR of 0.12 (95% CI: 0.04 to 0.40; *I*^2 ^= 36%; *p* = 0.0006), 0.44 (95% CI: 0.24 to 0.81; *I*^2 ^= 28%; *p* = 0.009), and 0.18 (95% CI: 0.04 to 0.86; *I*^2 ^= 52%; *p* = 0.03) respectively (Figures 5C–E).

**Figure 6:**
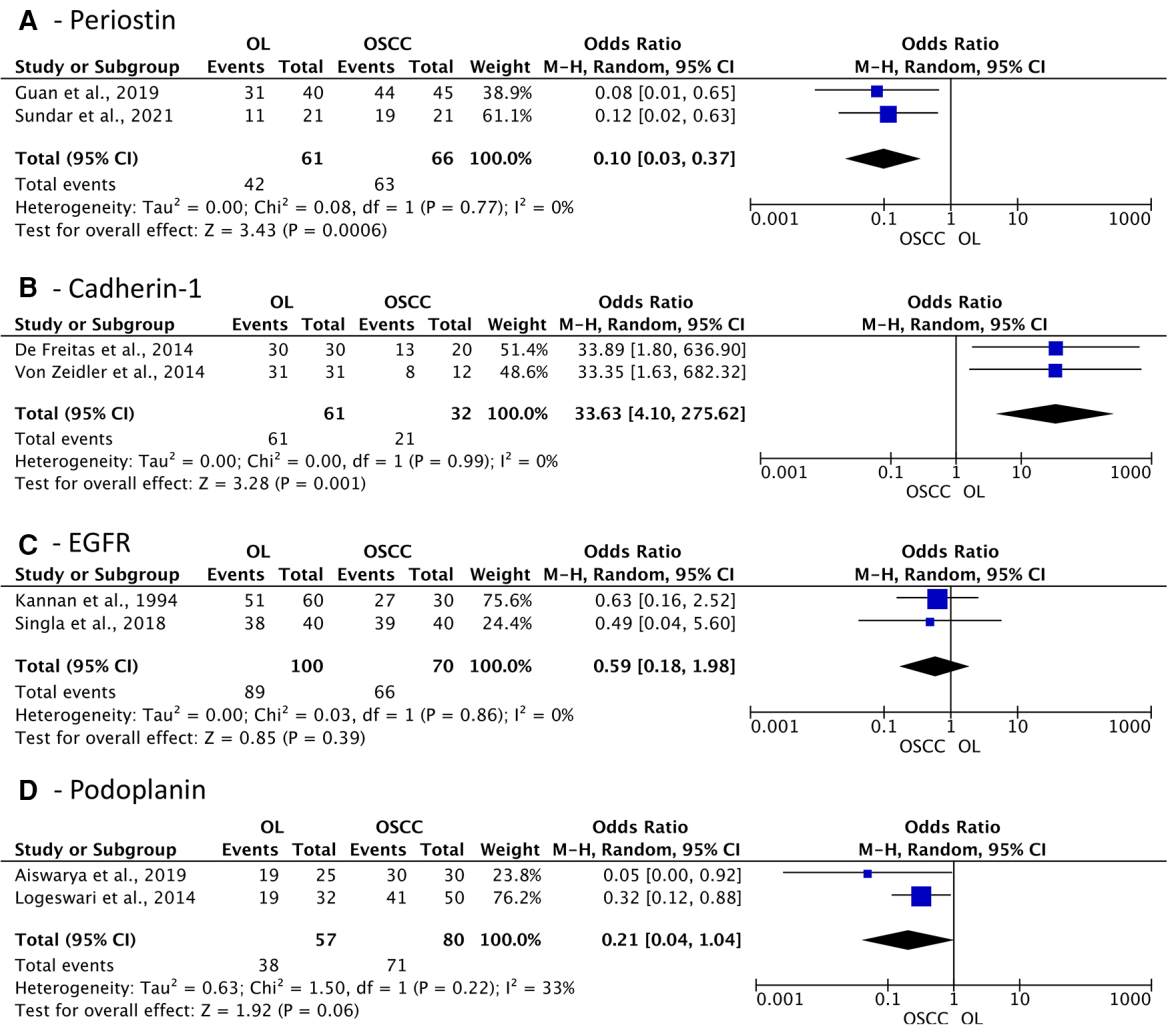
Association of expression of cell migration and cell adhesion proteins in oral leukoplakia (OL) compared to oral squamous cell carcinoma (OSCC). (**A**) Periostin; (**B**) Cadherin-1; (**C**) EGFR; (**D**) Podoplanin.

The association meta-analysis comparing cell migration and cell adhesion proteins demonstrated a significant difference between OL and OSCC patients in terms of periostin expression (OR: 0.10; 95% CI: 0.03 to 0.37; *I*^2^ = 0%; *p* = 0.0006) and Cadherin-1 positivity (OR: 33.63; 95% CI: 4.10 to 275.62; *I*^2^ = 0%; *p* = 0.001) (Figures 6A,B). On the other hand, Epidermal growth factor receptor (EGFR) and podoplanin did not show any difference between the groups, with an OR of 0.59 (*p* = 0.39) and 0.21 (*p* = 0.06), respectively.

**Figure 7:**
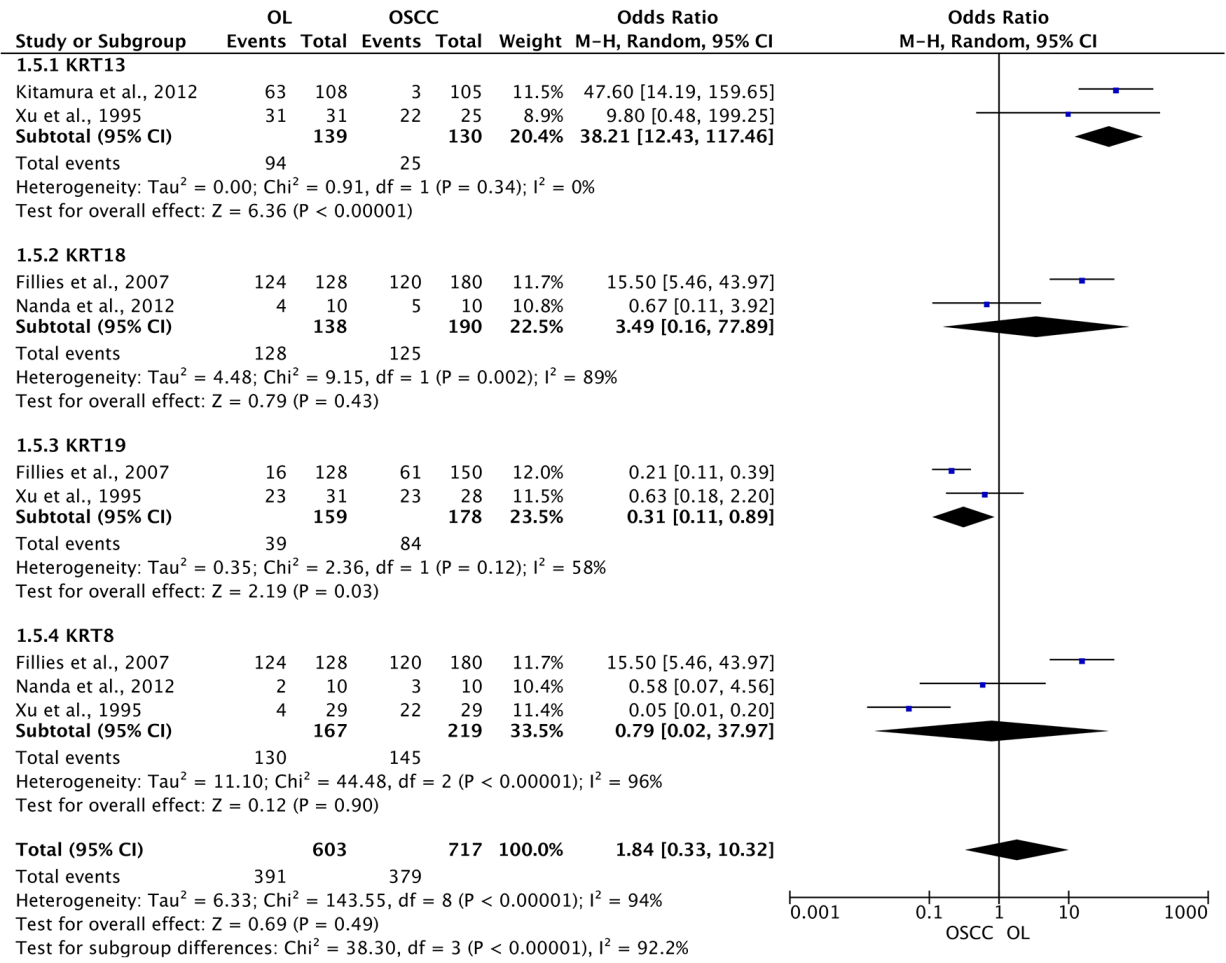
Association of expression of cytokeratins in oral leukoplakia (OL) compared to oral squamous cell carcinoma (OSCC).

Finally, cytokeratins (CK) 8, 13, 18, and 19 were pooled in subgroup analysis, revealing that collectively these genes do not show positivity difference, although separately CK-13 and CK-19 have shown significant difference between groups, with an OR of 38.21 (95% CI: 12.43 to 117.46; *I*^2 ^= 0%; *p* < 0.00001) and 0.31 (95% CI: 0.11 to 0.89; *I*^2 ^= 58%; *p* = 0.03), respectively (Figure 7). This result demonstrates that not all CKs are suitable for distinguishing OSCC from OL, and CK-13 and CK-19 may represent better options.

### 3.6. Certainty of evidence

The certainty of evidence from outcomes assessed by the meta-analysis were analyzed on GRADE system. For the association outcomes, moderate certainty of evidence was observed for the expression of pRb, Cadherin-1, Mdm2, p16, PD-L1, Podoplanin, Mucin-4, and periostin (Supplementary Appendix S11). This result reveals that further research may have an important impact on the confidence in the estimate of effect and could change the current results.

On the other hand, low certainty of evidence was demonstrated for the expression of survivin, p21, EGFR, and Bcl-2. For the expression of p53, cytokeratins, and Cyclin-D1 there was a very low certainty of evidence. Low and very low certainty of evidence were observed for these outcomes especially due to high risk of bias, considerable inconsistency, and consistent imprecision, revealing that further research will probably have an important impact on the confidence in the estimate of effect and is very likely to change it.

## 4. Discussion

Oral leukoplakia is among the most prevalent OPMD, with a mean rate of malignant transformation of approximately 15% (11). The disorder is still currently diagnosed by histopathological analysis, although microinvasive carcinoma may already be diagnosed at the initial assessment (4). Thus, it is of paramount importance to understand the molecular biology underneath this disorder and reveal biological markers of malignant transformation that may assist on future clinical assessment. The presence and grade of dysplasia is an important predictive marker to assess the risk for malignant transformation although not sufficiently accurate to define treatment and follow-up (11). Therefore, the search for protein markers that could predict the risk of malignant transformation could be critical for early treatment, improved survival, and decreased morbidity. To the best of our knowledge, this is the first systematic review with an association meta-analytical approach that aimed to assess protein biomarkers potentially involved with the malignant transformation of OL. Moreover, this review provided a list of protein candidates for further verification and validation assays.

Much effort has been done to summarize the literature regarding biomarkers of OL, OPMD, and dysplasia (8, 10, 17). Mello et al. (2020) (10) assessed general prognostic biomarkers in biopsy tissues of overall OPMD and found that the most evaluated proteins were p53, Ki-67, podoplanin, and p16, similar to our review. Also, they observed that studies investigating podoplanin reported a significant association between positive/high immunoexpression and malignant transformation, which was also observed in the studies assessing OL exclusively (72–74). Rivera et al. (2020) (8) also found podoplanin as one of the most assessed biomarkers of progression of dysplasia to oral cancer, as well as Aldehyde dehydrogenase 1A1 which were revealed to be associated with OSCC features according to TCGA as demonstrated by our systematic review. Monteiro et al. (2020) (17) performed a review of tissue biomarkers capable of predicting the risk of oral cancer in OL patients and found among 46 studies that the most assessed proteins were again podoplanin and p53. Podoplanin is a mucin-type transmembrane glycoprotein that mediates cell migration and adhesion, and its expression has been reported in squamous cell carcinomas and dysplastic lesions, suggesting that podoplanin may play a role in early oral tumorigenesis and in the malignant transformation (38, 75). Thus, further assessment of this protein, especially in other types of samples, are encouraging.

Assessing the proteins analyzed in at least four of the included studies, we provided a set of 13 proteins that have been majority investigated, including tumor suppressor proteins such as p53, pRb, and p16. The level of p53 has already been proven to progressively increase from normal mucosa to OL, to OSCC, following a progression toward malignancy (33, 48, 50, 51). Regarding pRb, which is also a tumor suppressor protein, some studies reported that most of the lesions stained positively (47, 49), whilst a reduction of pRb expression could be observed in transition from hyperplasia to dysplasia (*p* = 0.005) and from OL to OSCC (OR = 2.938, *p* = 0.001) (52, 53). Conversely, p16 expression linearly decreased from normal mucosa to dysplastic OL and to OSCC (47, 52, 53).

Cell proliferation proteins (Ki-67, CK-14, and Survivin) have also been extensively investigated in OL patients compared to oral cancer samples. It has been demonstrated that Ki-67 expression gradually increased through normal, non-dysplastic and dysplastic OL, and OSCC (36, 54, 55). The expression of Survivin and CK-14 also seemed statistically different among OSCC, OL, and normal mucosa, usually overexpressed in OSCC (*p* < 0.01) (28, 41, 42, 56–58).

Finally, immune system related proteins (IL-6, PD-L1, and COX-2), cell death regulators (Bcl-2 and TNF-α) as well as proteins associated with growth regulation (TGF-β1) and cell migration/adhesion (Podoplanin) were also repeatedly evaluated, in at least four studies. PD-L1, which is a protein encoded by the *CD274* gene, is overexpressed by numerous tumor cells as a strategy to evade immune responses (60). Stronger PD-L1 level was observed in both OSCC, and OL compared to normal control (*p* < 0.0001). In transformed OL, a significant 60-fold overexpression (*p* = 0.04) and in OSCC a 99.4-fold increase of PD-L1 level was detected and related to malignant transformation (*p* = 0.03) (59, 60). Regarding cell death regulators, TNF-α salivary concentration was found to be higher in OSCC patients than in controls (*p* = 0.0012) and non-dysplastic OL (*p* = 0.0492), although linear increase in salivary levels from normal mucosa to OL to OSCC has been also observed (*p* < 0.01). These findings demonstrated that TNF-α may be used to monitor the malignant transformation from OL to OSCC (45, 65, 76).

Cancer is characterized by abnormal and uncontrolled cellular growth primarily caused by genetic mutations known as “drivers” due to their ability to drive tumorigenesis. These mutations occur in a set of genes known as “cancer driver genes”, which consequently affect homeostatic key cellular functions (25). Among the 173 proteins assessed in the review, ten proteins were characterized as head and neck SCC driver genes, revealing the growing interest in also analyzing these genes in the process of progression from OL to oral cancer. A signature including driver genes suggests that this set may be effective in detecting proteins that may play a fundamental role in cancer maintenance by regulating the expression of several other OSCC suppressor or promoter genes. Of interest, as obtained by TCGA, *CDKN2A, RB1*, and *TP53* genes expression was correlated with clinical and pathological characteristics. *CDKN2A, RB1*, and *TP53* not only are driver genes but also present correlation with alcohol and tobacco history, final vital status, and neoplasm histologic grade in OSCC. Furthermore, all these three tumor suppressor genes could be pooled in association meta-analysis but only *RB1* positivity was significantly associated with OL compared to OSCC. Although *B2M, MAPK1,* and *SMAD4,* which are also driver genes, could not be pooled in the meta-analysis, they correlated with neoplasm histologic grade, pathologic stage and N status, final vital status, and HPV status in OSCC as demonstrated by TCGA clinical and pathological correlation analysis.

Taken altogether, analyzing protein levels to determine the susceptibility for transformation of potentially malignant oral lesions seems to be a promising method for potential risk determination and personalized management planning. Most of the studies included in the present systematic review evaluated protein expression primarily from IHC methods in FFPE tissues, which agrees with other previously published studies (10). Only 22 studies evaluated proteins in biofluids from patients with leukoplakia and OSCC. This result suggests that these proteins may compose the subproteome and may be suitable for assessment *via* biological fluids despite having their expression evaluated mainly in tissues. Given the ease of collection and the abundance of proteins in biofluids, it is still necessary to explore these types of samples in terms of progression risk, prognosis, and diagnosis feasibility. Interestingly, despite few studies evaluating protein expression in biofluids, most proteins were enriched in the extracellular space, in vesicles, or on the cell surface, demonstrating that these proteins may probably be quantified in biofluids. This would be advantageous as risk stratification and could be performed from simple and minimally invasive sample collections.

### 4.1. Limitations

Some limitations of the present systematic review and the included studies should be pointed out. First, the high heterogeneity in terms of assessed proteins and types of samples hindered a more focused and in-depth analysis of each protein individually. Furthermore, methodological heterogeneity may have led to statistical heterogeneity, as well as limiting the inclusion of more studies in the meta-analysis. In some studies, it was difficult to determine the assessment of malignant transformation when this was not the main outcome, and many studies had to be excluded as they did not determine which potentially malignant disorders were evaluated or did not individualize the leukoplakia data. Also, many studies were assessed as having high risk of bias due to lack of methodological rigor including inadequate statistical analysis. Thus, further well-designed studies are still needed to assess the expression of proteins capable of predicting the risk of malignant transformation of leukoplakia into oral cancer, especially in biofluids.

### 4.2. Conclusion

Assessing proteins in samples of OL patients has been gradually increasing to determine similarities and disparities with oral cancer patients that may indicate an increased risk of malignancy. The present review presented a list of 173 proteins that have already been assessed among patients diagnosed with oral SCC and OL, mainly detected in FFPE tissues as well as in biofluids. From these proteins, PD-L1, Mdm2, and Mucin-4 were significantly associated with greater abundance in OSCC patients, with a moderate certainty of evidence. The proteomic approach allowed the characterization of a set of proteins that have been investigated across OSCC initiation and progression. Moreover, the transcriptional expression in tissue is associated with clinical characteristics relevant to determine the prognosis and aggressiveness of the disease. Thus, further verification and validation are strongly recommended for future clinical application.

## 5. Other information

### 5.1. Protocol and registration

The methods of this systematic review were established prior to the review commencement and the resulting protocol based on PRISMA-P (77) that was registered at the International Prospective Register of Systematic Reviews (PROSPERO) database under registration number CRD42020157561. Also, the present systematic review was reported according to the Preferred Reporting Items for Systematic Reviews and Meta-Analyses (PRISMA) checklist (26).
