# Supplementary material for: A meta-analysis reveals the protein profile associated with malignant transformation of oral leukoplakia
Source: Front Oral Health. 2023 Feb 27;4:1088022. doi: 10.3389/froh.2023.1088022 (PMC10008949; doi:10.3389/froh.2023.1088022)
Supplement: Supplementary file 1 [file Table1.docx]

**Supplementary Materials from the article “A meta-analysis reveals the protein profile associated with malignant transformation of oral leukoplakia”**

Summary

[Appendix 1. Search strategies with appropriated key words and results from each database. 2](#_Toc125542692)

[Appendix 2. Excluded studies and reasons for exclusion (n=134) 3](#_Toc125542693)

[Appendix 3. Reference list of included studies in the qualitative synthesis of the systematic review 10](#_Toc125542694)

[Appendix 4. Qualitative synthesis of protein expression profile among OSCC, OL, and control groups. 21](#_Toc125542695)

[Appendix 5. Summary of descriptive characteristics of included studies assessing proteins in tissues 31](#_Toc125542696)

[Appendix 6. Summary of descriptive characteristics of included studies assessing proteins in biofluids 47](#_Toc125542697)

[Appendix 7. Risk of bias assessed by the Joanna Briggs Institute critical appraisal tools 50](#_Toc125542698)

[Appendix 8. Characteristics of assessed proteins among included studies 57](#_Toc125542699)

[Appendix 9. Genes enriched in the top-4 (FDR q-value) biological processes, molecular functions, and cellular components. 67](#_Toc125542700)

[Appendix 10. Characteristics of assessed proteins among included studies presenting clinical correlation in the TCGA comparing OSCC and normal samples (n=77). 71](#_Toc125542701)

[Appendix 11. Grading Of Recommendation, Assessment, Development, And Evaluation (GRADE) evidence profile. 74](#_Toc125542702)

# **Appendix 1**. Search strategies with appropriated key words and results from each database.

| **Database** | **Keywords**  (Search date: September 4^th^, 2021) | **Results** |
| --- | --- | --- |
| EMBASE | ('biomarkers'/exp OR biomarkers OR 'biomarker'/exp OR biomarker OR 'biological markers'/exp OR 'biological markers' OR 'biological marker'/exp OR 'biological marker' OR 'biologic marker' OR 'biologic markers' OR 'marker'/exp OR marker OR markers OR 'peptide'/exp OR peptide OR 'peptides'/exp OR peptides OR 'proteins'/exp OR proteins OR 'protein'/exp OR protein OR polypeptides OR 'gene'/exp OR gene OR 'genes'/exp OR genes OR 'signature'/exp OR signature) AND ('leukoplakia'/exp OR leukoplakia OR 'oral leukoplakia'/exp OR 'oral leukoplakia' OR 'leukoplakic lesions' OR 'leukoplakic lesion' OR 'proliferative verrucous leukoplakia'/exp OR 'proliferative verrucous leukoplakia' OR 'erythroleukoplakia'/exp OR erythroleukoplakia OR leukoerythroplakia) AND ('squamous cell carcinoma of head and neck'/exp OR 'squamous cell carcinoma of head and neck' OR 'head and neck squamous cell carcinoma'/exp OR 'head and neck squamous cell carcinoma' OR 'oral squamous cell carcinoma'/exp OR 'oral squamous cell carcinoma' OR 'mouth neoplasms'/exp OR 'mouth neoplasms' OR 'oral cancer'/exp OR 'oral cancer' OR 'oral neoplasia' OR 'oral neoplasm' OR 'oscc' OR 'squamous cell carcinoma'/exp OR 'squamous cell carcinoma') | 1,413 |
| LILACS | (Leucoplasia Oral OR Leucoplasia Bucal OR leukoplakia) AND (Biomarcadores OR biomarkers OR proteínas OR proteins Peptídeos OR péptidos OR peptides) AND (Neoplasias Bucais OR Neoplasias de la Boca OR Mouth Neoplasms) - filter: “title, abstract, subject” | 11 |
| PubMed | ("Biomarkers"[Mesh] OR biomarker OR "biological markers" OR "biological marker" OR "biologic marker" OR "biologic markers" OR marker OR markers OR peptide OR peptides OR "Proteins"[Mesh] OR protein OR polypeptides OR gene OR genes OR signature) AND ("Leukoplakia"[Mesh] OR "oral leukoplakia" OR "Leukoplakic Lesions" OR "Leukoplakic Lesion" OR "proliferative verrucous Leukoplakia" OR erythroleukoplakia OR leukoerythroplakia) AND ("Squamous Cell Carcinoma of Head and Neck"[Mesh] OR "Head and neck Squamous Cell Carcinoma" OR "Oral Squamous Cell Carcinoma" OR "Mouth Neoplasms"[Mesh] OR "oral cancer" OR "oral neoplasia" OR "oral neoplasm" OR "OSCC" OR "squamous cell carcinoma") | 1,271 |
| Scopus | TITLE-ABS-KEY(biomarkers OR biomarker OR "biological markers" OR "biological marker" OR "biologic marker" OR "biologic markers" OR marker OR markers OR peptide OR peptides OR proteins OR protein OR polypeptides OR gene OR genes OR signature ) AND TITLE-ABS-KEY(leukoplakia OR "oral leukoplakia" OR "Leukoplakic Lesions" OR "Leukoplakic Lesion" OR "proliferative verrucous Leukoplakia" OR erythroleukoplakia OR leukoerythroplakia ) AND TITLE-ABS-KEY("Squamous Cell Carcinoma of Head and Neck" OR "Head and neck Squamous Cell Carcinoma" OR "Oral Squamous Cell Carcinoma" OR "Mouth Neoplasms" OR "oral cancer" OR "oral neoplasia" OR "oral neoplasm" OR "OSCC" OR "squamous cell carcinoma" ) | 1,342 |
| Web of Science | (Biomarkers OR biomarker OR "biological markers" OR "biological marker" OR "biologic marker" OR "biologic markers" OR marker OR markers OR peptide OR peptides OR Proteins OR protein OR polypeptides OR gene OR genes OR signature) AND (Leukoplakia OR "oral leukoplakia" OR "Leukoplakic Lesions" OR "Leukoplakic Lesion" OR "proliferative verrucous Leukoplakia" OR erythroleukoplakia OR leukoerythroplakia) AND ("Squamous Cell Carcinoma of Head and Neck" OR "Head and neck Squamous Cell Carcinoma" OR "Oral Squamous Cell Carcinoma" OR "Mouth Neoplasms" OR "oral cancer" OR "oral neoplasia" OR "oral neoplasm" OR "OSCC" OR "squamous cell carcinoma") – filter: “topic” | 831 |
| Google Scholar | (Biomarkers OR proteins) AND (Leukoplakia OR "oral leukoplakia") AND ("Oral Squamous Cell Carcinoma") | 100 |
| ProQuest | TI,AB(Biomarkers OR biomarker OR "biological markers" OR "biological marker" OR "biologic marker" OR "biologic markers" OR marker OR markers OR peptide OR peptides OR Proteins OR protein OR polypeptides OR gene OR genes OR signature) AND TI,AB(Leukoplakia OR "oral leukoplakia" OR "Leukoplakic Lesions" OR "Leukoplakic Lesion" OR "proliferative verrucous Leukoplakia" OR erythroleukoplakia OR leukoerythroplakia) AND TI,AB("Squamous Cell Carcinoma of Head and Neck" OR "Head and neck Squamous Cell Carcinoma" OR "Oral Squamous Cell Carcinoma" OR "Mouth Neoplasms" OR "oral cancer" OR "oral neoplasia" OR "oral neoplasm" OR "OSCC" OR "squamous cell carcinoma") | 290 |

# Appendix 2. Excluded studies and reasons for exclusion (n=134)

| References | Reasons for exclusion* |
| --- | --- |
| 1. AbdulMajeed, A.A., Dalley, A. J., & Farah, C. S. (2013). Loss of ELF3 immunoexpression is useful for detecting oral squamous cell carcinoma but not for distinguishing between grades of epithelial dysplasia. Annals of diagnostic pathology, 17(4), 331-340. | 4 |
| 1. Abhishek, S. N., Mubeen, K., Vijayalakshmi, K. R., Suman, B., Gayitri, H. C., & Anitha, M. (2012). Serum total protein, albumin and advanced oxidation protein products (AOPP)-implications in oral squamous cell carcinoma. *The Malaysian journal of pathology*, *34*(1), 47. | 12 |
| 1. Afifi S, Zahran F, Shaker O, et al. Sensitivity and Specificity of Serum and Salivary CYFRA21-1 in Detecting Malignant Changes in Oral Potentially Malignant Lesions (Diagnostic Accuracy Study). World J Dent 2021;12(3):200–207. | 3 |
| 1. Ajila, V., Babu, S., Shetty, V., Hegde, S., & Shenoy, S. (2019). Secretory leukocyte protease inhibitor in oral potentially malignant disorders and oral squamous cell carcinoma. Indian Journal of Medical and Paediatric Oncology, 40(4), 491. | 3 |
| 1. Al-dhohrah, T., Mashrah, M., Yao, Z., & Huang, J. (2016). Aberrant DKK3 expression in the oral leukoplakia and oral submucous fibrosis: a comparative immunohistochemical study. European journal of histochemistry: EJH, 60(2). | 8 |
| 1. Aljabri, M. Y. (2020). Evaluation of ΔNp63 Expression in Oral Epithelial Dysplasia and Oral Squamous Cell Carcinoma (Doctoral dissertation, State University of New York at Buffalo). | 4 |
| 1. Alves MG, Kodama MH, da Silva EZM, et al. Relative expression of KLK5 to LEKTI is associated with aggressiveness of oral squamous cell carcinoma. Transl Oncol. 2021;14(1):100970. doi:10.1016/j.tranon.2020.100970 | 8 |
| 1. Angiero, F., Berenzi, A., Benetti, A., Rossi, E., Del Sordo, R., Sidoni, A., ... & Dessy, E. (2008). Expression of p16, p53 and Ki-67 proteins in the progression of epithelial dysplasia of the oral cavity. Anticancer research, 28(5A), 2535-2539. | 4 |
| 1. Arvind Rao & Sripathi Rao. (2013). Estimation of blood levels of Serum ceruloplasmin and Serum copper as tumour markers in oral leukoplakia and oral malignancies. Research Journal of Pharmaceutical, Biological and Chemical Sciences 4(1):1-7 | 10 |
| 1. Babiuch, K., Kęsek, B., Kuśnierz-Cabala, B., Kaczmarzyk, T., & Chomyszyn-Gajewska, M. (2015). Evaluation of nf-κb dependent cytokine levels in saliva of patients with oral potentially malignant disorders and oral squamous cell carcinoma: PP058. Head & Neck, 37. | 9 |
| 1. Babiuch, K., Bednarczyk, A., Gawlik, K., Pawlica-Gosiewska, D., Kęsek, B., Darczuk, D., Stępień, P., Chomyszyn-Gajewska, M., & Kaczmarzyk, T. (2019). Evaluation of enzymatic and non-enzymatic antioxidant status and biomarkers of oxidative stress in saliva of patients with oral squamous cell carcinoma and oral leukoplakia: a pilot study. Acta odontologica Scandinavica, 77(6), 408–418. https://doi.org/10.1080/00016357.2019.1578409 | 8 |
| 1. Bagan L, Sáez GT, Tormos MC, Labaig-Rueda C, Murillo-Cortes J, Bagan JV. Salivary and serum interleukin-6 levels in proliferative verrucous leukoplakia. *Clin Oral Investig*. 2016;20(4):737-743. doi:10.1007/s00784-015-1551-z | 3 |
| 1. Banerjee, A. G., Bhattacharyya, I., & Vishwanatha, J. K. (2005). Identification of genes and molecular pathways involved in the progression of premalignant oral epithelia. Molecular cancer therapeutics, 4(6), 865-875. | 4 |
| 1. Baran, C. A., Agaimy, A., Wehrhan, F., Weber, M., Hille, V., Brunner, K., ... & Ries, J. (2019). MAGE-A expression in oral and laryngeal leukoplakia predicts malignant transformation. Modern Pathology, 32(8), 1068-1081. | 3 |
| 1. Benchekroun, M. T., Saintigny, P., Thomas, S. M., El-Naggar, A. K., Papadimitrakopoulou, V., Ren, H., ... & Lee, J. J. (2010). Epidermal growth factor receptor expression and gene copy number in the risk of oral cancer. Cancer prevention research, 3(7), 800-809. | 4 |
| 1. Bhattacharjee, A., Giri, S. (2017). Evaluation of KI67, HER2/NEU and CK-OSCAR immunoexpression in oral premalignant and malignant lesions. Head and Neck, 39(0), E81-E83 | 9 |
| 1. Bossi, P., Perrone, F., Serafini, M. S., Pruneri, G., Piazza, C., Licitra, L., & De Cecco, L. (2019). OC-042 Genomic characterization of oral premalignant lesions to identify high-risk molecular clusters. *Radiotherapy and Oncology*, 132, 22. | 9 |
| 1. Cao, W., & Chen, W. (2012). EZH2 promotes oral squamous cell carcinoma initiation and progression through epigenetic silencing of FHL1. Cancer Research 72(8 Supplement):1143-1143 | 9 |
| 1. Carenzo, A., Serafini, M. S., Roca, E., Paderno, A., Mattavelli, D., Romani, C., ... & Bossi, P. (2020). Gene Expression Clustering and Selected Head and Neck Cancer Gene Signatures Highlight Risk Probability Differences in Oral Premalignant Lesions. Cells, 9(8), 1828. | 3 |
| 1. Chandra S, Goel MM, Makker A, Nayak S, Shilpi P, Bhatia V. (2014). Lymphangiogenesis in oral squamous cell carcinoma and potentially malignant oral lesions. Int J Pharm Biol Sci, 5, 983-993. | 10 |
| 1. Chandran, G. J., BALARAM, P., Kannan, S., PILLAI, M. R., Nalinakumari, K. R., & NAIR, M. K. (1994). Immunohistochemical localization of epidermal growth-factor and its receptor in normal, premalignant and malignant oral-mucosa. International journal of oncology, 4(2), 503-508. | 8 |
| 1. Chaves, A. L. F., Silva, A. G., Maia, F. M., Lopes, G. F. M., de Paulo, L. F. B., Muniz, L. V., ... & Loyola, A. M. (2019). Reduced CD8+ T cells infiltration can be associated to a malignant transformation in potentially malignant oral epithelial lesions. Clinical Oral Investigations, 23(4), 1913-1919. | 3 |
| 1. Chaw, S. Y., Majeed, A. A., Dalley, A. J., Chan, A., Stein, S., & Farah, C. S. (2012). Epithelial to mesenchymal transition (EMT) biomarkers–E-cadherin, beta-catenin, APC and Vimentin–in oral squamous cell carcinogenesis and transformation. *Oral oncology*, *48*(10), 997-1006. | 4 |
| 1. Chen, Q., Luo, G., Li, B., & Samaranayake, L. P. (1999). Expression of p16 and CDK4 in oral premalignant lesions and oral squamous cell carcinomas: a semi‐quantitative immunohistochemical study. Journal of oral pathology & medicine, 28(4), 158-164. | 4 |
| 1. Chen, H. M., Kuo, M. Y. P., Lin, K. H., Lin, C. Y., & Chiang, C. P. (2003). Expression of cyclin A is related to progression of oral squamous cell carcinoma in Taiwan. *Oral oncology*, *39*(5), 476-482. | 4 |
| 1. Chen, Y., Chen, X., Liu, M., Tang, Z., & Wang, M. (2004). Significance of the apoptosis and expression of Ki-67 in leukoplakia and oral squamous cell carcinoma. *Medical Journal of Wuhan University*, *25*(4), 429-431. | 10 |
| 1. Chen, Y. S., Wang, J. T., Chang, Y. F., Liu, B. Y., Wang, Y. P., Sun, A., & Chiang, C. P. (2004). Expression of hepatocyte growth factor and c‐met protein is significantly associated with the progression of oral squamous cell carcinoma in Taiwan. *Journal of oral pathology & medicine*, *33*(4), 209-217. | 4 |
| 1. Chen, C., Méndez, E., Houck, J., Fan, W., Lohavanichbutr, P., Doody, D., ... & Schwartz, S. M. (2008). Gene expression profiling identifies genes predictive of oral squamous cell carcinoma. *Cancer Epidemiology and Prevention Biomarkers*, *17*(8), 2152-2162. | 4 |
| 1. Chen, Y. K., Huang, A. H. C., Cheng, P. H., Yang, S. H., & Lin, L. M. (2013). Overexpression of Smad proteins, especially Smad7, in oral epithelial dysplasias. *Clinical oral investigations*, *17*(3), 921-932. | 4 |
| 1. Cheng LH. Ubiquitin and malignant transformation of oral mucosa. Head & Neck: Journal for the Sciences and Specialties of the Head and Neck. 2001 Nov;23(11):972-8. | 8 |
| 1. Cheng, S. J., Liu, Y. C., Cheng, S. L., Lee, J. J., Chen, H. M., Chang, H. H., ... & Chiang, C. P. (2013). Expression of G α12 predicts progression and prognosis of oral squamous cell carcinomas in T aiwan. *Journal of oral pathology & medicine*, *42*(7), 565-569. | 4 |
| 1. Dafar, A., Siarov, A., Robledo-Sierra, J., Sundberg, J., De Lara, S., Giglio, D., Öhman, J., Hasséus, B. (2019). PD-1 expressing cells in leukoplakia and oral lichen planus. *J Oral Pathol Med*, 48(Suppl. 1):5–83. | 9 |
| 1. Dai, Y., Liu, Y., Huang, D., Yu, C., Cai, G., Pi, L., ... & Zhang, X. (2012). Increased expression of Rab coupling protein in squamous cell carcinoma of the head and neck and its clinical significance. *Oncology letters*, *3*(6), 1231-1236. | 7 |
| 1. Dalley, A. J., Pitty, L. P., Major, A. G., AbdulMajeed, A. A., & Farah, C. S. (2014). Expression of ABCG 2 and B mi‐1 in oral potentially malignant lesions and oral squamous cell carcinoma. *Cancer medicine*, *3*(2), 273-283. | 4 |
| 1. Darling, M.R., Hassan, A., McLean L.Psoriasin: A New Biomarker in the Identification of Cancer Risk in Oral Lesions. Available online at: https://www.oralhealthgroup.com/features/psoriasin-a-new-biomarker-in-the-identification-of-cancer-risk-in-oral-lesions/ | 9 |
| 1. Dhivyalakshmi, M., & Maheswari, T. U. (2014). Expression of salivary biomarkers–Alkaline phosphatase & lactate dehydrogenase in oral leukoplakia. Int J Chemtech Res, 6, 3014-18. | 8 |
| 1. Donís SP, González AP, Alves MGO, et al. MLH1, MSH2, MRE11, and XRCC1 in Oral Leukoplakia and Oral Squamous Cell Carcinoma. Appl Immunohistochem Mol Morphol. 2021;29(8):613-618. doi:10.1097/PAI.0000000000000929 | 8 |
| 1. Driemel, O., Murzik, U., Escher, N., Melle, C., Bleul, A., Dahse, R., ... & von Eggeling, F. (2007). Protein profiling of oral brush biopsies: S100A8 and S100A9 can differentiate between normal, premalignant, and tumor cells. *PROTEOMICS–Clinical Applications*, *1*(5), 486-493. | 3 |
| 1. Duś, I., Hałoń, A., Radwan-Oczko, M. (2016). The protein expression of SPARC in changes of the oral mucosa. Dental and Medical Problems, 53(1), 153 | 10 |
| 1. Ganjre, A. P., & Bagul, N. (2014). Estimation and Comparison of Oxidative Stress Marker Superoxide Dismutase and Glutathione Peroxidase in Oral Leukoplakia and Oral Squamous Cell Carcinoma. *Indian Journal of Stomatology*, *5*(2), 49. | 10 |
| 1. Gao, L. (2011). Expression and significance of p27 and jab1 proteins in oral squamous cell carcinoma (Order No. 10546105). Available from ProQuest Dissertations & Theses Global. (1874973854). Retrieved from https://www.proquest.com/dissertations-theses/expression-significance-p27-jab1-proteins-oral/docview/1874973854/se-2?accountid=8113 | 10 |
| 1. Girod SC, Krueger G, Pape HD. (1993). p53 and Ki 67 expression in preneoplastic and neoplastic lesions of the oral mucosa. *International journal of oral and maxillofacial surgery*, *22*(5), 285-288. | 3 |
| 1. Gleber-Netto, F. O., Yakob, M., Li, F., Feng, Z., Dai, J., Kao, H. K., ... & Wong, D. T. (2016). Salivary biomarkers for detection of oral squamous cell carcinoma in a Taiwanese population. *Clinical Cancer Research*, *22*(13), 3340-3347. | 4 |
| 1. Goel, G., Srinidhi, A., Joshi, D., Gupta, V., Kapoor, N. (2018). Expression of Ki-67 and p53 in premalignant and malignant squamous cell lesions of the oral cavity: Is it significant? Indian Journal of Pathology and Microbiology, 61(5), S51 | 10 |
| 1. Goyal, G. (2020). Comparison of Salivary and Serum Alkaline Phosphates Level and Lactate Dehydrogenase Levels in Patients with Tobacco Related Oral Lesions with Healthy Subjects-A Step Towards Early Diagnosis. Asian Pacific Journal of Cancer Prevention, 21(4), 983-991. | 3 |
| 1. Han, B. F., Li, L. J. (2010). Numb and its isoforms in the role of oral leukoplakia and squamous cell carcinoma. Available from ProQuest Dissertations & Theses Global. (1868827198). Retrieved from https://search.proquest.com/docview/1868827198?accountid=8113 | 10 |
| 1. Hassan, N. M. M., Hamada, J. I., Murai, T., Seino, A., Takahashi, Y., Tada, M., ... & Moriuchi, T. (2006). Aberrant expression of HOX genes in oral dysplasia and squamous cell carcinoma tissues. Oncology Research Featuring Preclinical and Clinical Cancer Therapeutics, 16(5), 217-224. | 13 |
| 1. Huang, H. J., Ping, F. Y., Hu, J. A., & Zhao, S. F. (2009). Expression of Notch1 and epidermal growth factor receptor in human tongue squamous cell carcinoma and precancerous lesion. *Hua xi kou qiang yi xue za zhi= Huaxi kouqiang yixue zazhi= West China journal of stomatology*, *27*(6), 665-8. | 10 |
| 1. Jham, B. C., Costa, N. L., Silva, J. M., de Miranda, A. C., Oliveira, J. C., Silva, T. A., & Batista, A. C. (2012). Midkine expression in oral squamous cell carcinoma and leukoplakia. *Journal of oral pathology & medicine*, *41*(1), 21-26. | 8 |
| 1. Kafil Akhtar, A. A., Siddiqui, S. A., & Sherwani, R. K. (2016). Transition of immunohistochemical expression of e-cadherin and vimentin from premalignant to malignant lesions of oral cavity and oropharynx. Oman medical journal, 31(3), 165. | 3 |
| 1. Kang Y, Chen J, Li X, et al. Salivary KLK5 and uPA are potential biomarkers for malignant transformation of OLK and OLP. Cancer Biomark. 2021;31(4):317-328. doi:10.3233/CBM-203105 | 10 |
| 1. Kannan S, Balaram P, Chandran GJ, et al. (1994). Differential expression of cytokeratin proteins during tumour progression in oral mucosa. *Epithelial Cell Biol*, 3(2), 61‐69. | 10 |
| 1. Kannan S, Balaram P, Chandran GJ, et al. (1994). Alterations in expression of basement membrane proteins during tumour progression in oral mucosa. *Histopathology*, 24(6), 531‐537. | 8 |
| 1. Kannan, S., Balaram, P., Chandran, J., Pillai, R., Mathew, B., Nalinakumari, K. R., & Nair, K. (1994). Alterations in expression of terminal differentiation markers of keratinocytes during oral carcinogenesis. *Pathobiology*, *62*(3), 127-133. | 8 |
| 1. Kannan R, Bijur GN, Mallery SR, et al. Transforming growth factor-alpha overexpression in proliferative verrucous leukoplakia and oral squamous cell carcinoma: an immunohistochemical study. *Oral Surg Oral Med Oral Pathol Oral Radiol Endod*. 1996;82(1):69-74. doi:10.1016/s1079-2104(96)80379-9 | 3 |
| 1. Karathanasi, V., Hatzistamou, I., & Sklavounou, A. (2012). TGFβ/Smad4 signaling in oral leukoplakia and squamous cell carcinoma: 5. *Oral Diseases*, *18*. | 9 |
| 1. Katarkar, A., Patel, L., Mukherjee, S., Ray, J. G., Haldar, P. K., & Chaudhuri, K. (2015). Association of oral tumor suppressor gene deleted in oral cancer-1 (DOC-1) in progression of oral precancer to cancer. Oral Science International, 12(1), 15-21. | 13 |
| 1. Kaur, J., Matta, A., Kak, I., Srivastava, G., Assi, J., Leong, I., ... & Walfish, P. G. (2014). S100A7 overexpression is a predictive marker for high risk of malignant transformation in oral dysplasia. *International journal of cancer*, *134*(6), 1379-1388. | 4 |
| 1. Kikegawa, A. (2001). Immunohistochemical analysis of the p53 tumor suppressor gene product in oral leukoplakia. *Kokubyo Gakkai zasshi. The Journal of the Stomatological Society, Japan*, *68*(1), 51-59. | 9 |
| 1. Kiyosue, T., Kawano, S., Matsubara, R., Goto, Y., Hirano, M., Jinno, T., ... & Nakamura, S. (2013). Immunohistochemical location of the p75 neurotrophin receptor (p75NTR) in oral leukoplakia and oral squamous cell carcinoma. *International journal of clinical oncology*, *18*(1), 154-163. | 8 |
| 1. Kövesi, G., & Szende, B. (2003). Changes in apoptosis and mitotic index, p53 and Ki67 expression in various types of oral leukoplakia. *Oncology*, *65*(4), 331-336. | 8 |
| 1. Kujan, O., Huang, G., Ravindran, A., Vijayan, M., & Farah, C. S. (2019). CDK4, CDK6, cyclin D1 and Notch1 immunocytochemical expression of oral brush liquid‐based cytology for the diagnosis of oral leukoplakia and oral cancer. Journal of Oral Pathology & Medicine, 48(7), 566-573. | 3 |
| 1. Kumar, S. K. S., Zain, R. B., Ismail, S. M., & Cheong, S. C. (2005). Human telomerase reverse transcriptase expression in oral carcinogenesis-a preliminary report. *Journal of Experimental and Clinical Cancer Research*, *24*(4), 639. | 10 |
| 1. Kuo, M. Y. P., Chang, H. H., Hahn, L. J., Wang, J. T., & Chiang, C. P. (1995). Elevated ras p21 expression in oral premalignant lesions and squamous cell carcinomas in Taiwan. *Journal of oral pathology & medicine*, *24*(6), 255-260. | 4 |
| 1. Kyrodimou, M., Andreadis, D., Drougou, A., Amanatiadou, E. P., Angelis, L., Barbatis, C., ... & Vizirianakis, I. S. (2014). Desmoglein-3/γ-catenin and E-cadherin/ß-catenin differential expression in oral leukoplakia and squamous cell carcinoma. *Clinical oral investigations*, *18*(1), 199-210. | 8 |
| 1. LI, Y., CHEN, J., & GENG, Y. H. (2010). Expressions of p57~(KIP2) protein in oral leukoplakia and oral squamous cell carcinoma and its clinical significance. *Journal of Practical Oncology*, (6), 19. | 10 |
| 1. Liao, J., Mitsuyasu, T., Yamane, K., & Ohishi, M. (2000). Telomerase activity in oral and maxillofacial tumors. *Oral oncology*, *36*(4), 347-352. | 8 |
| 1. Liu, Y. C., Ho, H. C., Lee, M. R., Yeh, C. M., Tseng, H. C., Lin, Y. C., & Chung, J. G. (2017). Cortactin is a prognostic marker for oral squamous cell carcinoma and its overexpression is involved in oral carcinogenesis. *Environmental toxicology*, *32*(3), 799-812. | 8 |
| 1. Lodi, G., Franchini, R., Bez, C., Sardella, A., Moneghini, L., Pellegrini, C., ... & Carrassi, A. (2010). Detection of survivin mRNA in healthy oral mucosa, oral leucoplakia and oral cancer. Oral diseases, 16(1), 61-67. | 13 |
| 1. Luzar, B., Poljak, M., Marin, I. J., Eberlinc, A., Klopčič, U., & Gale, N. (2004). Human telomerase catalytic subunit gene re‐expression is an early event in oral carcinogenesis. *Histopathology*, *45*(1), 13-19. | 4 |
| 1. Ma, L. W., Zhou, Z. T., He, Q. B., & Jiang, W. W. (2012). Phosphorylated p120-catenin expression has predictive value for oral cancer progression. *Journal of clinical pathology*, *65*(4), 315-319. | 3 |
| 1. Ma, L. W., Zhou, Z. T., He, Q. B., & Jiang, W. W. (2013). Phospholipase C‐γ1 expression correlated with cancer progression of potentially malignant oral lesions. *Journal of oral pathology & medicine*, *42*(1), 47-52. | 3 |
| 1. Malar, Dineshkumar, T., Nandhini, G., Ramya, R., Rameshkumar, A., Vijayalakshmi, S., & Rajkumar, K. (2019). Expression of Salivary Secretory Leukocyte Protease Inhibitor in Smokers, Leukoplakia and OSCC. Journal of Clinical & Diagnostic Research, 13(9). | 8 |
| 1. Metgud, R., & Patel, S. (2014). Serum and salivary levels of albumin as diagnostic tools for oral pre-malignancy and oral malignancy. *Biotechnic & Histochemistry*, *89*(1), 8-13. | 8 |
| 1. Mirhashemi, M., Ghazi, N., Saghravanian, N., Taghipour, A., & Mohajertehran, F. (2020). Evaluation of CD24 and CD44 as cancer stem cell markers in squamous cell carcinoma and epithelial dysplasia of the oral cavity by q-RT-PCR. Dental Research Journal, 17(3), 208. | 13 |
| 1. Mishra, R., & Das, B. R. (2009). Cyclin D1 expression and its possible regulation in chewing tobacco mediated oral squamous cell carcinoma progression. *Archives of oral biology*, *54*(10), 917-923. | 3 |
| 1. Monteiro, L., Silva, P., Delgado, L., Amaral, J., Warnakulasuriya, S., Bousbaa, H. (2017). BUBR1 as a potential biomarker of malignant transformation in oral leukoplakias. Head & Neck 39(S1):E87 | 9 |
| 1. Monteiro, L., Delgado, L., Amaral, B., Salazar, F., Pacheco, J., Warnakulasuriya, S. Podoplanin as a potential predictive biomarker of malignant transformation in oral leukoplakias.  *J Oral Pathol Med*, 48(Suppl. 1):5–83. | 9 |
| 1. Mustafa, R., & Rajaraman, S. M. (2016). Study on translational medicine of RUNX3 expression in oral precancerous lesions and squamous cell carcinoma. *Journal of International Translational Medicine*, *4*(1), 36-40. | 4 |
| 1. Mutirangura, A., Supiyaphun, P., Trirekapan, S., Sriuranpong, V., Sakuntabhai, A., Yenrudi, S., & Voravud, N. (1996). Telomerase activity in oral leukoplakia and head and neck squamous cell carcinoma. *Cancer research*, *56*(15), 3530-3533. | 8 |
| 1. Natesan, S. C., Ramakrishnan, B. P., Krishnapillai, R., & Thomas, P. (2019). Immunohistochemical Expression of Fascin in Oral Epithelial Dysplasia and Oral Squamous Cell Carcinoma. World, 10(5), 341. | 4 |
| 1. Nayak, S., Goel, M. M., Makker, A., Bhatia, V., Chandra, S., Kumar, S., & Agarwal, S. P. (2015). Fibroblast growth factor (FGF-2) and its receptors FGFR-2 and FGFR-3 may be putative biomarkers of malignant transformation of potentially malignant oral lesions into oral squamous cell carcinoma. PLoS One, 10(10). | 3 |
| 1. Nayyar, A. S. (2012). Novel biochemical markers: Early detection and prevention of malignant transformation a pilot study. *Acta Medica Iranica*, 597-602. | 12 |
| 1. Odani, T., Ito, D., Li, M. H., Kawamata, A., Isobe, T., Iwase, M., & Nagumo, M. (2006). Gene expression profiles of oral leukoplakia and carcinoma: genome-wide comparison analysis using oligonucleotide microarray technology. *International journal of oncology*, *28*(3), 619-624. | 8 |
| 1. Ogbureke, K. U., Abdelsayed, R. A., Kushner, H., Li, L., & Fisher, L. W. (2010). Two members of the SIBLING family of proteins, DSPP and BSP, may predict the transition of oral epithelial dysplasia to oral squamous cell carcinoma. *Cancer: Interdisciplinary International Journal of the American Cancer Society*, *116*(7), 1709-1717. | 3 |
| 1. Okuno-Ishii, N. (1993). Analysis of expression of p53 tumor suppressor gene in human oral squamous cell carcinoma. *Kokubyo Gakkai zasshi. The Journal of the Stomatological Society, Japan*, *60*(1), 131-148. | 11 |
| 1. Pandey, M., Prakash, O., Santhi, W. S., Soumithran, C. S., & Pillai, R. M. (2008). Overexpression of COX-2 gene in oral cancer is independent of stage of disease and degree of differentiation. *International journal of oral and maxillofacial surgery*, *37*(4), 379-383. | 3 |
| 1. Patlolla P, N Shyam NDV, Kumar GK, Narayen V, Konda P, Mudududla P. Evaluation of glucose transporter-1 expression in oral epithelial dysplasia and oral squamous cell carcinoma: An immunohistochemical study. J Oral Maxillofac Pathol. 2020;24(3):578. doi:10.4103/jomfp.JOMFP_314_19 | 4 |
| 1. Prajwal, A., Kumar, A., & Chauhan, S. (2018). Role of ERBB receptor feedback inhibitor 1 (ERRFI1) downregulation in progression of premalignant oral lesions to cancer. *Journal Of Clinical Oncology,* 36(15). | 9 |
| 1. Probstmeier, R., Kraus, D., Wenghoefer, M., & Winter, J. (2019). S100 proteins as biomarkers in risk estimations for malignant transformation in oral lesions. In *Calcium-Binding Proteins of the EF-Hand Superfamily* (pp. 763-771). Humana Press, New York, NY. | 9 |
| 1. Punyani, S. R., & Sathawane, R. S. (2013). Salivary level of interleukin-8 in oral precancer and oral squamous cell carcinoma. *Clinical oral investigations*, *17*(2), 517-524. | 3 |
| 1. Ram, V. P., Nirmala, N. R., & Kotian, M. S. (2005). Immunohistochemical evaluation of expression of cytokeratin 19 in different histological grades of leukoplakia and oral squamous cell carcinoma. *Indian journal of dental research: official publication of Indian Society for Dental Research*, *16*(1), 6-11. | 10 |
| 1. Ries, J., Wehrhan, F., Baran, C., Agaimy, A., Danzer, E., Bolzel, S., ... & Weber, M. (2020, February). Expression of the Immune Modulators of the PD-1: PD-L1 Axis in oral Leukoplakia. Oncology Research And Treatment, 43, 99-100. | 9 |
| 1. Rodríguez, M. J., Acha, A., Ruesga, M. T., Rodríguez, C., Rivera, J. M., & Aguirre, J. M. (2007). Loss of expression of DNA repair enzyme MGMT in oral leukoplakia and early oral squamous cell carcinoma. A prognostic tool?. *Cancer letters*, *245*(1-2), 263-268. | 8 |
| 1. Rupa, J., Ramani, P., Premkumar, P., Natesan, A., & Sherlin, H. J. (2020). Expression of serum fucosyltransferase 8 in oral squamous cell carcinoma and potentially malignant disorder-A case-control study. Drug Invention Today, 14(7). | 3 |
| 1. Sabarathinam, J., Selvaraj, J., & Devi, S. (2019). Estimation of Levels of Glutathione Peroxidase (Gpx), Malondialdehyde (Mda), Tumor Necrosis Factor Alpha (Tnf Alpha) and Alpha Feto Protein (Afp) In Saliva of Potentially Malignant Disorders and Oral Squamous Cell Carcinoma. Biomedical and Pharmacology Journal, 12(04), 1881-1886. | 3 |
| 1. Sabarathinam, J., Dharman, S., & Selvaraj, J. (2020). Combination Assay for Tumor Markers in Saliva of Potentially Malignant Disorders and Oral Squamous Cell Carcinoma. Journal of Pharmaceutical Research International, 36-45. | 8 |
| 1. Safadi, R. A., Abdullah, N. I., Alaaraj, R. F., Bader, D. H., Divakar, D. D., Hamasha, A. A., & Sughayer, M. A. (2019). Clinical and histopathologic prognostic implications of the expression of cytokeratins 8, 10, 13, 14, 16, 18 and 19 in oral and oropharyngeal squamous cell carcinoma. Archives of Oral Biology, 99, 1-8. | 2 |
| 1. Santosh, N., McNamara, K. K., Beck, F. M., & Kalmar, J. R. (2019). Expression of cornulin in oral premalignant lesions. *Oral surgery, oral medicine, oral pathology and oral radiology*, *127*(6), 526-534. | 4 |
| 1. Saranath, D., Tandle, A. T., Deo, M. G., Mehta, A. R., & Sanghvi, V. (1997). Loss of p53 gene as a biomarker of high risk oral leukoplakias. *Indian journal of biochemistry & biophysics*, *34*(3), 266-273. | 10 |
| 1. Schaaij-Visser, T. B., Bremmer, J. F., Braakhuis, B. J., Heck, A. J., Slijper, M., van der Waal, I., & Brakenhoff, R. H. (2010). Evaluation of cornulin, keratin 4, keratin 13 expression and grade of dysplasia for predicting malignant progression of oral leukoplakia. *Oral oncology*, *46*(2), 123-127. | 2 |
| 1. Schoelch, M. L., Le, Q. T., Silverman Jr, S., McMillan, A., Dekker, N. P., Fu, K. K., ... & Regezi, J. A. (1999). Apoptosis-associated proteins and the development of oral squamous cell carcinoma. *Oral oncology*, *35*(1), 77-85. | 4 |
| 1. Schroeder, K. N., Pries, R., Wollenberg, B. (2009). Function and regulation of nucleotide-binding receptors in malignant head and neck cancer. European Journal of Immunology, 39(0), S78 | 9 |
| 1. Shah, M. H., Telang, S. D., Shah, P. M., & Patel, P. S. (2008). Tissue and serum α2-3-and α2-6-linkage specific sialylation changes in oral carcinogenesis. *Glycoconjugate journal*, *25*(3), 279-290. | 3 |
| 1. Shahnavaz, S. A., Regezi, J. A., Bradley, G., Dube, I. D., & Jordan, R. C. K. (2000). p53 gene mutations in sequential oral epithelial dysplasias and squamous cell carcinomas. *The Journal of Pathology: A Journal of the Pathological Society of Great Britain and Ireland*, *190*(4), 417-422. | 4 |
| 1. Shi, Y. J., Shen, L. J., & Yin, C. (2009). Expressions of receptor-interacting protein and caspase-8 in oral squamous cell carcinoma and oral precancerous lesions. *Nan fang yi ke da xue xue bao= Journal of Southern Medical University*, *29*(9), 1802-1805. | 11 |
| 1. Shiiba, M., Saito, K., Yamagami, H., Nakashima, D., Higo, M., Kasamatsu, A., ... & Tanzawa, H. (2015). Interleukin-1 receptor antagonist (IL1RN) is associated with suppression of early carcinogenic events in human oral malignancies. *International journal of oncology*, *46*(5), 1978-1984. | 8 |
| 1. Shintani, S., Yoshihama, Y., Emilio, A. R., & Matsumura, T. (1995). Overexpression of p53 is an early event in the tumorigenesis of oral squamous cell carcinomas. *Anticancer research*, *15*(2), 305-308. | 10 |
| 1. Silva, B. S. D. F. (2011). *Avaliação da expressão das proteínas Twist, Caderina-E, e p-Akt nos eventos que regem a progressão do carcinoma epidermóide oral* (Doctoral dissertation, Universidade de São Paulo). | 10 |
| 1. Singh, A. P., Kumar, N., Raju, M. S., Singh, N. N., & Nagendrareddy, S. G. (2014). Estimation of serum β2-microglobulin in potentially malignant disorders and squamous cell carcinoma of the oral cavity: A clinicopathological study. *Dental research journal*, *11*(1), 109. | 8 |
| 1. Sivadas, V. P., Saakshi, G., Iype, E. M., Balan, A., & Kannan, S. (2015). Prognostic implication of the loss of TGFBR2 expression in oral carcinoma. *Neoplasma*, *62*(3), 398-404. | 3 |
| 1. Sridharan, G., Ramani, P., & Patankar, S. (2017). Serum metabolomics in oral leukoplakia and oral squamous cell carcinoma. *Journal of cancer research and therapeutics*, *13*(3), 556. | 8 |
| 1. Sridharan, G., Ramani, P., Patankar, S., & Vijayaraghavan, R. (2019). Evaluation of salivary metabolomics in oral leukoplakia and oral squamous cell carcinoma. *Journal of Oral Pathology & Medicine*, *48*(4), 299-306. | 8 |
| 1. Sun, X. J., Ma, J., Zhang, H., Wang, X. K., & Li, J. H. (2005). The expressions of cyclooxygenase-2 (Cox-2), VEGF in oral squamous cell carcinoma and precancerous lesions and their significances. *Shanghai kou qiang yi xue= Shanghai journal of stomatology*, *14*(2), 173-176. | 11 |
| 1. Sun, G., & Ping, F. Y. (2009). Application of saliva protein fingerprints in the diagnosis of oral squamous cell cancer by surface enhanced laser desorption ionization time of flight mass. *Zhonghua kou qiang yi xue za zhi= Zhonghua kouqiang yixue zazhi= Chinese journal of stomatology*, *44*(11), 664-667. | 11 |
| 1. Tegginamani AS, Shivakumar VH, Kallarakkal TG, Ismail SM, Abraham MT, Bin Zamzuri AT. Analysis of octamer-binding transcription factor-4 expression in oral leukoplakia. J Oral Maxillofac Pathol. 2020;24(2):400. doi:10.4103/jomfp.JOMFP_272_19 | 2 |
| 1. Tosios, K. I., Kapranos, N., & Papanicolaou, S. I. (1998). Loss of basement membrane components laminin and type IV collagen parallels the progression of oral epithelial neoplasia. *Histopathology*, *33*(3), 261-268. | 3 |
| 1. Tripathi, S. C., Kumar, M., Kaur, J., Chauhan, S. S., Shukla, N. K., Thakkar, A., ... & Siu, K. M. (2011). Abstract B25: Emerging role of heterogeneous ribonucleoproteins (hnRNPs) as early predictive marker and prognosticator for head and neck oral squamous cell carcinoma. | 9 |
| 1. Tsuzuki, H., Fujieda, S., Sunaga, H., Narita, N., Tokuriki, M., & Saito, H. (2003). Expression of p27 and apoptosis in oral leukoplakia. *Anticancer research*, *23*(2B), 1265-1270. | 10 |
| 1. Vajaria, B. N., Patel, K. R., Begum, R., Patel, J. B., Shah, F. D., Shukla, S. N., & Patel, P. S. (2012). Glycoprotein electrophoretic patterns have potential to monitor changes associated with neoplastic transformation in oral cancer. *The International journal of biological markers*, *27*(3), 247-256. | 4 |
| 1. Vajaria, B. N., Patel, K. R., Begum, R., Patel, J. B., Shah, F. D., Joshi, G. M., & Patel, P. S. (2014). Salivary glyco-sialylation changes monitors oral carcinogenesis. *Glycoconjugate journal*, *31*(9), 649-659. | 3 |
| 1. Vered, M., Allon, I., & Dayan, D. (2009). Maspin, p53, p63, and Ki‐67 in epithelial lesions of the tongue: from hyperplasia through dysplasia to carcinoma. *Journal of oral pathology & medicine*, *38*(3), 314-320. | 3 |
| 1. Verma, V., & Chandrashekar, C. (2019). Evaluation of SOX2 and podoplanin expression in oral epithelial dysplasia and its correlation with malignant transformation. Journal of investigative and clinical dentistry, 10(4), e12450. | 2 |
| 1. Vigneswaran, N., Peters, K. P., Hornstein, O. P., Haneke, E. (1991). Immunostaining of β2-microglobulin provides an improved evaluation of risk of malignancy in oral leukoplakias. Skin Cancer, 6(4), 187-195. | 10 |
| 1. Wang, G. S., Wen, Y. M. (2000). Study on the relationship of cell apoptotic gene's expression and maligant change of leukoplakia. Available from ProQuest Dissertations & Theses Global. (1027131861). Retrieved from https://search.proquest.com/docview/1027131861?accountid=8113 | 10 |
| 1. Wang, D., Wen, S., & Wang, Z. (2019, October). T cells become exhausted in the process of malignant transformation of oral leukoplakia. European Journal Of Immunology, 49, 1389-1389. | 9 |
| 1. Warnakulasuriya, K. A. A. S., & Johnson, N. W. (1992). Expression of p53 mutant nuclear phosphoprotein in oral carcinoma and potentially malignant oral lesions. *Journal of oral pathology & medicine*, *21*(9), 404-408. | 4 |
| 1. Wei, Y., Liu, Z. W. (2011). Study on the expression of mcm-2p in oral leukopllakia and oral squamous cell carcinoma. Available from ProQuest Dissertations & Theses Global. (1873878505). Retrieved from https://search.proquest.com/docview/1873878505?accountid=8113 | 10 |
| 1. Wenghoefer, M., Pantelis, A., Dommisch, H., Reich, R., Martini, M., Allam, J. P., ... & Winter, J. (2008). Decreased gene expression of human β-defensin-1 in the development of squamous cell carcinoma of the oral cavity. International journal of oral and maxillofacial surgery, 37(7), 660-663. | 13 |
| 1. Wu, F., Shi, X., Zhang, R., Tian, Y., Wang, X., Wei, C., ... & Guo, W. (2018). Regulation of proliferation and cell cycle by protein regulator of cytokinesis 1 in oral squamous cell carcinoma. *Cell death & disease*, *9*(5), 1-12. | 8 |
| 1. Xia, J., Li, B. Q., Zeng, X., Chen, Q. M., He, Y., & Cai, Y. (2005). Amplification of EMS1 gene in oral carcinogenesis. *Zhonghua kou qiang yi xue za zhi= Zhonghua kouqiang yixue zazhi= Chinese journal of stomatology*, *40*(2), 102-104. | 11 |
| 1. Xiaonan, H. (2019). Expression levels of bdnf, vegf, il-17 and il-17f in oral and maxillofacial squamous cell carcinoma and their clinicopathological features. ACTA Medica Mediterranea, 35(3), 1225-1231. | 10 |
| 1. Ye, X., Zhang, J., Lu, R., & Zhou, G. (2016). Signal regulatory protein α associated with the progression of oral leukoplakia and oral squamous cell carcinoma regulates phenotype switch of macrophages. *Oncotarget*, *7*(49), 81305. | 8 |
| 1. Zhang, J., Zeng, Y., Zheng, J., & Xu, J. (2013). Expression of Prion protein and its clinical significance in oral squamous cells carcinoma and oral leukoplakia. *Zhonghua kou qiang yi xue za zhi= Zhonghua kouqiang yixue zazhi= Chinese journal of stomatology*, *48*(12), 752-754. | 11 |

*Reasons for exclusion:

1. Studies assessing biomarkers after any intervention for potentially malignant disorder or oral cancer treatment;
2. No comparison group;
3. Data not individualized for oral leukoplakia;
4. Does not specify the potentially malignant disorder;
5. Virus-associated biomarkers (HPV, HIV, EBV, HSV);
6. Hairy leukoplakia;
7. Non-oral leukoplakia;
8. No assessment of possible progression/ transformation;
9. Reviews, letter, conference abstracts, personal opinions, book chapter, in vitro or in vivo animal studies;
10. Full text copy not available;
11. Language restriction;
12. Studies containing data already reported in other studies;
13. No protein assessment.

Appendix 3. Reference list of included studies in the qualitative synthesis of the systematic review (n=142)

1. Agarwal S, Mathur M, Shukla NK, Ralhan R. Expression of cyclin dependent kinase inhibitor p21waf1/cip1 in premalignant and malignant oral lesions: relationship with p53 status. *Oral Oncol*. 1998;34(5):353-360. doi:10.1016/s1368-8375(98)00021-9
2. Agarwal S, Mathur M, Srivastava A, Ralhan R. MDM2/p53 co-expression in oral premalignant and malignant lesions: potential prognostic implications. *Oral Oncol*. 1999;35(2):209-216. doi:10.1016/s1368-8375(98)00092-x
3. Aiswarya A, Suresh R, Janardhanan M, Savithri V, Aravind T, Mathew L. An immunohistochemical evaluation of podoplanin expression in oral leukoplakia and oral squamous cell carcinoma to explore its potential to be used as a predictor for malignant transformation. *J Oral Maxillofac Pathol*. 2019;23(1):159. doi:10.4103/jomfp.JOMFP_272_17
4. Ambatipudi S, Bhosale PG, Heath E, et al. Downregulation of keratin 76 expression during oral carcinogenesis of human, hamster and mouse. *PLoS One*. 2013;8(7):e70688. doi:10.1371/journal.pone.0070688
5. Ameena M, Rathy R. Evaluation of tumor necrosis factor: Alpha in the saliva of oral cancer, leukoplakia, and healthy controls–A comparative study. *Journal of International Oral Health*. 2019; 11(2), 92.
6. Angelin D, Nair BJ. Comparative evaluation of survivin expression in leukoplakia, lichen planus, and oral squamous cell carcinoma: An immunohistochemical study. J Cancer Res Ther. 2020;16(3):569-574. doi:10.4103/jcrt.JCRT_421_19
7. Ankita K, Shwetha V, Vanitha S, Reddy Sujatha S, Nagaraju R, Tupakula Pavan K. Assessment of salivary endothelin-1 in patients with leukoplakia, submucous fibrosis, oral cancer and healthy individuals - a comparative study. *J Stomatol Oral Maxillofac Surg*. 2019;120(4):326-331. doi:10.1016/j.jormas.2019.02.024
8. Aruldoss N, Sarvathikari R, Srivastav CK, Austin RD. Expression of cyclo-oxygenase-2 enzyme in the tissue samples of patients with various clinicopathological stages of oral leukoplakia and oral squamous cell carcinoma. *Journal of Indian Academy of Oral Medicine and Radiology*. 2016;28(2), 134.
9. Babiuch K, Kuśnierz-Cabala B, Kęsek B, Okoń K, Darczuk D, Chomyszyn-Gajewska M. Evaluation of Proinflammatory, NF-kappaB Dependent Cytokines: IL-1α, IL-6, IL-8, and TNF-α in Tissue Specimens and Saliva of Patients with Oral Squamous Cell Carcinoma and Oral Potentially Malignant Disorders. *J Clin Med*. 2020;9(3):867. doi:10.3390/jcm9030867
10. Bavle RM, Paremala K, Soumya M, Reshma V, Sudhakara M. Immunohistochemical expression of p63 in oral premalignant disorders and its correlation with oral squamous cell carcinoma. *Journal of Datta Meghe Institute of Medical Sciences University*. 2020;15(2), 255.
11. Bernardes VF, Correa GT, Loyola AM, et al. STAG2 expression in oral cancer and potentially malignant lesions. *Tumour Biol*. 2014;35(4):3641-3645. doi:10.1007/s13277-013-1482-8
12. Brailo V, Vucicevic-Boras V, Lukac J, et al. Salivary and serum interleukin 1 beta, interleukin 6 and tumor necrosis factor alpha in patients with leukoplakia and oral cancer. *Med Oral Patol Oral Cir Bucal*. 2012;17(1):e10-e15. doi:10.4317/medoral.17323
13. Buajeeb W, Poomsawat S, Punyasingh J, Sanguansin S. Expression of p16 in oral cancer and premalignant lesions. *J Oral Pathol Med*. 2009;38(1):104-108. doi:10.1111/j.1600-0714.2008.00710.x
14. Chamorro-Petronacci CM, Lafuente-Ibanez De Mendoza I, Suarez-Peñaranda JM, et al. Immunohistochemical Characterization of Bcl-2 in Oral Potentially Malignant Disorders [published online ahead of print, 2021 May 17]. Appl Immunohistochem Mol Morphol. 2021;10.1097/PAI.0000000000000945. doi:10.1097/PAI.0000000000000945
15. Chang PY, Kuo YB, Wu TL, et al. Association and prognostic value of serum inflammation markers in patients with leukoplakia and oral cavity cancer. *Clin Chem Lab Med*. 2013;51(6):1291-1300. doi:10.1515/cclm-2012-0504
16. Chaudhari NT, Tupkari JV, Joy T, Ahire MS. Human MutL homolog 1 immunoexpression in oral leukoplakia and oral squamous cell carcinoma: A prospective study in Indian population. *J Oral Maxillofac Pathol*. 2016;20(3):453-461. doi:10.4103/0973-029X.190948
17. Chen XJ, Tan YQ, Zhang N, He MJ, Zhou G. Expression of programmed cell death-ligand 1 in oral squamous cell carcinoma and oral leukoplakia is associated with disease progress and CD8+ tumor-infiltrating lymphocytes. *Pathol Res Pract*. 2019;215(6):152418. doi:10.1016/j.prp.2019.04.010
18. de Freitas Silva BS, Yamamoto-Silva FP, Pontes HA, Pinto Júnior Ddos S. E-cadherin downregulation and Twist overexpression since early stages of oral carcinogenesis. *J Oral Pathol Med*. 2014;43(2):125-131. doi:10.1111/jop.12096
19. de Vicente JC, Donate-Pérez Del Molino P, Rodrigo JP, et al. SOX2 Expression Is an Independent Predictor of Oral Cancer Progression. *J Clin Med*. 2019;8(10):1744.
20. Deepthi G, Nandan SRK, Kulkarni PG. Salivary Tumour Necrosis Factor-α as a Biomarker in Oral Leukoplakia and Oral Squamous Cell Carcinoma. *Asian Pac J Cancer Prev*. 2019;20(7):2087-2093. doi:10.31557/APJCP.2019.20.7.2087
21. Dikova V, Jantus-Lewintre E, Bagan J. Potential Non-Invasive Biomarkers for Early Diagnosis of Oral Squamous Cell Carcinoma. J Clin Med. 2021;10(8):1658. Published 2021 Apr 13. doi:10.3390/jcm10081658
22. Ding X, Zhang N, Cai Y, et al. Down-regulation of tumor suppressor MTUS1/ATIP is associated with enhanced proliferation, poor differentiation and poor prognosis in oral tongue squamous cell carcinoma. *Mol Oncol*. 2012;6(1):73-80. doi:10.1016/j.molonc.2011.11.002
23. Ding L, Li B, Zhao Y, et al. Serum CCL2 and CCL3 as potential biomarkers for the diagnosis of oral squamous cell carcinoma. *Tumour Biol*. 2014;35(10):10539-10546. doi:10.1007/s13277-014-2306-1
24. Ding L, Hu EL, Xu YJ, et al. Serum IL-17F combined with VEGF as potential diagnostic biomarkers for oral squamous cell carcinoma. *Tumour Biol*. 2015;36(4):2523-2529. doi:10.1007/s13277-014-2867-z
25. Ding X, Zheng Y, Wang Z, et al. Expression and oncogenic properties of membranous Notch1 in oral leukoplakia and oral squamous cell carcinoma. *Oncol Rep*. 2018;39(6):2584-2594. doi:10.3892/or.2018.6335
26. Ding L, Zhao X, Zhu N, Zhao M, Hu Q, Ni Y. The balance of serum IL-18/IL-37 levels is disrupted during the development of oral squamous cell carcinoma. Surg Oncol. 2020;32:99-107. doi:10.1016/j.suronc.2019.12.001
27. Dong Y, Li Z, Gao M, et al. Immunohistochemical detection of aurora A and ERK pathway in oral leukoplakia and oral squamous cell carcinoma. *Journal of hard tissue biology*. 2014; 23(1), 71-76.
28. Duś-Ilnicka I, Radwan-Oczko M, Gerber H, Hałoń A. Histopathological assessment of oral leukoplakia. Osteonectin as possible biomarker for further diagnostics. Pol J Pathol. 2020;71(2):138-145. doi:10.5114/pjp.2020.97021
29. Dwivedi R, Chandra S, Mehrotra D, Raj V, Pandey R. Predicting transition from oral pre-malignancy to malignancy via Bcl-2 immuno-expression: Evidence and lacunae. J Oral Biol Craniofac Res. 2020;10(4):397-403. doi:10.1016/j.jobcr.2020.07.003
30. Eversole LR, Sapp JP. c-myc oncoprotein expression in oral precancerous and early cancerous lesions. *Eur J Cancer B Oral Oncol*. 1993;29B(2):131-135. doi:10.1016/0964-1955(93)90035-d
31. Fan GK, Chen J, Ping F, Geng Y. Immunohistochemical analysis of P57(kip2), p53 and hsp60 expressions in premalignant and malignant oral tissues. *Oral Oncol*. 2006;42(2):147-153. doi:10.1016/j.oraloncology.2005.06.017
32. Feng CJ, Li HJ, Li JN, Lu YJ, Liao GQ. Expression of Mcm7 and Cdc6 in oral squamous cell carcinoma and precancerous lesions. *Anticancer Res*. 2008;28(6A):3763-3769.
33. Fernández-Valle Á, Rodrigo JP, García-Pedrero JM, et al. Expression of the voltage-gated potassium channel Kv3.4 in oral leucoplakias and oral squamous cell carcinomas. *Histopathology*. 2016;69(1):91-98. doi:10.1111/his.12917
34. Fernández-Valle Á, Rodrigo JP, Rodríguez-Santamarta T, et al. HERG1 potassium channel expression in potentially malignant disorders of the oral mucosa and prognostic relevance in oral squamous cell carcinoma. *Head Neck*. 2016;38(11):1672-1678. doi:10.1002/hed.24493
35. Fillies T, Jogschies M, Kleinheinz J, Brandt B, Joos U, Buerger H. Cytokeratin alteration in oral leukoplakia and oral squamous cell carcinoma. *Oncol Rep*. 2007;18(3):639-643.
36. Foki E, Gangl K, Kranebitter V, et al. Early effects of cigarette smoke extract on human oral keratinocytes and carcinogenesis in head and neck squamous cell carcinoma. Head Neck. 2020;42(9):2348-2354. doi:10.1002/hed.26247
37. Garg R, Shetti AV, Bagewadi AS. Assessment and Comparison of Salivary Survivin Biomarker in Oral Leukoplakia, Oral Lichen Planus, and Oral Cancer: A Comparative Study. *World Journal of Dentistry*. 2017;8(2):73-76
38. Girod SC, Pfeiffer P, Ries J, Pape HD. Proliferative activity and loss of function of tumour suppressor genes as 'biomarkers' in diagnosis and prognosis of benign and preneoplastic oral lesions and oral squamous cell carcinoma. *Br J Oral Maxillofac Surg*. 1998;36(4):252-260. doi:10.1016/s0266-4356(98)90708-2
39. Gonçalves AS, Mosconi C, Jaeger F, et al. Overexpression of immunomodulatory mediators in oral precancerous lesions. *Hum Immunol*. 2017;78(11-12):752-757. doi:10.1016/j.humimm.2017.09.003
40. Guan WQ, Li Q, Ouyang QM. Expression and Significance of Periostin in Tissues and Serum in Oral Leukoplakia and Squamous Cell Carcinoma. *Cancer Biother Radiopharm*. 2019;34(7):444-450. doi:10.1089/cbr.2018.2764
41. Hamidi S, Salo T, Kainulainen T, Epstein J, Lerner K, Larjava H. Expression of alpha(v)beta6 integrin in oral leukoplakia. *Br J Cancer*. 2000;82(8):1433-1440. doi:10.1054/bjoc.1999.1130
42. He Y, Chen Q, Li B. ATM in oral carcinogenesis: association with clinicopathological features. *J Cancer Res Clin Oncol*. 2008;134(9):1013-1020. doi:10.1007/s00432-008-0365-7
43. Herrera Costa F, Narana Ribeiro El Achkar V, Costa V, et al. Different Expression of Aldehyde Dehydrogenases 1A1 and 2 in Oral Leukoplakia With Epithelial Dysplasia and in Oral Squamous Cell Carcinoma. *Appl Immunohistochem Mol Morphol*. 2019;27(7):537-542. doi:10.1097/PAI.0000000000000612
44. Hoffmann RR, Yurgel LS, Campos MM. Evaluation of salivary endothelin-1 levels in oral squamous cell carcinoma and oral leukoplakia. *Regul Pept*. 2011;166(1-3):55-58. doi:10.1016/j.regpep.2010.08.006
45. Hu F, Chen X, Liu X, et al. Clinicopathological features and prognostic implications of Raf kinase inhibitor protein downregulation in tongue squamous cell carcinoma. *Oncol Lett*. 2015;10(3):1303-1308. doi:10.3892/ol.2015.3496
46. Humayun S, Prasad VR. Expression of p53 protein and ki-67 antigen in oral premalignant lesions and oral squamous cell carcinomas: An immunohistochemical study. *Natl J Maxillofac Surg*. 2011;2(1):38-46. doi:10.4103/0975-5950.85852
47. Jing Y, Zhou Q, Zhu H, et al. Ki-67 is an independent prognostic marker for the recurrence and relapse of oral squamous cell carcinoma. Oncol Lett. 2019;17(1):974-980. doi:10.3892/ol.2018.9647
48. Juneja S, Chaitanya NB, Agarwal M. Immunohistochemical expression of Bcl-2 in oral epithelial dysplasia and oral squamous cell carcinoma. *Indian J Cancer*. 2015;52(4):505-510. doi:10.4103/0019-509X.178411
49. Kannan S, Balaram P, Chandran GJ, Pillai MR, Mathew B, Nair MK. Co-expression of ras p21 and epidermal growth factor receptor during various stages of tumour progression in oral mucosa. *Tumour Biol*. 1994;15(2):73-81. doi:10.1159/000217877
50. Khan Z, Tiwari RP, Mulherkar R, et al. Detection of survivin and p53 in human oral cancer: correlation with clinicopathologic findings. *Head Neck*. 2009;31(8):1039-1048. doi:10.1002/hed.21071
51. Kitamura R, Toyoshima T, Tanaka H, et al. Association of cytokeratin 17 expression with differentiation in oral squamous cell carcinoma. *J Cancer Res Clin Oncol*. 2012;138(8):1299-1310. doi:10.1007/s00432-012-1202-6
52. Klein IP, Meurer L, Danilevicz CK, Squarize CH, Martins MD, Carrard VC. BMI-1 expression increases in oral leukoplakias and correlates with cell proliferation. J Appl Oral Sci. 2020;28:e20190532. doi:10.1590/1678-7757-2019-0532
53. Kouketsu A, Sato I, Oikawa M, et al. Expression of immunoregulatory molecules PD-L1 and PD-1 in oral cancer and precancerous lesions: A cohort study of Japanese patients. *J Craniomaxillofac Surg*. 2019;47(1):33-40. doi:10.1016/j.jcms.2017.04.013
54. Lameira AG, Pontes FS, Guimarães DM, et al. MCM3 could be a better marker than Ki-67 for evaluation of dysplastic oral lesions: an immunohistochemical study. *J Oral Pathol Med*. 2014;43(6):427-434. doi:10.1111/jop.12153
55. Lin CY, Chen WH, Liao CT, et al. Positive association of glucose-regulated protein 78 during oral cancer progression and the prognostic value in oral precancerous lesions. *Head Neck*. 2010;32(8):1028-1039. doi:10.1002/hed.21287
56. Lin L, Wang J, Liu D, et al. Interleukin-37 expression and its potential role in oral leukoplakia and oral squamous cell carcinoma. *Sci Rep*. 2016;6:26757. doi:10.1038/srep26757
57. Liu S, Ye D, Wang T, et al. Repression of GPRC5A is associated with activated STAT3, which contributes to tumor progression of head and neck squamous cell carcinoma. *Cancer Cell Int*. 2017;17:34. doi:10.1186/s12935-017-0406-x
58. Logeswari J, Malathi N, Thamizhchelvan H, Sangeetha N, Nirmala SV. Expression of podoplanin in oral premalignant and malignant lesions and its potential as a biomarker. *Indian J Dent Res*. 2014;25(3):305-310. doi:10.4103/0970-9290.138321
59. Lopes NM, Xavier FCA, Ortiz RC, et al. Subcellular localization and expression of E-cadherin and SNAIL are relevant since early stages of oral carcinogenesis. *Pathol Res Pract*. 2018;214(8):1185-1191. doi:10.1016/j.prp.2018.06.004
60. Madan M, Chandra S, Raj V, Madan R. Evaluation of cell proliferation in malignant and potentially malignant oral lesions. *J Oral Maxillofac Pathol*. 2015;19(3):297-305. doi:10.4103/0973-029X.174613
61. Mao T, Xiong H, Hu X, et al. DEC1: a potential biomarker of malignant transformation in oral leukoplakia. Braz Oral Res. 2020;34:e052. doi:10.1590/1807-3107bor-2020.vol34.0052
62. Markopoulos AK, Deligianni E, Antoniades DZ. Heat shock protein 70 membrane expression in oral cancer: a possible new target in antineoplastic therapy?. *Chemotherapy*. 2009;55(4):211-214. doi:10.1159/000218099
63. Matsubara R, Kawano S, Kiyosue T, et al. Increased ΔNp63 expression is predictive of malignant transformation in oral epithelial dysplasia and poor prognosis in oral squamous cell carcinoma. *Int J Oncol*. 2011;39(6):1391-1399. doi:10.3892/ijo.2011.1151
64. Matta A, Bahadur S, Duggal R, Gupta SD, Ralhan R. Over-expression of 14-3-3zeta is an early event in oral cancer. *BMC Cancer*. 2007;7:169. doi:10.1186/1471-2407-7-169
65. Matta A, Tripathi SC, DeSouza LV, et al. Heterogeneous ribonucleoprotein K is a marker of oral leukoplakia and correlates with poor prognosis of squamous cell carcinoma. *Int J Cancer*. 2009;125(6):1398-1406. doi:10.1002/ijc.24517
66. Meng W, Xia Q, Wu L, et al. Downregulation of TGF-beta receptor types II and III in oral squamous cell carcinoma and oral carcinoma-associated fibroblasts. *BMC Cancer*. 2011;11:88. doi:10.1186/1471-2407-11-88
67. Moraes JK, Wagner VP, Fonseca FP, et al. Activation of BDNF/TrkB/Akt pathway is associated with aggressiveness and unfavorable survival in oral squamous cell carcinoma. Oral Dis. 2019;25(8):1925-1936. doi:10.1111/odi.13190
68. Nakabayashi M, Osaki M, Kodani I, et al. PITX1 is a reliable biomarker for predicting prognosis in patients with oral epithelial dysplasia. *Oncol Lett*. 2014;7(3):750-754. doi:10.3892/ol.2013.1775
69. Nanda KD, Ranganathan K, Devi U, Joshua E. Increased expression of CK8 and CK18 in leukoplakia, oral submucous fibrosis, and oral squamous cell carcinoma: an immunohistochemistry study. *Oral Surg Oral Med Oral Pathol Oral Radiol*. 2012;113(2):245-253. doi:10.1016/j.tripleo.2011.05.034
70. Narashiman S, Narasimhan M, Venkatraman G. Expression of Mucin 4 in leukoplakia and oral squamous cell carcinoma: An immunohistochemical study. *J Oral Maxillofac Pathol*. 2014;18(1):25-31. doi:10.4103/0973-029X.131887
71. Nasser W, Flechtenmacher C, Holzinger D, Hofele C, Bosch FX. Aberrant expression of p53, p16INK4a and Ki-67 as basic biomarker for malignant progression of oral leukoplakias. *J Oral Pathol Med*. 2011;40(8):629-635. doi:10.1111/j.1600-0714.2011.01026.x
72. Nayyar AS, Khan M. In search of malignant transformation: a pilot study. *J Cancer Res Ther*. 2012;8(2):277-281. doi:10.4103/0973-1482.98987
73. Negi A, Puri A, Gupta R, Nangia R, Sachdeva A, Mittal M. Comparison of Immunohistochemical Expression of Antiapoptotic Protein Survivin in Normal Oral Mucosa, Oral Leukoplakia, and Oral Squamous Cell Carcinoma. *Patholog Res Int*. 2015;2015:840739. doi:10.1155/2015/840739
74. Nguyen CT, Okamura T, Morita KI, et al. LAMC2 is a predictive marker for the malignant progression of leukoplakia. *J Oral Pathol Med*. 2017;46(3):223-231. doi:10.1111/jop.12485
75. Nogami T, Kuyama K, Yamamoto H. Histopathological and immunohistochemical study of malignant transformation of oral leukoplakia, with special reference to apoptosis-related gene products and proliferative activity. *Acta Otolaryngol*. 2003;123(6):767-775. doi:10.1080/00016480310000700b
76. Ohkura S, Kondoh N, Hada A, et al. Differential expression of the keratin-4, -13, -14, -17 and transglutaminase 3 genes during the development of oral squamous cell carcinoma from leukoplakia. *Oral Oncol*. 2005;41(6):607-613. doi:10.1016/j.oraloncology.2005.01.011
77. Palani J, Lakshminarayanan V, Kannan R. Immunohistochemical detection of human telomerase reverse transcriptase in oral cancer and pre-cancer. *Indian J Dent Res*. 2011;22(2):362. doi:10.4103/0970-9290.84281
78. Pande P, Mathur M, Shukla NK, Ralhan R. pRb and p16 protein alterations in human oral tumorigenesis. *Oral Oncol*. 1998;34(5):396-403. doi:10.1016/s1368-8375(98)00024-4
79. Paneer Selvam N, Sadaksharam J. Salivary interleukin-6 in the detection of oral cancer and precancer. *Asia Pac J Clin Oncol*. 2015;11(3):236-241. doi:10.1111/ajco.12330
80. Patil A, Patil K, Tupsakhare S, Gabhane M, Sonune S, Kandalgaonkar S. Evaluation of Podoplanin in Oral Leukoplakia and Oral Squamous Cell Carcinoma. *Scientifica (Cairo)*. 2015;2015:135298. doi:10.1155/2015/135298
81. Pereira T, Shetty S, Pereira S. Estimation of serum lactate dehydrogenase level in patients with oral premalignant lesions/conditions and oral squamous cell carcinoma: a clinicopathological study. *J Cancer Res Ther*. 2015;11(1):78-82. doi:10.4103/0973-1482.150352
82. Pontes HA, de Aquino Xavier FC, da Silva TS, et al. Metallothionein and p-Akt proteins in oral dysplasia and in oral squamous cell carcinoma: an immunohistochemical study. *J Oral Pathol Med*. 2009;38(8):644-650. doi:10.1111/j.1600-0714.2009.00787.x
83. Pontes HA, Pontes FS, Fonseca FP, et al. Nuclear factor κB and cyclooxygenase-2 immunoexpression in oral dysplasia and oral squamous cell carcinoma. *Ann Diagn Pathol*. 2013;17(1):45-50. doi:10.1016/j.anndiagpath.2012.04.008
84. Poomsawat S, Buajeeb W, Khovidhunkit SO, Punyasingh J. Alteration in the expression of cdk4 and cdk6 proteins in oral cancer and premalignant lesions. *J Oral Pathol Med*. 2010;39(10):793-799. doi:10.1111/j.1600-0714.2010.00909.x
85. Poomsawat S, Punyasingh J, Vejchapipat P. Overexpression of survivin and caspase 3 in oral carcinogenesis. *Appl Immunohistochem Mol Morphol*. 2014;22(1):65-71. doi:10.1097/PAI.0b013e31828a0d0c
86. Priyanka KP, Majumdar S, Kotina S, Uppala D, Balla H. Expression of Heat Shock Protein 70 in Oral Epithelial Dysplasia and Oral Squamous Cell Carcinoma: An Immunohistochemical Study. Contemp Clin Dent. 2019;10(2):185-190. doi:10.4103/ccd.ccd_101_18
87. Ralhan R, Narayan M, Salotra P, Shukla NK, Chauhan SS. Evaluation of P-glycoprotein expression in human oral oncogenesis: correlation with clinicopathological features. *Int J Cancer*. 1997;72(5):728-734. doi:10.1002/(sici)1097-0215(19970904)72:5<728::aid-ijc4>3.0.co;2-u
88. Rathee R, Devi A, Narwal A, Kamboj M, Singh S. Immunohistochemical Coexpression of MUC1 and MUC4 in Oral Leukoplakia and Oral Squamous Cell Carcinoma. Head Neck Pathol. 2021;15(3):831-842. doi:10.1007/s12105-021-01291-y
89. Ravi D, Nalinakumari KR, Rajaram RS, Nair MK, Pillai MR. Expression of programmed cell death regulatory p53 and bcl-2 proteins in oral lesions. *Cancer Lett*. 1996;105(2):139-146. doi:10.1016/0304-3835(96)04258-9
90. Renkonen J, Wolff H, Paavonen T. Expression of cyclo-oxygenase-2 in human tongue carcinoma and its precursor lesions. *Virchows Arch*. 2002;440(6):594-597. doi:10.1007/s00428-002-0616-y
91. Ries J, Agaimy A, Wehrhan F, et al. Importance of the PD-1/PD-L1 Axis for Malignant Transformation and Risk Assessment of Oral Leukoplakia. Biomedicines. 2021;9(2):194. Published 2021 Feb 16. doi:10.3390/biomedicines9020194
92. Routray S, Kheur SM, Kheur M. Osteopontin: a marker for invasive oral squamous cell carcinoma but not for potentially malignant epithelial dysplasias. *Ann Diagn Pathol*. 2013;17(5):421-424. doi:10.1016/j.anndiagpath.2013.03.005
93. Sabitha S, Mohan J, Nirmal RM, et al. Immunoexpressionof p53, Bax and hTERT in Oral Epithelial Dysplasia and Oral Squamous Cell Carcinoma–A Comparative Study. *Journal of Advanced Medical and Dental Sciences Research.* 2018; 6(11), 30.
94. Saddiwal R, Hebbale M, Sane VD, Hiremutt D, Gupta R, Merchant Y. Estimation of Serum Beta 2-Microglobulin Levels in Individuals Exposed to Carcinogens: Clinical Study in Indian Population. *J Maxillofac Oral Surg*. 2017;16(1):53-57. doi:10.1007/s12663-016-0914-6
95. Saileela A, Ravi SB. Expression of Heat Shock Protein 27 in Oral Potentially Malignant Disorders and Oral Squamous Cell Carcinoma: An Immunohistochemical Study. *World Journal of Dentistry*. 2018;9(2):101-105
96. Sakthivel R, Ramamoorthy A, Jeddy N, Singaram M. Evaluation and Expression of Survivin in Potentially Malignant Lesions and Squamous Cell Carcinoma: A Comparative Study. Cureus. 2020;12(4):e7551. doi:10.7759/cureus.7551
97. Santhi WS, Sebastian P, Varghese BT, Prakash O, Pillai MR. NF-kappaB and COX-2 during oral tumorigenesis and in assessment of minimal residual disease in surgical margins. *Exp Mol Pathol*. 2006;81(2):123-130. doi:10.1016/j.yexmp.2006.05.001
98. Sawant S, Dongre H, Ahire C, et al. Alterations in desmosomal adhesion at protein and ultrastructure levels during the sequential progressive grades of human oral tumorigenesis. *Eur J Oral Sci*. 2018;126(4):251-262. doi:10.1111/eos.12426
99. Servato JPS, Ueira Vieira C, de Faria PR, Cardoso SV, Loyola AM. The importance of inducible nitric oxide synthase and nitrotyrosine as prognostic markers for oral squamous cell carcinoma. *J Oral Pathol Med*. 2019;48(10):967-975. doi:10.1111/jop.12942
100. Shah PH, Venkatesh R, More CB. Determination of role of ceruloplasmin in oral potentially malignant disorders and oral malignancy-A cross-sectional study. *Oral Dis*. 2017;23(8):1066-1071. doi:10.1111/odi.12690
101. Shetty SR, Chadha R, Babu S, Kumari S, Bhat S, Achalli S. Salivary lactate dehydrogenase levels in oral leukoplakia and oral squamous cell carcinoma: a biochemical and clinicopathological study. *J Cancer Res Ther*. 2012;8 Suppl 1:S123-S125. doi:10.4103/0973-1482.92226
102. Shyam N, Rao NN, Narang RD, George J, Bommu SR, Kiran G. Immunohistochemical characterization of cyclin dependent kinase-4 in different histological grades of oral leukoplakia and oral squamous cell carcinoma. *J Oral Maxillofac Pathol*. 2014;18(1):36-41. doi:10.4103/0973-029X.131896
103. Singh KN, Ramadas MN, Veeran V, Naidu MR, Dhanaraj TS, Chandrasekaran K. Expression Pattern of the Cancer Stem Cell Marker "Nestin" in Leukoplakia and Oral Squamous Cell Carcinoma. Rambam Maimonides Med J. 2019;10(4):e0024. doi:10.5041/RMMJ.10378
104. Singh DN, Srivastava KC, Potsangbam AD, et al. A Case-control Study Comparing and Correlating iNOS Expression among Various Clinicopathological Variants of Oral Leukoplakia and Oral Squamous Cell Carcinoma: A Immunohistochemistry Study. J Pharm Bioallied Sci. 2020;12(Suppl 1):S324-S331. doi:10.4103/jpbs.JPBS_96_20
105. Singla S, Singla G, Zaheer S, Rawat DS, Mandal AK. Expression of p53, epidermal growth factor receptor, c-erbB2 in oral leukoplakias and oral squamous cell carcinomas. *J Cancer Res Ther*. 2018;14(2):388-393. doi:10.4103/0973-1482.191027
106. Sivadasan P, Gupta MK, Sathe G, et al. Salivary proteins from dysplastic leukoplakia and oral squamous cell carcinoma and their potential for early detection. J Proteomics. 2020;212:103574. doi:10.1016/j.jprot.2019.103574
107. Soares CP, Zuanon JA, Teresa DB, et al. Quantitative cell-cycle protein expression in oral cancer assessed by computer-assisted system. *Histol Histopathol*. 2006;21(7):721-728. doi:10.14670/HH-21.721
108. Soni S, Kaur J, Kumar A, et al. Alterations of rb pathway components are frequent events in patients with oral epithelial dysplasia and predict clinical outcome in patients with squamous cell carcinoma. *Oncology*. 2005;68(4-6):314-325. doi:10.1159/000086970
109. Sravya T, Sivaranjani Y, Rao GV. Immunohistochemical expression of budding uninhibited by benzimidazole related 1 in leukoplakia and oral squamous cell carcinoma. *J Oral Maxillofac Pathol*. 2016;20(1):71-77. doi:10.4103/0973-029X.180938
110. Srinivasan M, Jewell SD. Evaluation of TGF-alpha and EGFR expression in oral leukoplakia and oral submucous fibrosis by quantitative immunohistochemistry. *Oncology*. 2001;61(4):284-292. doi:10.1159/000055335
111. Sudha VM, Hemavathy S. Role of bcl-2 oncoprotein in oral potentially malignant disorders and squamous cell carcinoma: an immunohistochemical study. *Indian J Dent Res*. 2011;22(4):520-525. doi:10.4103/0970-9290.90286
112. Sun Y, Liu N, Guan X, Wu H, Sun Z, Zeng H. Immunosuppression Induced by Chronic Inflammation and the Progression to Oral Squamous Cell Carcinoma. *Mediators Inflamm*. 2016;2016:5715719. doi:10.1155/2016/5715719
113. Sundar S, Ramani P, Sherlin HJ, Jayaraj G. Expression of “periostin” in leukoplakia and oral submucous fibrosis: An immunohistochemical study. *Journal of International Oral Health*. 2021;13(3), 267.
114. Sutariya RV, Manjunatha BS. Immunohistochemical study of p21 and Bcl-2 in leukoplakia, oral submucous fibrosis and oral squamous cell carcinoma. *J Exp Ther Oncol*. 2016;11(4):285-292.
115. Tashiro K, Oikawa M, Miki Y, Takahashi T, Kumamoto H. Immunohistochemical assessment of growth factor signaling molecules: MAPK, Akt, and STAT3 pathways in oral epithelial precursor lesions and squamous cell carcinoma. *Odontology*. 2020;108(1):91-101. doi:10.1007/s10266-019-00428-4
116. Thiem DGE, Schneider S, Venkatraman NT, et al. Semiquantifiable angiogenesis parameters in association with the malignant transformation of oral leukoplakia. *J Oral Pathol Med*. 2017;46(9):710-716. doi:10.1111/jop.12544
117. Thorup AK, Reibel J, Schiødt M, et al. Can alterations in integrin and laminin-5 expression be used as markers of malignancy?. *APMIS*. 1998;106(12):1170-1180. doi:10.1111/j.1699-0463.1998.tb00274.x
118. Tripathi SC, Matta A, Kaur J, et al. Nuclear S100A7 is associated with poor prognosis in head and neck cancer. *PLoS One*. 2010;5(8):e11939. doi:10.1371/journal.pone.0011939
119. Turatti E, da Costa Neves A, de Magalhães MH, de Sousa SO. Assessment of c-Jun, c-Fos and cyclin D1 in premalignant and malignant oral lesions. *J Oral Sci*. 2005;47(2):71-76. doi:10.2334/josnusd.47.71
120. Vadla P, Yeluri S, Deepthi G, Guttikonda VR, Taneeru S, Naramala S. Stathmin! An immunohistochemical analysis of the novel marker in Oral Squamous Cell Carcinoma and Oral Leukoplakia. Asian Pac J Cancer Prev. 2020;21(11):3317-3323. Published 2020 Nov 1. doi:10.31557/APJCP.2020.21.11.3317
121. Varghese SS, Sunil PM, Madhavan RN. Expression of inducible nitric oxide synthase (iNOS) in oral precancer and oral squamous cell carcinoma: an immunohistochemical study. *Cancer Biomark*. 2010;8(3):155-160. doi:10.3233/CBM-2011-0207
122. Varun BR, Ranganathan K, Rao UK, Joshua E. Immunohistochemical detection of p53 and p63 in oral squamous cell carcinoma, oral leukoplakia, and oral submucous fibrosis. *J Investig Clin Dent*. 2014;5(3):214-219. doi:10.1111/jicd.12038
123. Vigneswaran N, Baucum DC, Wu J, et al. Repression of tumor necrosis factor-related apoptosis-inducing ligand (TRAIL) but not its receptors during oral cancer progression. *BMC Cancer*. 2007;7:108. doi:10.1186/1471-2407-7-108
124. Vijayakumar G, Narwal A, Kamboj M, Sen R. Association of SOX2, OCT4 and WNT5A Expression in Oral Epithelial Dysplasia and Oral Squamous Cell Carcinoma: An Immunohistochemical Study. Head Neck Pathol. 2020;14(3):749-757. doi:10.1007/s12105-019-01114-1
125. von Zeidler SV, de Souza Botelho T, Mendonça EF, Batista AC. E-cadherin as a potential biomarker of malignant transformation in oral leukoplakia: a retrospective cohort study. *BMC Cancer*. 2014;14:972. doi:10.1186/1471-2407-14-972
126. Vora HH, Trivedi TI, Shukla SN, Shah NG, Goswami JV, Shah PM. p53 expression in leukoplakia and carcinoma of the tongue. *Int J Biol Markers*. 2006;21(2):74-80. doi:10.5301/jbm.2008.5628
127. Wagner VP, Cardoso PR, Dos Santos JN, et al. Immunohistochemical Study of TGF-β1 in Oral Leukoplakia and Oral Squamous Cell Carcinoma: Correlations Between Clinicopathologic Factors and Overall Survival. *Appl Immunohistochem Mol Morphol*. 2017;25(9):651-659. doi:10.1097/PAI.0000000000000355
128. Wang Z, Feng X, Liu X, et al. Involvement of potential pathways in malignant transformation from oral leukoplakia to oral squamous cell carcinoma revealed by proteomic analysis. *BMC Genomics*. 2009;10:383. doi:10.1186/1471-2164-10-383
129. Wang X, Jiang W, Duan N, et al. NOD1, RIP2 and Caspase12 are potentially novel biomarkers for oral squamous cell carcinoma development and progression. *Int J Clin Exp Pathol*. 2014;7(4):1677-1686.
130. Wang X, Jin Y, Li YX, Yang Y. Secretory leukocyte peptidase inhibitor expression and apoptosis effect in oral leukoplakia and oral squamous cell carcinoma. *Oncol Rep*. 2018;39(4):1793-1804. doi:10.3892/or.2018.6251
131. Wang S, Li F, Fan H, Xu J, Hu Z. Expression of PIWIL2 in oral cancer and leukoplakia: Prognostic implications and insights from tumors. Cancer Biomark. 2019;26(1):11-20. doi:10.3233/CBM-182009
132. Wilkman TS, Hietanen JH, Malmström MJ, Konttinen YT. Immunohistochemical analysis of the oncoprotein c-erbB-2 expression in oral benign and malignant lesions. *Int J Oral Maxillofac Surg*. 1998;27(3):209-212. doi:10.1016/s0901-5027(98)80012-x
133. Winter J, Pantelis A, Reich R, et al. Risk estimation for a malignant transformation of oral lesions by S100A7 and Doc-1 gene expression. *Cancer Invest*. 2011;29(7):478-484. doi:10.3109/07357907.2011.597813
134. Xia J, Wang J, Chen N, et al. Expressions of CXCR7/ligands may be involved in oral carcinogenesis. *J Mol Histol*. 2011;42(2):175-180. doi:10.1007/s10735-011-9322-x
135. Xia J, Chen N, Hong Y, et al. Expressions of CXCL12/CXCR4 in oral premalignant and malignant lesions. *Mediators Inflamm*. 2012;2012:516395. doi:10.1155/2012/516395
136. Xia RH, Song XM, Wang XJ, Li J, Mao L. The combination of SMAD4 expression and histological grade of dysplasia is a better predictor for the malignant transformation of oral leukoplakia. *PLoS One*. 2013;8(6):e66794. doi:10.1371/journal.pone.0066794
137. Xu XC, Lee JS, Lippman SM, Ro JY, Hong WK, Lotan R. Increased expression of cytokeratins CK8 and CK19 is associated with head and neck carcinogenesis. *Cancer Epidemiol Biomarkers Prev*. 1995;4(8):871-876.
138. Yamada T, Ohno S, Kitamura N, Sasabe E, Yamamoto T. SPARC is associated with carcinogenesis of oral squamous epithelium and consistent with cell competition. *Med Mol Morphol*. 2015;48(3):129-137. doi:10.1007/s00795-014-0089-5
139. Ye X, Wang X, Lu R, Zhang J, Chen X, Zhou G. CD47 as a potential prognostic marker for oral leukoplakia and oral squamous cell carcinoma. *Oncol Lett*. 2018;15(6):9075-9080. doi:10.3892/ol.2018.8520
140. Yoshida K, Sato K, Tonogi M, Tanaka Y, Yamane GY, Katakura A. Expression of Cytokeratin 14 and 19 in Process of Oral Carcinogenesis. *Bull Tokyo Dent Coll*. 2015;56(2):105-111. doi:10.2209/tdcpublication.56.105
141. Zhang X, Kim KY, Zheng Z, Bazarsad S, Kim J. Nomogram for risk prediction of malignant transformation in oral leukoplakia patients using combined biomarkers. *Oral Oncol*. 2017;72:132-139. doi:10.1016/j.oraloncology.2017.07.015
142. Zhu M, Liu W, Shi L, et al. Expression of DNA doublestrand repair proteins in oral leukoplakia and the risk of malignant transformation. *Oncol Lett*. 2018;15(6):9827-9835. doi:10.3892/ol.2018.8574

# Appendix 4. Qualitative synthesis of protein expression profile among OSCC, OL, and control groups.

| Protein  (Gene) | Number of studies (Refs) | Qualitative analysis – Protein expression pattern |
| --- | --- | --- |
| ATP-dependent translocase ABCB1 (*ABCB1)* | 1  (67) | Expression of P-gp proportionately increased according to severity of dysplasia. Also, markedly increased levels in primary OSCC and significantly higher levels in recurrent OSCCs were observed, indicating that alteration in P-gp expression may be an early event in oral oncogenesis. |
| Atypical chemokine receptor 3 (*ACKR3)* | 1  (68) | Significantly upregulated in OL (p=0.005) and OSCC (p=0.006) tissue samples compared to normal epithelia. |
| RAC-alpha serine/threonine-protein kinase (*AKT1)* | 3  (69–71) | Normal mucosa and OL showed similar expression of p‐Akt. OSCC presented increased expression of p‐Akt compared to normal mucosa and OL / There was a significant increase in p-Akt positivity comparing normal oral mucosa to severe dysplasia and OSCC (p<0.01) / pAkt expression was significantly enhanced in OSCC compared to non-dysplastic OL (p<0.01). |
| Albumin (*ALB)* | 1  (72) | There is still a need for further studies assessing sera levels of albumin to confirm its utility and to assess its role in the pathogenesis and their impact on the prognosis of OSCC. |
| Retinal dehydrogenase 1 (*ALDH1A1)* | 1  (73) | ALDH1A1 positive expression occurred in the basal and suprabasal layers in 100% and 83.3% of OL cases, respectively. ALDH1A1 expression was negative in 70% of the OSCC cases, mostly in moderately differentiated tumors. |
| Aldehyde dehydrogenase, mitochondrial (*ALDH2)* | 1  (73) | ALDH2 positive expression occurred in the basal and suprabasal layers in 48.1% of the cases. In the OSCC cases, ALDH2 expression was positive in all cases. |
| Serine-protein kinase ATM (*ATM)* | 2  (74,75) | ATM expression gradually increased in normal tissue, OL and OSCC groups during carcinogenesis (p=0.005). Significant difference on high expression rate of ATM in untransformed compared to transformed OL was observed (p=0.037) / 2). Significant diference (P = 0.030) was found only between OL and normal controls |
| Aurora kinase A (*AURKA)* | 1  (76) | OSCC patients tended to have significantly higher Aurora A expression than OL patients (p=0.008). Suggesting that this protein might be indicator of malignant transformation of OL. |
| Beta-2-microglobulin (*B2M)* | 1  (77) | Progressively increasing serum levels positively correlated with the degree of cellular atypia from mild dysplasia to moderately differentiated OSCC (p<0.001) |
| Apoptosis regulator BAX (*BAX)* | 2  (37,63) | 1) Control cells were weakly positive for Bax, OL were moderate to strong, and OSCC were again weak / 2) There was a statistically significant expression of BAX between normal mucosa and OL and OSCC (*p*<0.05). |
| Apoptosis regulator Bcl-2 (*BCL2)* | 7  (37,78–83) | Bcl-2 expression among healthy tissues (23.94±19.16), non-dysplastic OL (28.40±25.87), dysplastic OL (31.21±25.47), and OSCC (24.63±22.68) was statistically different (p=0.000) / The mean LI in normal tissue was 14.16 ± 6.43, 15.30 ± 9.94 in non-dysplastic OL, 33.78 ± 14.29 in dysplastic OL and 15.60 ± 18.47 in OSCC/ Bcl-2 positivity was seen in 26.7% of OL cases and among 30.0% of OSCC cases with no statistically significant difference (p > 0.05) / All normal cases were Bcl-2 negative, a few positive cells were detected on OL and many positive cells were seen in OSCC / All OSCC and dysplastic OL tissues expressed Bcl-2, while none hyperplastic OL had more than 3% Bcl-2 positive cells / Bcl-2 positivity was observed in all cases (100%) of OL and in 83.33-86.7% of OSCC cases with no statistical significance. |
| Brain-derived neurotrophic factor (*BDNF)* | 1  (69) | Normal mucosa and OL showed similar expression of BDNF. OSCC presented increased expression of BNDFcompared to normal mucosa and OL. |
| Class E basic helix-loop-helix protein 40 - DEC1 (*BHLHE40)* | 1  (84) | Increased expression from normal mucosa to OL to OSCC (p < 0.0001). |
| Baculoviral IAP repeat-containing protein 5 – Survivin (*BIRC5)* | 5  (39,41–43,85,86) | The expression of survivin was higher in OSCC (80%) when compared to OL (70%), and normal mucosa (35%) (p = 0.015). 72% of OSCC revealed survivin expression, which was significantly higher than normal oral tissues (0%) (p< 0.0008), while 44% OL were survivin positive. 20% of normal mucosa samples were survivin positive, while 53.33 of OL and 80% of OSCC cases were positive for survivin staining (𝑝<0.001). Survivin expression was significantly higher in the OSCC group than in the normal mucosa (p<0.0001), non-dysplastic and dysplastic OL (p<0.01). The expression of survivin was positive in 70% of OSCC and in 50% of OL samples. All samples of normal oral mucosa were negative for the expression of survivin. |
| Polycomb complex protein BMI-1 (*BMI1)* | 2  (38,69) | BMI-1 expression increases in early oral carcinogenesis, and it may be associated with the occurrence of dysplastic changes |
| Serine/threonine-protein kinase B-raf (*BRAF)* | 1  (71) | Moderate to strong cytoplasmic BRAF staining was observed in basal cell and prickle cell layers of OL and in most OSCC cells |
| Mitotic checkpoint serine/threonine-protein kinase BUB1 beta (*BUB1B)* | 1  (87) | Significant increase on BUBR1 IHC staining intensity scores according to histological grades of dysplasia and OSCC (p=0.00001), suggesting its role as a prognostic indicator. |
| Carbonic anhydrase 9 (*CA9)* | 1  (88) | Analyzing each biomarker in terms of malignant progression, all assessed markers showed statistical significance. |
| Inactive caspase-12 (*CASP12)* | 1  (89) | Moderate/strong staining intensity observed in the normal and OL tissues, while the expression decreased gradually with the progression of OSCC (p<0.001). |
| Caspase-3 (*CASP3)* | 1  (41) | The elevated expression of nuclear caspase 3 in OSCC were demonstrated. These results suggest that Caspase 3 play a crucial role in oral carcinogenesis. The cytoplasmic plus nuclear staining within the same cells of caspase 3 is common in OSCC, implying that this specific expression pattern may be a useful tool for treatment plan of premalignant lesion. |
| C-C motif chemokine 14 (*CCL14)* | 1  (51) | Patients with OL and PL have significantly higher salivary HCC-1 than healthy controls (p<0.01). Notable elevation of HCC-1 levels in OSCC collated to OL sample cohorts (p<0.0001). The levels of HCC-1 considerably increased in OSCC compared to control saliva (p<0.01). |
| C-C motif chemokine 2 (*CCL2)* | 3  (51,90,91) | No significant differences in the number of MCP-1+ cells between OL and OSCC / Patients with OL, PL, and OSCC presented significantly higher salivary MCP-1 than healthy controls (p<0.01). / CCL2 concentration was significantly lower in OSCC patients (67.81 pg/ml) than in OL (86.15 pg/ml, p<0.001) and healthy subjects (108.1 pg/ml p<0.0001). |
| C-C motif chemokine 3 (*CCL3)* | 1  (90) | CCL3 serum level was significantly lower in healthy controls (118.3 pg/ml) than in the OL group (201.9 pg/ml, *p*<0.001) but not significantly different from OSCC group (153.9 pg/ml). |
| Cyclin-D1-binding protein 1 (*CCNDBP1)* | 3  (29,34,92) | Overexpression of Cyclin D1 linearly increased from nondysplastic to dysplastic OL to OSCC and it was significantly higher in OSCC than in OL and normal tissues (p=0.013). Transition of OL to OSCC was associated with overexpression of cyclin D1 (OR = 2.938, p=0.001) |
| Programmed cell death 1 ligand 1 - PD-L1  (*CD274)* | 4  (44,45,53,93) | PD-L1 density was significantly stronger in both OSCC, and OL compared with normal control (p<0.0001). PD-L1 expression tended to increase in association with malignant degrees of oral epithelial lesions, demonstrating significant differences between OL and OSCC (p < 0.001). In transformed OL a significant 60-fold overexpression (p = 0.04) and in OSCC a 99.4-fold increase of PD-L1 was detected. Overexpression was related to malignant transformation (p = 0.03) and malignancy (p = 0.003) |
| T-cell surface glycoprotein CD4 (*CD4)* | 1  (91) | CD4 expression increased gradually according to oral dysplasia, with statistically significant difference from OL to OSCC (p=0.0017). |
| Leukocyte surface antigen CD47 (*CD47)* | 1  (94) | Mean expression in normal mucosa (1.56±0.93) significantly lower compared to dysplastic OL (5.67±2.50; p<0.0001) and OSCC (7.10±3.76; p=0.0057). |
| Macrosialin (*CD68)* | 1  (91) | CD68 expression gradually increased according to oral dysplasia, with statistically significant difference from OL to OSCC (p<0.0001). |
| Cell division control protein 6 homolog (*CDC6)* | 1  (95) | Cdc6 IHC expression: Normal: 0/10, OL: 16/34 and OSCC: 33/54 (LI: 31.2%). High expression of Cdc6 was correlated with OSCC development. Expression and LI values may help distinguish normal tissues from OL and OSCC. Cdc6 expression may potentially become molecular markers for the early diagnosis and prognosis prediction of OSCC. |
| Cadherin-1  (*CDH1)* | 3  (96–98) | Differences in E-cadherin expression were seen between normal mucosa and dysplasia (p ≤ 0.002), normal mucosa and OSCC (p ≤ 0.004) and mild dysplasia and OSCC (p ≤ 0.018). A significant increase in cytoplasmic E-cadherin expression was observed in OSCC compared to normal mucosa (p<0.0001), OL (p<0.01) and dysplastic OL (p< 0.0001). Decreased E-cadherin expression was observed in OSCC compared to dysplastic OL (p=0.0001), in dysplastic OL compared to non-dysplastic OL (p=0.019), and in non-dysplastic OL compared to normal mucosa (p = 0.006). |
| Cyclin-dependent kinase 2-associated protein 1 (*CDK2AP1)* | 1  (99) | Compared to normal tissues, the expression of Doc-1 was decreased in OL and OSCC (p<0.001) and correlated with the malignancy of these lesions. |
| Cyclin-dependent kinase 4 (*CDK4)* | 2  (100,101) | The number of cdk4-positive cases was significantly lower in normal mucosa than in dysplastic OL (p=0.0008) and OSCC (p=0.0213) / The CDK4 immunoreactivity and the staining intensity progressively increased from normal to OL and OSCC (p=0.001). |
| Cyclin-dependent kinase 6 (*CDK6)* | 1  (100) | Highest cdk6 expression observed in OSCC (60%), followed by non-dysplastic OL (25%), dysplastic OL (21.43%) and normal mucosa (8.33%) (OSCC vs normal; p= 0.0140). |
| Cyclin-dependent kinase inhibitor 1 - p21  (*CDKN1A)* | 3  (83,102,103) | 68.6% OSCCs and 60% OL were p21 positive while normal oral tissues did not show p21 immunoreactivity. Non-keratinizing mucosa was p21-negative, 67% of non-dysplastic OL, 80% of dysplastic OL and 69% of OSCC showed p21 positive staining / 3) p21 positivity was seen in 80% of cases of OL and in 80% of OSCC cases, with no statistical significance between the groups. |
| Cyclin-dependent kinase inhibitor 1B - p27 (*CDKN1B)* | 1  (35) | p27 expression was lower in OSCC compared with Mucocele, Moderate Dysplasia and Severe Dysplasia. |
| Cyclin-dependent kinase inhibitor 1C - p57kip2 (*CDKN1C)* | 1  (27) | Progressively decreased expression from control to OL and OSCC (p< 0.05). |
| Cyclin-dependent kinase inhibitor 2A - p16^INK4a^  (*CDKN2A)* | 5  (29,33,34,88,104) | No statistically significant difference was observed in p16 expression among OSCC, OL with and without dysplasia, and normal mucosa (p=0.08). Loss of expression of p16^INK4a^ linearly increased from nondysplastic to dysplastic OL and to OSCC. 37% of OSCC, 41% of OL and 93% of normal oral mucosa exhibited p16 positive immunostaining, with a strong correlation between the expression of p16 in OSCCs (p=0.007) and in OL (p=0.0052). p16 positivity was detected in 45% normal tissues, 41% of OL and in 40% of OSCCs. Significant difference comparing the expression between normal mucosa and OL. In terms of malignant progression, p16 showed statistical significance. |
| Serine/threonine-protein kinase Chk2 (*CHEK2)* | 1  (75) | There was no difference in CHEK2 expression among the groups (p=0.074) |
| Ceruloplasmin  (*CP)* | 1  (105) | The serum levels of ceruloplasmin are associated with progression of the carcinogenesis process, thus, it could be a used as prediction biomarker of malignant transformation of oral precancer. |
| C-reactive protein  (*CRP)* | 1  (106) | CRP was significantly elevated in OL and was further increased in OSCC compared to controls. CRP was highly correlated with disease progression (p<0.001). Logistic regression revealed CRP as one of the best markers for distinguishing OL and OSCC patients from healthy individuals. |
| Macrophage colony-stimulating factor 1 - M-CSF (*CSF1)* | 1  (106) | No differences on serum levels among control, OL, and OSCC groups. |
| Catenin beta-1 (*CTNNB1)* | 1  (88) | Analyzing each biomarker in terms of malignant progression, all assessed markers showed statistical significance. |
| C-X-C motif chemokine 10 (*CXCL10)* | 1  (51) | No appreciable differences were assessed in IP-10 levels among the collated groups. |
| Stromal cell-derived factor 1 (*CXCL12)* | 1  (107) | Expression observed in 50% of OL and in 62.5% OSCC cases, with significant difference from normal epithelia (7.7%) (p=0.01 and p=0.001, respectively). |
| Interleukin-8  (*CXCL8)* | 2  (50,51) | IL-8 was present in all layers of the epithelium in most OSCCs, was observed in few OL samples, and was not present in normal specimens, demonstrating the possibility as biomarker of malignant transformation. Patients with OL, PL and OSCC have significantly higher salivary IL-8 than healthy controls (p<0.01). Notable elevations of IL-8 levels in OSCC collated to OL sample cohorts (p<0.0001). |
| C-X-C chemokine receptor type 4 (*CXCR4)* | 1  (107) | CXCR4 was expressed in 15.4% of normal tissues, in 37.5% of OL and in 60% of OSCC samples, with significant difference between normal tissue and OSCC (p=0.005). CXCL12/CXCR4 may play an important role in oral premalignant stages, contributing to the oral carcinogenesis progression |
| Beta-defensin 1 - hBD-1 (*DEFB1)* | 1  (89) | In the normal and OL tissues, moderate/strong staining intensity was observed, while no or weak staining was noted in OSCC. The expression of hBD1 decreased gradually with the progression of OSCC. hBD1 expression correlated significantly to diagnostic category (p <0.001). |
| Beta-defensin 103 - hBD-3 (*DEFB103A)* | 1  (89) | In the normal and OL tissues, moderate/strong staining intensity was observed, while no or weak staining was noted in OSCC. The expression of hBD3 decreased gradually with the progression of OSCC. hBD3 expression correlated significantly to diagnostic category (p <0.001). |
| Beta-defensin 4A - hBD-2 (*DEFB4A)* | 1  (89) | In the normal and OL tissues, moderate/strong staining intensity was observed, while no or weak staining was noted in OSCC. The expression of hBD2 decreased gradually with the progression of OSCC. hBD2 expression correlated significantly to diagnostic category (p <0.001). |
| Desmoglein-2  (*DSG2)* | 1  (108) | The staining intensity of desmoplakin, plakoglobin, and desmoglein 2 were high in hyperplastic epithelium, and progressively decreased in the subsequent stages of the disease, with significant difference from normal oral epithelium (p<0.05). |
| Desmoplakin  (*DSP)* | 1  (108) | High staining intensity in hyperplastic epithelium that progressively decreased in the subsequent stages of the disease, with significant difference from normal oral epithelium (p<0.05). |
| Endothelin-1  (*EDN1)* | 2  (109,110) | Salivary Endothelin-1 level was 82.78 ± 5.9 pg/ml in OSCC, 57.76 ± 4.1 pg/ml in OL and 29.72 ± 14.1 pg/mL in healthy controls (p < 0.001) / Although ET-1 might display an important role in OSCC, its salivary levels do not seem to be a good biomarker of OSCC grade or malignant transformation. |
| Epidermal growth factor receptor (*EGFR)* | 3  (30,103,111) | Normal mucosa showed moderate EGFR staining, 77% of non-dysplastic OL, 70% of dysplastic OL and 88% of OSCC showed positive EGFR staining. The expression of EGFR increased with progression toward malignancy from control to OL, to OSCC (p ≤ 0.001). The area and intensity of EGFR staining increased significantly in OL and OSCC compared to normal mucosa (p< 0.05). |
| Receptor tyrosine-protein kinase erbB-2 (*ERBB2)* | 2  (30,112) | C‑erbB2 was found to be negative in all specimens. The lower the degree of epithelial differentiation, the higher the expression of c-erbB-2 oncoprotein. |
| Proto-oncogene c-Fos (*FOS)* | 1  (92) | The expression of c-Fos in mild dysplasia was significantly lower than in moderate to severe dysplasia (*p*=0.018), normal mucosa and OSCC (*p=*0.001). |
| Forkhead box protein P3 (*FOXP3)* | 1  (91) | Expression increased gradually according to oral dysplasia, with statistically significant difference from OL to OSCC (p=0.012). |
| Retinoic acid-induced protein 3 (*GPRC5A)* | 1  (113) | GPRC5A expression was gradually suppressed during oncogenesis of HNSCC (p < 0.01). Suppression of GPRC5A may serve as a molecular biomarker for the oncogenesis of HNSCC. |
| Histone H2AX  (*H2AX)* | 1  (75) | γH2AFX expression in OL tissue was associated with OSCC progression. Detection of γH2AFX may be a promising method for an early identification and risk evaluation of OSCC in patients with OL. |
| HLA class I histocompatibility antigen, alpha chain E (*HLA-E)* | 1  (53) | OL presented a significantly higher tissue expression of HLA-E than the control group (p<0.05). The expression of these proteins in OL was similar than the OSCC group (p>0.05) |
| HLA class I histocompatibility antigen alpha chain G (*HLA-G)* | 1  (53) | OL presented a significantly higher expression than control group (p<0.05) but similar expression than OSCC group (p>0.05). There was no significant difference between salivary levels in OL patients and those of the control group (p>0.05). |
| Heterogeneous nuclear ribonucleoprotein K (*HNRNPK)* | 1  (114) | Significant increase in nuclear hnRNP K staining was observed in different stages of tumorigenesis (normal, OL and OSCC) (p< 0.001). nuclear-cytoplasmic translocation may have an important role in malignant transformation of oral cancer. |
| GTPase Hras (*HRAS)* | 1 (71) | HRAS immunoreactivity was detected in the cytoplasm of epithelial cells of OLs and OSCC, with no statistical difference among the groups. |
| Endoplasmic reticulum chaperone BiP (*HSPA5)* | 1  (115) | The expression of GRP78 increased according to the malignant potential of oral lesions, with 14% in OL and 74% in patients with OSCC (p<.0001). Patients with GRP78 hyperexpression had a 4.9-fold increased risk of developing malignancy at the same location (p<0.01) |
| Heat shock protein beta-1 (*HSPB1)* | 1  (116) | Percentage and intensity of positively stained cells significantly higher in OSCC than in normal mucosa (p=0.002), but no significant difference between dysplasia and normal tissues (p=0.504). |
| Hsp70-binding protein 1 (*HSPBP1)* | 2  (117,118) | HSP70 expression was found to increase significantly from normal oral mucosa to dysplastic OL, to OSCC (p= 0.000) / HSP70 expression was significantly higher in OL and OSCC groups than in the control group. Significant increased expression of HSP70 was observed from control to OL and OSCC cases, supporting the critical role of HSP70 in the development of OSCC |
| 60 kDa heat shock protein, mitochondrial (*HSPD1)* | 1  (27) | Expression progressively increased from normal mucosa to OL to OSCC (p< 0.05). |
| Telomerase reverse transcriptase (*hTERT)* | 2  (63,119) | The mean LI increased from normal (28.3±12.3), to OL (44.06±14.6), to OSCC (47.56±21.30) (p=0.00) / Significant differences among normal epithelium, OL and OSCC in hTERT expression (p<0.005). |
| Intercellular adhesion molecule 1 (*ICAM1)* | 1  (106) | ICAM-1 serum level (ng/ml) progressively increased from control (246.4±117.0) to OL (266.9±119.0), to OSCC (317.0±169.6). OSCC vs Control (p<0.05) |
| Interferon gamma (*IFNG)* | 1  (91) | Significantly fewer IFN-𝛾+ cells were seen in OSCC than in OL (p= 0.0001). |
| Interleukin-10 (*IL10)* | 2  (53,91) | OL presented a significantly higher tissue expression of IL-10 than the control group (p<0.05) but similar expression than the OSCC group (p>0.05). / IL-10 expression increased gradually according to oral dysplasia, with statistically significant difference from OL to OSCC (p<0.001). |
| Interleukin-17F (*IL17F)* | 1  (120) | The mean±SEM level (pg/ml) of IL-17F was gradually downregulated from healthy (394.3 ±96.42), OL (169.6±50.58), to OSCC individuals (82.96±11.19) (p<0.05). |
| Interleukin-18 (*IL18)* | 1  (56) | The ratio IL-18/IL-37 was higher in OSCC patients (2.643) than in OL (1.743, p < 0.0001) and controls (1.198, p < 0.0001). The ratio of IL-18/IL-37 may be used as a biomarker for OSCC and risk assessment of malignant transformation of OL. |
| Interleukin-1 alpha (*IL1A)* | 2  (50,51) | No appreciable differences were assessed in IL-1a levels among the collated groups / Concentrations of IL-1a was only higher in saliva of OSCC patients than in controls (p = 0.017). |
| Interleukin-1 beta (*IL1B)* | 1  (121) | IL-1β was significantly higher in saliva of OSCC patients than in OL and control groups (p≤0.05). IL-1β concentrations were not detected in serum |
| Interleukin-37 (*IL37)* | 2  (55,56) | OL and OSCC lesions show higher IL-37 expression than normal tissue (p<0.001), indicating IL-37 as a possible diagnostic and prognostic marker of malignant transformation / IL-37 serum concentration was decreased in OSCC patients (75.02) than in OL (83.51, p=0.033) and controls (95.52, p < 0.0001). |
| Interleukin-4 (*IL4)* | 1 (91) | Significantly fewer IL-4+ cells were seen in OSCC than in OL (p= 0.0001). |
| Interleukin-6  (*IL6)* | 5  (50,51,106,121,122) | There were significant (p<0.01) differences in the serum and saliva IL-6 levels among the groups with significant differences in the saliva levels between PL and OSCC groups (p<0.01). Other study found that salivary IL-6 was significantly higher in OSCC than in OL and control groups (p≤0.05), but there was not difference in IL-6 serum level. Patients with OL and PL have significantly higher salivary IL-6 than healthy controls (p<0.01). Notable elevations of IL-6 levels in OSCC collated to OL sample cohorts (p<0.0001). The mean salivary IL-6 concentration among control (9.68 ± 12.838), OL (43.00 ± 52.143) and OSCC (132.88 ± 59.098) groups was statistically significant different (p<0.001). |
| U3 small nucleolar ribonucleoprotein protein IMP3 (*IMP3)* | 1  (88) | Analyzing each biomarker in terms of malignant progression, all assessed markers showed statistical significance. |
| Integrin alpha-2 (α2𝛽1 integrin) (*ITGA2)* | 2  (123,124) | Compared to healthy mucosa, ITGA-2 was significantly overexpressed in OL (p = 0.002) as well as in OSCC (p = 0.003) / There was no unequivocal expression of the adhesion molecules distinguishing between inflammatory tissue, OL, and OSCC. |
| Integrin alpha-3 (α3𝛽l integrin) (*ITGA3)* | 1  (124) | No unequivocal expression distinguishing between inflammatory tissue, OL, and OSCC. |
| Integrin alpha-6 (α6𝛽4 integrin) (*ITGA6)* | 1  (124) | No unequivocal expression distinguishing between inflammatory tissue, OL, and OSCC. |
| Integrin beta-6 (avb6 integrin) (*ITGB6)* | 1  (125) | Expression necessary but not sufficient for malignant transformation |
| Involucrin (*IVL)* | 1 (126) | Involucrin was detected in all analyzed specimens, but the staining intensity was variable among samples. |
| Transcription factor AP-1 (*JUN)* | 2  (88,92) | The number of c-Jun positive cells was significantly higher in mild dysplasia (p=0.031), moderate to severe dysplasia and OSCC(p<0.001), compared to normal mucosa / Analyzing each biomarker in terms of malignant progression, all assessed markers showed statistical significance. |
| Junction plakoglobin (*JUP)* | 1  (108) | High staining intensity in hyperplastic epithelium that progressively decreased in the subsequent stages of the disease, with significant difference from normal oral epithelium (p<0.05). |
| Potassium voltage-gated channel subfamily C member 4 (*KCNC4)* | 1  (127) | Patients with dysplastic oral epithelial lesions presented an increased risk of malignant progression to OSCC compared to those with hyperplastic lesions (p = 0.009). |
| Potassium voltage-gated channel subfamily H member 2 (*KCNH2)* | 1  (128) | There was no association between HERG1 expression and the risk of progression from oral leukoplakia to OSCC. |
| GTPase Kras (*KRAS)* | 1  (71) | KRAS expression was significantly decreased in OSCC compared to non-dysplastic OL, mild and severe dysplasia (p<0.001). |
| Keratin, type II cytoskeletal 1 (*KRT1)* | 2  (126,129) | Ck 1 expression was significantly reduced in oral squamous cell carcinomas compared to oral leukoplakia (p<0.01). No significant alteration of Ck 1 expression was found within the group of oral leukoplakia dependent on the degrees of histological dysplasia / No significant difference on KRT1 expression among the groups |
| Keratin, type I cytoskeletal 10 (*KRT10)* | 1  (129) | No correlation found between malignant transformation (OL vs. OSCC) and KRT10 expression |
| Keratin, type I cytoskeletal 13 (*KRT13)* | 3  (40,126,130) | CK13 expression decreased from normal (100%), to hyperplastic OL (70.3%), to dysplastic OL (32.4%), and to OSCC (2.9%) (p < 0.05). CK13 was expressed in all adjacent normal mucosa specimens, hyperplastic lesions, and dysplastic lesions and in 88% of HNSCCs. The staining intensity did not differ between adjacent normal mucosa and the positive HNSCCs. |
| Keratin, type I cytoskeletal 14 (*KRT14)* | 4  (36,40,129,131) | No correlation was found between malignant transformation and the expression of CK14. The LI of CK14 in the OL was significantly increased with the severity of epithelial dysplasia (p<0.0001) and in OSCC patients, CK14 was overexpressed compared to normal (p<0.0001). The expression of CK14 was significantly higher in hyperplastic and in mild to moderately dysplastic OL than in severe dysplasia and OSCC (p < 0.05). |
| Keratin, type I cytoskeletal 17 (*KRT17)* | 2  (40,130) | CK17-positive expression gradually increased according to progression. A combination pattern of CK17/CK13 could be an appropriate biomarker of malignant transformation / The levels of KRT-17 were significantly lower in hyperplasia and in mild to moderate dysplasia than in severe dysplastic tissue and OSCC (p < 0.007 and p < 0.001). |
| Keratin, type I cytoskeletal 18 (*KRT18)* | 2  (129,132) | Detectable levels of Ck 18 increased from 1.0% in OL to 13.0% in dysplastic OL to 66.7% in OSCCs, with significant correlation with dysplastic transformation in OL (p<0.02) and in OSCCs (p<0.01) / CK18 positivity was seen in none of the normal mucosa, in 40% of OL, and in 50% of OSCC (p= 0.08). |
| Keratin, type I cytoskeletal 19 (*KRT19)* | 3  (126,129,131) | Detectable levels of Ck 19 linearly increased from OL to dysplastic OL to OSCCs, significantly correlated with dysplastic transformation in OL (p<0.02) and in OSCCs (p<0.01). CK 19 expression in mild dysplasia, severe dysplasia and OSCC were 0.8±1.3%, 27.6±34.4% and 48.0±34.62%, respectively, with a significant difference between mild dysplasia and OSCC (p < 0.01) |
| Keratin, type II cytoskeletal 4 (*KRT4)* | 1  (40) | The expression was significantly higher in hyperplastic and in mild to moderately dysplastic OL than in severe dysplasia and OSCC (p < 0.05). |
| Keratin, type II cytoskeletal 2 oral (*KRT76)* | 1  (133) | The positive staining significantly decreased across the transition from normal tissue (100% positive) to OL (44%) to OSCC (35%) (p <0.0001). |
| Keratin, type II cytoskeletal 8 (*KRT8)* | 3  (126,129,132) | Detectable levels of Ck 8/18 increased from 1.0% in OL to 13.0% in dysplastic OL to 66.7% in OSCCs / Expression of CK8 was seen in 20% of the OL and in 30% of OSCC. None of the normal mucosa stained for CK8 (p = 0.34) / The expression of CK8 was significantly different between normal and dysplasia (p=0.024) and between normal and OSCC (p=0.0001). |
| Keratin-associated protein 5-6 (*KRTAP5-6)* | 1  (129) | No correlation was found between malignant transformation and the expression of Ck 5/6 |
| Laminin subunit alpha-3 - Laminin-5 (*LAMA3)* | 1  (124) | There was no unequivocal expression of the adhesion molecules distinguishing between inflammatory tissue, OL, and OSCC. |
| Laminin subunit gamma-2 (*LAMC2)* | 1  (134) | Cases of LAMC2-positive OL had an approximately 14-fold risk of malignant progression compared to the LAMC2-negative OL cases (p = 0.003). |
| Probable D-lactate dehydrogenase, mitochondrial – LDH (*LDHD)* | 2  (135,136) | The mean serum LDH level progressively increased from control group to mild dysplastic lesions, to lesions with moderate dysplasia, well differentiated OSCC and moderately differentiated OSCC (p<0.001) / Mean salivary LDH level also progressively increased in normal, OL and OSCC groups |
| Dual specificity mitogen-activated protein kinase kinase 1 (*MAP2K1)* | 1  (76) | Significantly higher expression of pMEK1/2 were observed in OSCC developed from OL than in OL samples, suggesting that these proteins might be indicators of malignant transformation of OL. |
| Dual specificity mitogen-activated protein kinase kinase 2 (*MAP2K2)* | 1  (76) | Significantly higher expression of pMEK1/2 were observed in OSCC developed from OL than in OL samples, suggesting that these proteins might be indicators of malignant transformation of OL. |
| Mitogen-activated protein kinase 1 (*MAPK1)* | 1  (71) | Immunohistochemical reactivity significantly enhanced in High grade Dysplasia only compared to that in Leukoplakia without epithelial dysplasia (P< 0.05) |
| Mitogen-activated protein kinase 3 (*MAPK3)* | 2  (71,76) | OSCC patients tended to have significantly higher pERK1/2 expression than OL patients (p < 0.001) / pERK1/2 expression was significantly enhanced in severe dysplasia compared to non-dysplastic OL (p<0.05). |
| DNA replication licensing factor MCM3 (*MCM3)* | 1  (35) | MCM3 expression was lower in control and mild dysplasia compared to severe dysplasia and OSCC (p<0.001). |
| DNA replication licensing factor MCM7 (*MCM7)* | 1  (95) | Mcm7 IHC expression: Normal: 8/10 (LI:3.6%), OL: 30/34 (LI: 22.3%) and OSCC: 51/54 (LI: 45.9%) (p<0.01). |
| E3 ubiquitin-protein ligase Mdm2 (*MDM2)* | 2  (28,137) | 78% OSCCs, 52% OL and 0% normal were MDM2 positive / MDM2 expression was found more often in OSCC than in OL. |
| Hepatocyte growth factor receptor (*MET)* | 1  (88) | Univariate analysis revealed that the expression was associated with the risk of malignant transformation of OL (HR: 6.431 (2.372–17.450); p<0.001). |
| Proliferation marker protein Ki-67  (*MKI67)* | 10  (28,29,31,35–38,88,138,139) | Ki-67 expression progressively increased from normal to hyperplasia, non-dysplastic OL to dysplasia and OSCC, and in OSCC increased according to loss of differentiation (p<0.01) and it was overexpressed, compared to normal (p<0.0001) / The mean Ki-67 LI in the OSCC group was 25.00 ± 20.82, 35.75 ± 44.52 on the OL group, whereas normal oral mucosa showed 30.00 ± 23.09 / High expression of Ki-67 was observed in 44% of normal mucosa, in 53% of OL and in 50% of OSCC / Positive expression for Ki-67 was 4.7± 1.8% in normal mucosa, 6.4 ± 3.3% in OL, and 14.9±4.9% in OSCC (p < 0.05) / No difference between normal and OL Ki-67 expression (p>0.05) / Significant difference in terms of malignant progression comparing the expression between normal mucosa and OL. |
| DNA mismatch repair protein Mlh1 (*MLH1)* | 1  (140) | Significantly lower expression seen in OL, which further decreases in OSCC as compared to normal oral mucosa. hMLH1 can be used as a reliable biomarker for malignant transformation. |
| Interstitial collagenase (*MMP1)* | 1  (123) | Significantly overexpressed in OSCC compared to normal mucosa (P =.007). However, there were no differences in OL vs healthy mucosa (P =.749). |
| 72 kDa type IV collagenase (*MMP2)* | 1  (106) | Logistic regression revealed MMP-2 as one of the best markers for distinguishing OL and OSCC patients from healthy individuals. |
| Matrix metalloproteinase-9 (*MMP9)* | 1  (106) | Significantly elevated in OL serum and further increased in OSCC compared to controls. Highly correlated with disease progression (p<0.001) |
| Metallothionein-2 (*MT2A)* | 1  (70) | There was a significant increase in MT2A positivity comparing normal oral mucosa to severe dysplasia and OSCC (p<0.01) |
| Serine/threonine-protein kinase mTOR (*MTOR)* | 1  (71) | pmTOR expression was significantly enhanced in severe dysplasia compared to non-dysplastic OL (p<0.05) and in OSCC compared to non-dysplastic OL (p<0.01). |
| Microtubule-associated tumor suppressor 1 (*MTUS1)* | 1  (141) | Detectable in the entire epithelium with strong staining in normal tissues. Significant reduction in staining in OL and OSCC (p=0.018 and p=0.0007). |
| Mucin-1  (*MUC1)* | 1  (142) | Higher frequency for positive expression in OSCC, while higher negative expression was seen in OL and normal groups with statistically high significant difference among groups (p = 0.002) |
| Mucin-4  (*MUC4)* | 2  (142,143) | MUC4 expression progressively increased from OL to OSCC, especially among the well-differentiated. MUC 4 may be a useful marker for OL and OSCC / MUC4 could act as marker of malignant progression given their rise in immunoexpression from normal mucosa to OL to OSCC. |
| Myc proto-oncogene protein (*MYC)* | 1  (144) | c-myc expression increased according to advanced grades of atypia. Statistical difference was found between OL and carcinoma in situ and OSCC. |
| Nestin  (*NES)* | 1  (145) | 50% of normal samples presented mild staining. 35% of OL samples were nestin negative, 42% presented mild staining and 23% were moderately stained. 7% of OSCC cases showed moderate expression, 33% showed mild expression, and 60% failed to express nestin. |
| Nuclear factor NF-kappa-B p105 subunit - NF-kB (*NFKB1)* | 2  (48,49) | The immunoreactivity of NF-κB howed a significant association with worsening histology (p<0.001). Increased expression and translocation of NF-κB was observed, suggesting the validation of NF-κB as participants of oral carcinogenesis / There was a gradual increase in NF-κB expression among the groups, with statistically significant difference in the OSCC group compared with the others (p<0.05). |
| Nucleotide-binding oligomerization domain-containing protein 1 (*NOD1)* | 1  (89) | NOD1 expression correlated significantly to diagnostic category (p <0.001). NOD1 may serve as novel and potential biomarkers for development and progression of OSCC. |
| Nitric oxide synthase, inducible (iNOS)  (*NOS2)* | 3  (146–148) | 1) Significant difference in the iNOS immune staining between normal and OSCC groups (p = .0280) / 2) A significantly higher percentage (83.3%) of OSCC patients showed iNOS expression compared to OL patients (73.3%) (p< 0.001). Control samples were iNOS negative / 3) Normal tissues were iNOS negative, 40% of OL cases were iNOS positive while 66,6% of OSCC cases were positively stained for iNOS. |
| Neurogenic locus notch homolog protein 1  (*NOTCH1)* | 1  (149) | A decrease on nuclear Notch1 expression (p<0.001) and an increase on membranous Notch1 expression (p=0.002) were observed in the OSCC samples compared to their matched OL samples. |
| GTPase Nras (*NRAS)* | 1 (71) | NRAS expression was significantly decreased in OSCC compared to non-dysplastic OL, mild and severe dysplasia (p<0.001). |
| BDNF/NT-3 growth factors receptor (*NTRK2)* | 1  (69) | Normal mucosa and OL showed similar expression of TrkB but differed on p‐TrkB expression. OL had a higher score compared to normal mucosa. Also, TrkB expression was increased in dysplastic OL compared to non‐dysplastic OL. OSCC presented increased expression of TrkB compared to normal mucosa and OL. |
| Proliferating cell nuclear antigen (*PCNA)* | 3  (28,31,150) | PCNA progressively increased from hyperplasia to dysplasia and OSCC, and in OSCC increased according to loss of differentiation / PCNA can differentiate between normal and OSCC but it does not seem a good marker in delineating non-dysplastic OL from dysplasia. Thus, PCNA absolute reliability is still questionable / No significant difference in PCNA expression was observed among the groups |
| Programmed cell death protein 1 - PD-1 (*PDCD1)* | 2  (45,93) | PD-1 expression tended to increase in association with malignant degrees of the oral epithelial lesions / High PD-1 expression was detected in the epithelial (p = 0.001) and subepithelial layers (p = 0.005) of transformed OL (T-OL) compared to non-transformed OL (N-OL). The overexpression in both tissue layers was significantly associated to malignant transformation (pE = 0.0001, pS = 0.02). |
| Podoplanin  (*PDPN)* | 4  (58–60,88) | 1) OSCC, OL and normal oral mucosa showed PDPN positivity of 100%, 76% and 60%, respectively (p = 0.003) / 2) Control samples were negative, 59.4% of OL cases and 82% of OSCC samples were PDPN positive (p = 0.00) / 3) There was an increase of PDPN expression scores from mild (1.32 ± 0.43) to severe dysplasia (3.03 ± 0.33), as well as from well (1.03 ± 0.74) to poorly differentiated (7.07 ± 0.86) OSCC (p < 0.01) / 4) In terms of malignant progression showed statistical significance |
| Phosphatidylethanolamine-binding protein 1 – RKIP (*PEBP1)* | 1  (151) | There was no statistically significant difference between RKIP expression in adjacent non‑cancerous tissues and oral leukoplakia lesions (P=0.518). However, a significant decrease in RKIP expression was noted in TSCC samples compared with either adjacent non‑cancerous tissues (P=0.000) or oral leukoplakia lesions (P=0.033). |
| Pituitary homeobox 1  (*PITX1)* | 1  (152) | PITX1 expression levels were significantly decreased in OL compared with the normal oral mucosa (p<0.001) and were further decreased in OSCC (p<0.001). PITX1 may serve as a novel biomarker for predicting prognosis in OL. |
| Piwi-like protein 2  (*PIWIL2)* | 1  (153) | Expression of PIWIL2 in patients with OL was found to be strongly prognostic of malignant transformation (p< 0.001), suggesting that PIWIL2 might play a role in the early steps of oral cancer tumorigenesis. |
| Periostin  (*POSTN)* | 2  (54,154) | Periostin expression in OSCC was significantly higher than in OL (p<0.01). The serum periostin levels in OL and OSCC patients were significantly higher than in healthy subjects (p < 0.05) / 90.5% of OSCC cases showed positive periostin expression significantly higher compared with OL (p = 0.003). Mild dysplasia showed significantly higher periostin expression compared to moderate and severe dysplasia (p = 0.055). Periostin is not a conceivable marker to predict the malignant transformation of OL. |
| POU domain, class 5, transcription factor 1 (*POU5F1)* | 1  (155) | OCT4 expression was sparse and insignificant. |
| Prostaglandin G/H synthase 2 - COX-2 (*PTGS2)* | 5  (46–49,88) | A significant increase in expression was seen from control through OL to OSCC (p<0.001). COX-2 expression increased as the severity of lesion progressed from normal epithelium to hyperplasia, dysplasia to invasive cancer, with a statistically significant difference between moderate dysplasia and control (p = 0.0163). OSCC tissues expressed significantly more COX-2 than OL and the expression was very low in normal oral tissues. |
| RAF proto-oncogene serine/threonine-protein kinase - Raf-1 (*RAF1)* | 1  (76) | There was no significant difference between OL and OSCC groups in pRaf-1 expression (p > 0.05). |
| Retinoblastoma-associated protein – pRb (*RB1)* | 4  (28,29,33,34) | 1) Most of the lesions stained positively for pRB / 2) pRb remained normal / 3) pRb positive staining was observed in 34.3% of OSCC cases, 36% of OL and in 93% of normal oral mucosa, with a strong correlation between the expression of pRb in OSCCs and in OL (p<0.008) / 4) Significant loss of pRb observed in transition from hyperplasia to dysplasia (p=0.005) and from OL to OSCC (OR = 2.938, p=0.001) |
| Receptor-interacting serine/threonine-protein kinase 2 (*RIPK2)* | 1  (89) | The current data indicate that RIP2 may serve as a novel and potential biomarker for development and progression of OSCC. |
| 40S ribosomal protein S6 (*RPS6)* | 1  (69) | No correlation with disease progression |
| Protein S100-A7  (*S100A7)* | 2  (99,156) | There was significant increase in S100A7 expression in normal, hyperplasia, dysplasia and HNSCC tissues (p<0.001). Nuclear S100A7 may be associated with increased transformation risk of oral premalignant lesions and recurrence in HNSCC / Compared to normal tissues, the expression of S100A7 was elevated in OL and OSCC (p<0.001) and correlated with the malignancy of these lesions. |
| E-selectin (*SELE)* | 1 (106) | No correlation with disease progression |
| Antileukoproteinase  (*SLPI)* | 1  (157) | It was observed a negative association between SLPI expression and the pathological differentiation of oral tissues from OL to OSCC (r=-0.4922, p<0.05). The overexpression of SLPI protein is negatively associated with the grade of OL and it may have a potential role in prediction of malignant transformation of OPMD |
| Mothers against decapentaplegic homolog 4 (*SMAD4)* | 1  (158) | Significantly lower expression in OSCC than in the paired malignant transformed OL (p<0.001). Strong expression in 77.3% of transformed OL and 39.4% of untransformed OL (p=0.002). |
| Zinc finger protein SNAI1- SNAIL (*SNAI1)* | 1  (97) | No differences in SNAIL immunoexpression among NOM, OL, OLD, OSCC and LN specimens |
| Transcription factor SOX-2 (*SOX2)* | 2  (155,159) | 1) SOX2 expression showed statistically significant difference among study groups (p<0.03), with increasing expression from OL to OSCC, suggesting its use as a potential marker of proliferation / 2) SOX2 expression was found to significantly increase with the grade of dysplasia and to be negative in normal adjacent epithelia. Positive SOX2 expression significantly predicted oral cancer risk |
| Osteonectin (*SPARC)* | 1  (160) | OL, carcinoma in situ, and early invasive SCC had more SPARC-positive cells than normal epithelium. This may suggest that some transformation of cells provoke SPARC expression. |
| Osteopontin  (*SPP1)* | 1  (161) | 55% of mild dysplasia cases, 0% of moderately dysplastic cases and 80% of severely dysplastic OL were OPN positive with no statistically significant difference compared to normal epithelium. OPN expression does not seem a relevant diagnostic and prognostic marker for oral mucosal dysplasia and malignant transformation |
| Cohesin subunit SA-2  (*STAG2)* | 1  (162) | All lesions and normal mucosa showed high expression, with no association with any clinical, pathological, or prognostic data. |
| Signal transducer and activator of transcription 3  (*STAT3)* | 1  (71) | pSTAT3 immunoreactivity was detected in the cytoplasm and nuclei of epithelial cells of OLs and OSCC, with no statistical difference among the groups. |
| Stathmin  (*STMN1)* | 1  (163) | Statistically significant increased expression of stathmin was observed in OSCC group (2.50 ±1.33) compared to the OL group (2.11 ± 1.54) and the normal tissue (0.00) (p = 0.0001). |
| Angiopoietin-1 receptor  (*TEK)* | 1  (123) | TEK expression in healthy tissue did neither significantly differ in comparison to OSCC (p =0.595) nor to OL (p =0.057). |
| Protransforming growth factor alpha (*TGFA)* | 1  (111) | The mean Cytoplasmic optical density of PL and OSCC were significantly higher than that of normal mucosa (p<0.01). There was no significant difference in the mean COD of PL and OSCC (p>0.05) / The area and intensity of staining of TGF-a increased significantly in OL and OSCC compared to normal mucosa (p< 0.05). |
| Transforming growth factor beta-1 proprotein (*TGFB1)* | 4  (53,91,106,164) | There was no significant difference between salivary TGF-β levels in OL patients and the control group (p>0.05). Significantly fewer TGF-𝛽1 cells were seen in OSCC than in OL (p= 0.0001). Increasing levels of serum TGF-𝛽1 were observed from normal mucosa, to OL, to OSCC samples (p<0.00). TGF-β1 was significantly elevated in OL and further increased in OSCC compared to controls, highly correlated with disease progression (p<0.001). Logistic regression revealed TGF-β1 as one of the best markers for distinguishing OL and OSCC patients from healthy individuals. |
| Transforming growth factor beta-2 proprotein  (*TGFB2)* | 1  (53) | OL presented a significantly higher tissue expression of TGF-β2 than the control group (p<0.05), but not in saliva. The expression of this protein in OL was similar than the OSCC group (p>0.05). |
| Transforming growth factor beta-3 proprotein  (*TGFB3)* | 1  (53) | OL presented a significantly higher tissue expression of TGF-β3 than the control group (p<0.05), but not in saliva. The expression of this protein in OL was similar than the OSCC group (p>0.05). |
| TGF-beta receptor type-1 (*TGFBR1)* | 1  (165) | Homogeneous, moderate, and intense cytoplasmic expression of T𝛽RI was observed in OSCC, OL and normal tissues. |
| TGF-beta receptor type-2 (*TGFBR2)* | 1  (165) | T𝛽RII expression gradually decreased according to the progression of carcinogenesis (from normal specimens through OL to OSCC). Therefore, T𝛽RII may be used as predictor of tumorigenesis and severity. |
| TGF-beta receptor type-3 (*TGFBR3)* | 1  (165) | T𝛽RIII expression gradually decreased according to the progression of carcinogenesis (from normal specimens through OL to OSCC). Therefore, T𝛽RIII may be used as predictor of tumorigenesis and severity. |
| Protein-glutamine gamma-glutamyltransferase E  (*TGM3)* | 1  (40) | Expression significantly higher in hyperplastic and in mild to moderately dysplastic OL than in severe dysplasia and OSCC (p < 0.05). |
| Tumor necrosis factor  (*TNF)* | 5  (50,51,121,166,167) | A significant difference was found in salivary TNF-α level between OSCC (133.3±15.0) and OL (99.8±7.9), OSCC and controls (83.3±5.5), and OL and controls (p = 0.000). Salivary concentration of TNF-α was higher in OSCC patients than in controls (p=0.0012) and non-dysplastic OL patients (p =0.0492). Serum TNF-α level was significantly higher in control than in OL and OSCC (p≤0.05). Increasing levels of salivary TNF-α from control (5.75 ±3.98) to OL (28.96 ± 20.94) to OSCC (63.94 ± 56.05) (p<0.01). Salivary TNF-α of OL vs healthy control and OSCC vs healthy controls with an AUC of 0.968 and 0.997, respectively (p<0.001). |
| Tumor necrosis factor receptor superfamily member 10A (*TNFRSF10A)* | 1  (168) | Expression of DR4 receptor was not significantly altered during oral cancer progression |
| Tumor necrosis factor receptor superfamily member 10B (*TNFRSF10B)* | 1  (168) | Expression of DR5 receptor was not significantly altered during oral cancer progression |
| Tumor necrosis factor receptor superfamily member 10C (*TNFRSF10C)* | 1  (168) | Expression of DcR1 receptor was not significantly altered during oral cancer progression |
| Tumor necrosis factor receptor superfamily member 10D (*TNFRSF10D)* | 1  (168) | A partial loss of DcR2 expression is also noted in premalignant and malignant oral epithelia compared to normal oral epithelia |
| Tumor necrosis factor ligand superfamily member 10 (*TNFSF10)* | 1  (168) | TRAIL is constitutively expressed in normal oral mucosa, but its expression is gradually lost in OL and OSCC. Thus, loss of TRAIL expression is an early event during oral carcinogenesis. |
| Cellular tumor antigen p53 (*TP53)* | 15  (27–32,37,39,63,81,88,102,137,138,169) | The expression of p53 progressively increased with progression toward malignancy from normal mucosa (5-14%) to hyperplasia to nondysplastic to dysplastic OL (33-67%) and it was even higher in OSCC (59-72.5%) (p< 0.05) / The mean p53 LI in the OSCC group was 66.25 ± 25.62, 75.00 ± 16.83 on the OL group, whereas normal oral mucosa showed 20.00 ± 5.77 (p>0.05) / Accumulation of p53 was present in 88% of hyperplasia, 62% of dysplasia and 19% OSCC. p53 positivity was significantly higher in patients with OL than in OSCC patients (p = 0.0001). OL patients who show p53 expression have a higher risk of developing OSCC than those who do not show p53 / Comparing the expression of p53 between normal mucosa and OL in terms of malignant progression, statistical significance was observed. The set of p53 and CA9 showed the highest accuracy (0.881) combined with age and dysplasia. |
| Tumor protein 63 - p63 (*TP63)* | 3  (32,36,170) | 1) The average LI for OSCC was 82.8% and for OL the LI was 65.15%, demonstrating statistically significant difference between OSCC and OL (p<0.05) / 2) The LI of p63 in the OL was significantly increased with the severity of epithelial dysplasia and in OSCC compared to normal samples (p<0.0001). The p63-LI of the OL with malignant transformation was significantly higher than in OL with no malignant transformation (p<0.01) /3) There was a significant increase in the mean p63 LI of OSCC compared to OL (p < 0.05), and normal mucosa (p < 0.01), and in OL compared to normal mucosa (p<0.01). |
| Twist-related protein 1 (*TWIST1)* | 1  (96) | Differences in Twist expression were seen among all the groups (p ≤ 0.004), between normal mucosa and OSCC (p ≤ 0.004) and mild dysplasia and OSCC (p ≤ 0.018). A possible value of Twist was showed in the risk prediction of oral epithelium malignant transformation. Also, Twist may be associated since early stages of oral carcinogenesis. |
| Vascular endothelial growth factor A (*VEGFA)* | 2  (120,171) | The mean±SEM level of VEGF was gradually upregulated from healthy (70.35±4.931), OL (101.4±10.82), to OSCC individuals (158.8±10.03) (p<0.05) / VEGF-A expression was significantly increased from OL through dysplasia (mild and moderate; all p< 0.05) to OSCC. |
| Protein Wnt-5a  (*WNT5A)* | 1  (155) | Normal tissue had significantly lower expression of WNT5A compared to OSCC and OL. WNT5A positivity also significantly increased from OL to OSCC, with higher expression in peripheral tumor cells of tumor islands aiding in invasion, suggesting its use as a marker of tumor invasion and transformation from OL to OSCC. |
| 14-3-3 potein zeta/delta (*YWHAZ)* | 1  (172) | Significant increase in 14-3-3ζ expression was observed in different stages of oral tumorigenesis (normal to hyperplasia, dysplasia and OSCC, p< 0.001). |

IHC: Immunohistochemistry; LI: Labeling index; OL: Oral Leukoplakia; OSCC: Oral Squamous Cell Carcinoma; Refs: References

Appendix 5. Summary of descriptive characteristics of included studies assessing proteins in tissues (n=124)

| Author, year, country | Study design | Sample size (M/F) | Gene | Protein | Method | Main results | Conclusion |
| --- | --- | --- | --- | --- | --- | --- | --- |
| Agarwal et al., 1998  India | Cross-sectional | 51 OSCCs (34/19)  25 OL (19/6)  40 healthy control | *CDKN1A*  *TP53* | p21  p53 | IHC | p21 immunoreactivity was predominantly nuclear. 68.6% OSCCs and 60% OL were p21 positive. Normal oral tissues did not show p21 immunoreactivity. The expression of p21 and p53 proteins was observed in 44% OL and 58.8% OSCCs. | Heterogeneity in p21 expression was observed in OSCCs and in OL, suggesting that alterations in p21 expression are an early event in oral oncogenesis. |
| Agarwal et al., 1999  India | Cross-sectional | 65 OSCCs (46/19)  33 OL (27/6)  30 healthy control | *MDM2*  *TP53* | MDM2  p53 | IHC | 78% OSCCs, 52% OL and 0% healthy control were MDM2 positive. 60% OSCCs (*p*=0.021) and 49% OL (*p*=0.001) showed co-overexpression of MDM2 and p53. In OSCCs, the co-expression was associated with advanced tumour stages (*p*=0.0009) and lymph node metastases (*p*=0.0325). | Significant association between co-expression of MDM2 and p53 proteins in OL suggests that these alterations are early events and may be involved in preinvasive stages of oral tumorigenesis. |
| Aiswarya et al., 2019  India | Cross-sectional | 30 OSCCs  25 OL  10 healthy control | *PDPN* | Podoplanin | IHC | OSCC, OL and normal oral mucosa showed PDPN positivity of 100%, 76% and 60%, respectively (*p* = 0.003). The mean immunoreactive PDPN score in the OSCC group was 9.15 ± 3.54, 3.76 ± 3.19 on the OL group, whereas normal oral mucosa showed 0.83 ± 0.80 (*p <* 0.001). | A progressive and significant increase of PDPN expression is evident from normal oral mucosa to OL and OSCC. Increased PDPN expression correlates with higher grade of epithelial dysplasia, indicating increased risk of malignant transformation. |
| Ambatipudi et al., 2013  India/UK | Cross-sectional | 159 OSCCs (120/39)  61 OL (55/6)  35 healthy control | *KRT76* | Keratin 76 | RT-qPCR  IHC | RT-qPCR analysis revealed significant downregulation of KRT76 RNA in tumor compared to healthy control samples (*p* <0.0001). The frequency of KRT76 positive staining significantly decreased across the transition from normal tissue (100% positive) to OL (44%) to OSCC (35%) (*p* <0.0001). | Although it was observed KRT76 downregulation in patients with OSCC, future studies are still needed to assess the impact of KRT76 loss in predicting high-risk potentially malignant lesions of oral cavity. |
| Angelin et al., 2020  India | Cross-sectional | 20 OSCCs (12/8)  20 OL (15/5)  20 control | *BIRC5* | Survivin | IHC | Survivin positivity in normal mucosa was noted in 7 cases with weak expression. 14/20 OL cases were positive and 16/20 OSCC cases had a positive survivin expression. The expression of survivin among all the groups was statistically significant (p = 0.015) as well as in OSCC compared to normal mucosa (p= 0.01). | Survivin expression levels in OL and OSCC were significantly higher than that in normal oral tissues. Survivin can be identified as a useful tool for the identification of potentially malignant disorders at higher risk for progression into invasive carcinoma. |
| Aruldoss et al., 2016  India/Kingdom of Saudi Arabia | Cross-sectional | 20 OSCC (15/5)  20 OL (16/4)  40 healthy control (24/16) | *PTGS2* | COX-2 | IHC | The mean value of COX‑2 expression was 0 in negative control, 29.72 in positive control, 48.10 in OL group, and 59.18 in OSCC group. A very high significant increase in expression was seen from control through OL to OSCC. | It was observed an increased expression of COX‑2 from OL to OSCC. Therefore, COX‑2 could be used as an early detection marker in OL and a prognostic marker in OSCC. |
| Babiuch et al., 2020  Poland | Cross-sectional | 14 OSCC  10 dysplastic OL  21 non-dysplastic OL | *IL1A*  *IL6*  *CXCL8*  *TNF* | IL-1α  IL-6  IL-8  TNF-α | IHC | IL-1α was present more often within all layers of the epithelium in OSCCs than in OL. TNF-α  was present within all layers of the epithelium in almost all cases of OED and OSCC, and only in 1/3 of healthy control cases. IL-8 was present within all layers of the epithelium in almost 65% of OSCC cases and in none layer of healthy control specimens. | The most important biomarker of malignant transformation seems to be IL-8 since it was present within all layers of the epithelium in most OSCCs, was not present in epithelial cells of healthy control specimens and was observed in the smallest number of OL samples. |
| Bavle et al., 2020  India | Cross-sectional | 35 OSCC  35 OL | *TP63* | p63 | IHC | The average labeling index for OSCC was 82.8 while for OL the labeling index was 65.15%, demonstrating statistically significant difference between OSCC and OL (p < 0.05). | Progressive accumulation of p63‑immunopositive cells from OL to OSCC may be useful indicator of dysplastic change, serving as a biomarker of cancer progression. |
| Bernardes et al., 2014  Brazil | Cross-sectional | 22 OSCC (18/3)  15 OL (7/8)  11 Healthy control | *STAG2* | STAG2 | IHC | All lesions and healthy mucosa showed high expression of STAG2 (> 75 % positivity), with no association of STAG2 expression with any clinical, pathological, or prognostic data of the lesions. | The findings suggest that STAG2 loss is not a frequent event in oral carcinogenesis, and it cannot be used as a prognostic marker for the studied tumor types. |
| Buajeeb et al., 2009  Thailand | Cross-sectional | 16 OSCC (9/7)  30 OL (14/16)  10 Healthy control (4/6) | *CDKN2A* | p16 | IHC | Positive staining was found only in non dysplastic OL  and OSCC. There were no statistically significant differences in p16 expression among OSCC, OL with and without dysplasia, and healthy mucosa (*p*=0.08). | The results indicate that p16 expression cannot be used as a marker for oral mucosal dysplasia nor malignant transformation. |
| Chamorro-Petronacci et al., 2021  Spain and Brazil | Cross-sectional | 15 OSCC  15 dysplastic OL  14 non-dysplastic OL  16 healthy control | *BCL2* | Bcl-2 | IHC | Bcl-2 expression among healthy controls (23.94±19.16), non-dysplastic OL (28.40±25.87), dysplastic OL (31.21±25.47), and OSCC (24.63±22.68) was statistically different (p=0.000), although no bivariate analysis were performed between these groups. | The findings highlight the value of Bcl-2 as a biomarker for studying the malignant transformation of OPMD and the need for future research in cohort studies and with other convergent apoptotic signaling molecules. |
| Chaudhari et al., 2016  India | Cross-sectional | 30 OSCC (19/11)  30 OL (30/0)  30 Healthy control | *MLH1* | hMLH1 | IHC | hMLH1 positivity was seen in all healthy control samples, in 25 of 30 samples of OL and in 24 of 30 samples of OSCC. hMLH1 expression was significantly higher in control group than in OL and OSCC groups. hMLH1 expression was significantly higher in OL than in OSCC (*p* < 0.05). | Progressive decrease in hMLH1 expression was observed from control to OL and further to OSCC, and also in different clinical and histopathological stages of OL and OSCC. Altered expression of hMLH1 in OL may be an early event in carcinogenesis. |
| Chen et al., 2019  China | Cross-Sectional | 41 OSCC (27/14)  21 OL (13/8)  25 Healthy control (12/13) | *CD274* | PD-L1 | IHC | Positively stained OSCC vs OL vs healthy tissues were scored as follows: score 3 (61-100% intensity), 48.78% vs. 4.76% vs. 0. There was a significant difference in positive staining scores across the 3 groups (*p*<0.0001). PD-L1 density was significantly stronger in both OSCC and OL compared with normal control (*p*<0.0001). | The percentage of PD-L1-positive OL samples was higher than in the normal tissues, suggesting that PD-L1 has potential as a biomarker for OLK. PD-L1 expression  in OSCC and OLK was closely associated with disease progress. |
| De Freitas Silva et al., 2014  Brazil | Cross-sectional | 20 OSCC  30 OL  10 Normal  (41/19) | *TWIST1*  *CDH1* | Twist  E-cadherin | IHC | Differences in Twist and E-cadherin expression were seen among all the groups (*p* ≤ 0.004), between normal mucosa and OSCC (*p* ≤ 0.004) and mild dysplasia and OSCC (*p* ≤ 0.018). Also, significant differences in E-cadherin staining were observed between normal mucosa and dysplasia (*p* ≤ 0.002). | A possible value of Twist and E-cadherin was showed in the risk prediction of oral epithelium malignant transformation. Also, Twist and E-cadherin may be associated since early stages of oral carcinogenesis, suggesting that Twist may play an important role in oral carcinogenesis. |
| De Vicente et al., 2019  Spain | Cross-sectional | 125 OSCC  55 OL (26/29) | *SOX2* | SOX2 | TMA  IHC | Normal adjacent epithelia showed negative SOX2 expression. SOX2 expression was found to significantly increase with the grade of dysplasia. Positive SOX2 expression significantly predicted oral cancer risk either considering SOX2 > 10 (log-rank test, *p* = 0.02) or SOX2any (log-rank test, *p*= 0.01) as cut-off points. According to TCGA, SOX2 gene alterations were present in a total of 38 (22%) of 172 OSCC patients. | SOX2 expression is an important feature in early stages of oral tumorigenesis and it is potentially useful as biomarker for oral cancer risk assessment. SOX2 expression emerges as an important determinant in the pathogenesis of OSCC, contributing to tumor initiation and acquisition of an invasive phenotype. |
| Ding et al., 2012  China/USA | Cross-sectional | 80 OSCC  27 OL  13 Normal | *MTUS1/ ATIP* | MTUS1/ ATIP | IHC  RT-qPCR | In normal tissues, MTUS1/ATIP was detectable in the entire epithelium with strong staining. Significant reduction in MTUS1/ATIP staining was observed in OL and OSCC (*p*=0.018 and *p=*0.0007). Among OSCC cases, MTUS1/ATIP levels were significantly lower in poorly and moderately differentiated cases (*p*=0.04 and *p=*0.0006, respectively). | The results suggest a critical role of MTUS1/ATIP in the tumorigenesis of OSCC, and MTUS1/ATIP may serve as a biomarker or a novel therapeutic target for patients with OSCC. |
| Ding et al., 2018  China/Australia/USA | Retrospective cohort | 19 OSCC (5/14)  78 OL (33/45) | *NOTCH1* | Notch1 | IHC | Higher nuclear Notch1 expression was observed in untransformed OLs than in OSCCs (*p*=0.001). A decrease on nuclear Notch1 expression (*p*<0.001) and an increase on membranous Notch1 expression (*p*=0.002) were observed in the OSCC samples compared to their matched OL samples. The expression of membranous Notch1 was associated with OSCC‑free survival (*p*=0.001). | Further studies are necessary to determine how membranous Notch1 expression leads to oral tumorigenesis and OSCC progression. |
| Dong et al., 2014  China | Cross-sectional | 21 OSCC (15/6)  25 OL (14/11) | *AURKA*  *RAF1*  *MAP2K1*  *MAP2K2*  *MAPK3* | Aurora A pRaf-1 pMEK1/2  ERK1/2  pERK1/2 | IHC | OSCC patients tended to have significantly higher Aurora A, pMEK1/2 and pERK1/2 expression than OL patients (*p*=0.008, *p* = 0.033 and *p* < 0.001, respectively). There was no significant difference between OL and OSCC groups in pRaf-1 and ERK1/2 expression (*p* > 0.05). | Significantly higher expression of Aurora A, pMEK1/2 and pERK1/2 were observed in OSCC developed from OL than in OL samples, suggesting that these proteins might be indicators of malignant transformation of OL. |
| Duś-Ilnicka et al., 2020  Poland | Cross-sectional | 29 OSCC (17/12)  31 OL (19/12) | *SPARC* | Osteonectin | IHC | Expression of SPARC was negative in 11.54% of OSCC patients. Scores 1, 2, 3 and 4 expression were observed in 34.63%, 30.77%, 15.38%, and in 7.69% of OSCC patients, respectively. In OL patients, lack of expression (Score 0) was observed in 66.67%. Score 1 was observed in 30.00%, Score 2 was observed in 1 case, and Scores 3 and 4 were not present in the whole group of OL. With the progression of severity of the oral mucosa pathological changes, increased production of osteonectin was observed. | A higher expression of SPARC was demonstrated according to pathological changes in the oral mucosa, which progressed from different histopathological stages in OL to OSCC, compared to normal tissue. Osteonectin production may be induced by dysplastic cells in the initial stages of neoplastic development, but it stopped at this stage and was not an indicator of malignancy of OSCC. |
| Dwivedi et al., 2020  India | Cross-sectional | 15 OSCC  15 dysplastic OL  15 non-dysplastic OL  5 Normal | *BCL2* | Bcl-2 | IHC | The mean labeling index (LI) in normal tissue was 14.16 ± 6.43, in non-dysplastic OL was 15.30 ± 9.94, in dysplastic OL was 33.78 ± 14.29 and in OSCC was 15.60 ± 18.47 with maximum in poorly differentiated OSCC (30.92±23.16) and minimum in moderately differentiated OSCC (2.28±1.73). | Bcl-2 expression in dysplastic and non-dysplastic oral epithelium suggests that it may play an important role only in early tumorigenesis and may decrease with increased maturation according to differentiation within malignant epithelial cells. |
| Eversole & Sapp, 1993  USA | Cross-sectional | 20 OSCC  30 OL  10 Normal | *MYC* | c-myc | IHC | In most benign keratoses, c-myc expression was confined to the basal cell layer. Among the dysplasia, carcinoma in situ and OSCC cases, c-myc expression was regularly found in all strata and the expression increased according to advanced grades of atypia. Statistical difference was found between OL and carcinoma in situ and OSCC. | The expression c-myc in advanced stages of cytological atypia may not represent progressive cell transformation. However, this oncogene product could have important implications in the initial phases of malignant transformation in OL. |
| Fan et al., 2006  China | Cross-sectional | 67 OSCC  79 OL  10 normal | *CDKN1C*  *TP53*  *HSPD1* | P57(kip2)  p53  hsp60 | IHC | P57(kip2) expression progressively decreased from normal mucosa to OL and it was even lower in OSCC (*p*< 0.05). Negative expression of P57(kip2) was associated with advanced tumor size, occurrence of lymph node metastasis and advanced clinical stage (*p*< 0.05). p53 and hsp60 expression progressively increased from normal mucosa to OL and it was even higher in OSCC (*p*< 0.05). | Loss of P57(kip2) expression seems to be associated with carcinogenesis in oral mucosa. Thus, P57(kip2) may be a promising candidate as a progressive and prognostic biomarker in OSCC. |
| Feng et al., 2008  China | Cross-sectional | 76 OSCC (29/25)  49 OL (15/19)  20 Normal | *MCM7*  *CDC6* | Mcm7  Cdc6 | RT-PCR  IHC | Mcm7 mRNA expression: Normal: 1.932±0.104, OL: 2.448±0.103 and OSCC: 3.123±0.070 (p<0.01). Cdc6 mRNA expression: Normal: 1.798±0.096, OL: 2.448±0.117 and OSCC: 2.903±0.118 (p<0.01). Mcm7 IHC expression: Normal: 8/10 (LI:3.6%), OL: 30/34 (LI: 22.3%) and OSCC: 51/54 (LI: 45.9%) (p<0.01). Cdc6 IHC expression: Normal: 0/10, OL: 16/34 and OSCC: 33/54 (LI: 31.2%). | High expression of Mcm7 and Cdc6 were correlated with OSCC development. Expression and LI values of both proteins may help distinguish normal tissues from OL and OSCC. Mcm7 and Cdc6 expression may potentially become molecular markers for the early diagnosis and prognosis prediction of OSCC. |
| Fernández-Valle et al., 2016a  Spain | Retrospective cohort | 100 OSCC (64/36)  62 OL (35/27)  Normal | *KCNC4* | Kv 3.4 | IHC | Normal epithelium showed no Kv3.4 expression. 22% of OL lesions were Kv3.4-positive while 39% of OSCCs exhibited Kv3.4-positive expression. Patients with Kv3.4-positive lesions showed a higher progression risk than Kv3.4-negative (*p*=0.58). | Abnormal Kv3.4 expression is a clinically  relevant feature on OSCC tumorigenesis and malignant transformation, although expression does not seem to have a major impact on OSCC progression or outcome. |
| Fernández-Valle et al., 2016b  Spain | Retrospective cohort | 100 OSCC (64/36)  62 OL (35/27)  Normal | *KCNH2* | HERG1 | IHC  RT-qPCR | 47% of OSCCs and 36% of OL exhibited HERG1-positive expression, whereas normal epithelium did not expressed HERG1. There was not association between HERG1 expression and progression to cancer. HERG1 mRNA levels significantly increased in tumors compared with normal mucosa (*p* = 0.013). | Aberrant HERG1 expression occurs in early stages of oral tumorigenesis and increases during tumor progression. HERG1 expression emerges as a clinically relevant feature in OSCC progression. |
| Fillies et al., 2007  Germany | Cross-sectional | 192 OSCC (154/38)  140 OL (84/56) | *KRTAP5-6*  *KRT8*  *KRT18*  *KRT1*  *KRT10*  *KRT14*  *KRT19* | Keratin 5/6  Keratin 8/18  Keratin 1  Keratin 10  Keratin 14  Keratin19 | TMA  IHC | Detectable levels of Ck 8/18 increased from 1.0% in OL to 13.0% in dysplastic OL to 66.7% in OSCCs. Detectable levels of Ck 19 increased from 9.4% in OL to 27.2% in dysplastic OL to 40.6% in OSCCs. Both Ck were significantly correlated with dysplastic transformation in OL (*p*<0.02) and in OSCCs (*p*<0.01). No correlation was found between malignant transformation and the expression of Ck 5/6, Ck 14 and Ck 10. | Important change in the cytokeratin expression pattern for Ck 8/18 and 19 may play a role in the initiation and progression of OSCC and its precursor lesions. |
| Foki et al., 2020  Austria | Cross-sectional | 10 OSCC  10 OL  10 Normal | *ITGA2*  *MMP1*  *TEK* | ITGA-2 MMP-1  TEK | IHC | Compared to healthy mucosa, ITGA-2 was significantly overexpressed in OL (*p* = 0.002) as well as in OSCC (*p* = 0.003). MMP-1 was significantly overexpressed in OSCC compared to normal mucosa (*p* =0.007) but there were no differences in OL vs healthy mucosa (*p* =0.749). TEK expression in healthy tissue did neither significantly differ in comparison to OSCC (*p* =0.595) nor to OL (*p* =0.057). | On the posttranslational level, ITGA-2 and MMP-1 were significantly overexpressed in tumor tissue. These changes seem to influence various transformation states of oral mucosa and thus contribute substantially to early oral carcinogenesis after cigarette smoke exposure. |
| Girod et al., 1998  Germany | Cross-sectional | 103 OSCC  90 OL | *TP53*  *MDM2*  *RB1*  *MKI67*  *PCNA* | p53  MDM2  pRb  Ki-67  PCNA | IHC | p53 expression progressively increased from hyperplasia to dysplasia and OSCC, but with no significant correlation with tumor grade. MDM2 expression was found more often in OSCC than in OL. Most of the lesions stained positively for RB. Ki-67 and PCNA progressively increased from hyperplasia to dysplasia and OSCC, and in OSCC increased according to loss of differentiation. | Alterations in p53 and Rb genes may be potentially useful biomarkers in oral carcinogenesis. Overexpression of p53 combined with proliferative activity predicts a less favorable course of disease in OSCC. |
| Gonçalves et al., 2017  Brazil | Cross-sectional | 20 OSCC (14/6)  80 OL (40/60)  20 Normal | *HLA-G HLA-E*  *CD274*  *IL10*  *TGFB1*  *TGFB2*  *TGFB3* | HLA-G  HLA-E,  PD-L1  IL-10  TGF-β1, 2 and 3 | IHC | OL presented a significantly higher expression of HLA-G, -E, IL-10, TGF-β2 and -β3 than the control group (*p*<0.05). The expression of these proteins in OL was similar than the OSCC group (*p*>0.05). There was no significant difference between salivary sHLA-G, IL-10 and TGF-β levels in OL patients and those of the control group (*p*>0.05). | OL is characterized by high expression of HLA-G, -E, PD-L1, IL-10, TGF-β2 and -β3. Overexpression of these mediators in OL indicates the immune evasion potential of this lesion, which seems independent of cytological and proliferation/apoptosis status. |
| Guan et al., 2019  China | Cross-sectional | 45 OSCC (25/20)  40 OL  30 Normal | *POSTN* | Periostin | IHC  RT-qPCR | Periostin expression in OSCC was significantly higher than in OL (*p*<0.01) and was correlated with tumor differentiation, TNM staging and lymph node metastasis (*p*< 0.05). Compared to normal mucosa, mRNA expression of periostin in OL and OSCC significantly increased (*p*<0.01). The serum periostin levels in OL and OSCC patients were significantly higher than in healthy subjects (*p* < 0.05). | Aberrant expression of periostin occurs in early stages of morphological changes of OL, suggesting that the increase in periostin expression may be an early event in the carcinogenesis of OL. |
| Hamidi et al., 2000  Canada/ Finland | Cohort | 11 OSCC  29 OL  11 Normal | *ITGB6* | α_v_β_6_ integrins | Immunofluorescence | 41% of OL specimens expressed α_v_β_6_ integrin. 27% of the dysplasia specimens expressed α_v_β_6_ integrin, while 86% of hyperkeratosis, hyperplasia and atypia expressed it. 80% of OSCC were positive when α_v_β_6_ integrin antibody was used, and 90% were positive when stained with α_v_ integrin antibody only. All the patients who progressed expressed α_v_β_6_ integrin. | OL specimens express α_v_β_6_ integrin that may be associated to epithelial repair, inflammation or malignant transformation. Expression of α_v_β_6_ integrin seems to be necessary but not sufficient for malignant transformation. |
| He et al., 2008  China | Cross-sectional | 32 OSCC (23/9)  19 OL (12/7)  10 Normal (7/3) | *ATM* | ATM | IHC  PCR | All normal samples were moderately stained, and in OL samples, 12 presented moderate and 7 high ATM expression. In OSCC, ATM expression was negative in 5 samples, 5 were mild positive, 12 were moderately positive, and 10 were strongly positive. Significant difference was observed in ATM expression between OL and controls (*p*=0.030). | Overexpression of ATM may be one of the early events in carcinogenesis of OSCC. ATM inactivation might be one of the genetic alterations during the progression of OSCC. ATM might be a possible genetic marker for early-onset OSCC. |
| Herrera Costa et al., 2019  Brazil | Cross-sectional | 30 OSCC (23/7)  27 OL (14/13) | *ALDH1A1*  *ALDH2* | ALDH1A1  ALDH2 | IHC | ALDH1A1 positive expression occurred in the basal layer and in the suprabasal layer in 100% and 83.3% OL cases, respectively. For ALDH2, in both layers, 48.1% of the cases were positive. In the OSCC cases, ALDH1A1 expression were negative in 70% of the cases, mostly in moderately differentiated tumors, whereas ALDH2 expression was positive in all cases. | ALDH1A1 is higher expressed when compared with ALDH2 in OL. However, ADLH1A1 expression is low, with a significant increase of ALDH2 expression in OSCC. The results suggest that these proteins might be involved in oral carcinogenesis. |
| Hu et al., 2015  China/ USA | Cross-sectional | 85 OSCC (51/34)  32 OL  85 adjacent non‑cancerous | *PEBP1* | RKIP | IHC | 43.5% of the OSCC demonstrated high RKIP expression, while 71.8% of the corresponding adjacent non‑cancerous tissues and 65.6% of the OL demonstrated high RKIP expression. There was a significant decrease in RKIP expression in OSCC samples compared with adjacent non‑cancerous tissues (*p*=0.000) and OL lesions (*p*=0.033). | The loss of RKIP expression in OSCC seems to be associated with clinicopathological characteristics of cancer aggressiveness. Loss of RKIP expression is associated with poor survival time and may act as a potential biomarker of prognosis in OSCC patients. |
| Humayun & Prasad, 2011  India | Cross-sectional | 4 OSCC (3/1)  4 OL (4/0)  2 normal (2/0) | *TP53*  *MKI67* | p53  Ki-67 | IHC | The mean p53 LI in the OSCC group was 66.25 ± 25.62, 75.00 ± 16.83 on the OL group, whereas normal oral mucosa showed 20.00 ± 5.77. The mean Ki-67 LI in the OSCC group was 25.00 ± 20.82, 35.75 ± 44.52 on the OL group, whereas normal oral mucosa showed 30.00 ± 23.09. No difference comparing groups with each other for any protein. | The correlation between progression of oral epithelium from normal to OSCC and increased expression of these antigens suggest that they may be useful biomarkers of malignant transformation. |
| Jing et al., 2019  China | Cross-sectional | 298 OSCC (152/146)  62 OL  36 Normal | *MKI67* | Ki-67 | IHC | High expression of Ki-67 was observed in 16/36 (44%) of normal mucosa, in 33/62 (53%) of OL and in 149/298 (50%) of OSCC. Also, the results showed that high Ki-67 expression increased from mild dysplasia (66.7%) to moderate (73.3%) but it was decreased in severe dysplasia (60.5%). | There was upregulation of Ki-67 expression with tumor progression from normal epithelial mucosa, dysplasia and OSCC samples. Ki-67 expression increased from mild to moderate dysplasia. Also, highly expressed Ki‑67 may be an independent prognostic marker for OSCC. |
| Juneja et al., 2015  India | Cross-sectional | 30 OSCC (15/15)  30 OL (16/14) | *BCL2* | Bcl-2 | IHC | Positivity for Bcl-2 was seen in 26.7% of OL cases and among 30.0% of OSCC cases. No statistically significant differences between the expression of Bcl-2 in OL and OSCC were observed (*p* > 0.05), although increased expression of Bcl-2 in OSCC compared to OL may be an evidence of disease progression. | Bcl-2 gene product role is less clear in terms of progression of carcinogenesis since the interaction among members of the Bcl-2 protein family may control the sensitivity or resistance of cells to apoptosis. |
| Kannan et al., 1994  India | Cross-sectional | 30 OSCC  60 OL  10 Normal | *CDKN1A*  *EGFR* | p21  EGFR | IHC | Normal keratinizing mucosa showed mild p21 and moderate EGFR staining, while the non-keratinizing mucosa was p21-negative and mild EGFR positive. 67% of non-dysplastic OL, 80% of dysplastic OL and 69% of OSCC showed p21 positive staining. 77% of non-dysplastic OL, 70% of dysplastic OL and 88% of OSCC showed positive EGFR staining. | The study showed inter-group variations in the expression of p21 and EGFR and suggests their potential use as markers of transformation. Evaluation of these proteins may give answers in terms of diagnostic utility. |
| Khan et al., 2009  India | Cross-sectional | 29 OSCC  9 OL  7 Normal | *BIRC5 TP53* | Survivin  p53 | IHC  RT-qPCR | 72% of OSCC revealed survivin expression, which was significantly higher than normal oral tissues (0%) (*p<* 0.0008). 44% OL were survivin positive. 59% of OSCC revealed p53 expression, while 33% of OL and 14% of normal tissues were p53 positive. | High percentage of survivin and p53 overexpression in OL suggests early involvement in OSCC development. |
| Kitamura et al., 2012  Japan | Cross-sectional | 105 OSCC (53/52)  108 OL (68/40)  10 Normal | *KRT17*  *KRT13* | Keratin17  Keratin13 | IHC | CK17-positive cases: Normal - 0%; hyperplastic OL - 48.6%; dysplastic OL - 54.5%; OSCC - 96.2%. CK13-positive cases: Normal - 100%; hyperplastic OL – 70.3 %; dysplastic OL – 32.4 %; OSCC - 2.9%. CK17-positive expression gradually increased, while CK13-positive expression gradually decreased according to progression. | CK17 expression might be associated with OSCC differentiation and malignancy. A combination pattern of CK17/CK13 could be an appropriate biomarker of malignant transformation. |
| Klein et al., 2020  Brazil | Cross-sectional | 19 OSCC (13/6)  61 OL (35/26)  17 inflammatory hyperplasia (IH)  9 Normal | *MKI67*  *BMI1* | Ki-67  BMI-1 | IHC | There was a gradual increase in Ki-67 expression through normal (10.2%), IH (29.4%), non-dysplastic OL (33.1%), dysplastic OL (36.3%) and OSCC (62.8%) (*p*<0.01). Increasing positivity was observed for BMI-1 through normal (1.7%), IH (2.1%), non-dysplastic OL (2.4%), dysplastic OL (2.5%) and OSCC (2.8%). The means were statistically higher in OL compared to normal, and in OSCC, when compared to normal, IH and OL (p<0.01). | BMI-1 expression increases in early oral carcinogenesis and it may be associated with the occurrence of dysplastic changes. Furthermore, both Ki-67 and BMI-1 are directly correlated and  possibly play a role in initiation and progression of OSCC. |
| Kouketsu et al., 2019  Japan | Cohort | 106 OSCC  79 OL | *PDCD1*  *CD274* | PD-1  PD-L1 | IHC | PD-L1 and PD-1 expression tended to increase in association with malignant degrees of the oral epithelial lesions. PDL1 and PD-1 expression levels demonstrated significant differences between OL and OSCC specimens (*p* < 0.001). | The higher immunoreactivity of PD-L1 and PD-1 in OSCC than in OL suggests that the PD-L1/PD-1 pathway may be correlated with oncogenesis and tumor progression in the oral cavity. |
| Lameira et al., 2014  Brazil | Cross-sectional | 50 OSCC (38/12)  37 OL (21/16)  11 Mucocele | *MKI67*  *MCM3*  *CDKN1B* | Ki-67  MCM3  p27 | IHC | Ki-67 expression was higher in OSCC than in control (*p*<0.001) and mild dysplasia (*p*<0.01) groups, and there was a lower expression in control compared to moderate and severe dysplasia (*p*<0.05). p27 expression was lower in OSCC than in control, moderate and severe dysplasia (*p*<0.001). MCM3 expression was lower in control and mild dysplasia compared to severe dysplasia and OSCC (*p*<0.001). | MCM3 seems a better proliferation marker than Ki-67 in oral dysplastic lesions, as there was a significant increase in the expression of MCM3 in severe dysplasia compared to mild dysplasia samples. MCM3 may indicate that the cells are ready to proliferate. |
| Lin et al., 2010  Taiwan | Cohort | 204 OSCC (196/8)  28 OL | *HSPA5* | GRP78 | Western Blotting  IHC | The expression of GRP78 increased according to the malignant potential of oral lesions, with 14% in OL and 74% in patients with OSCC (*p*<.0001). Patients with GRP78 hyperexpression had a 4.9-fold increased risk of developing malignancy at the same location (*p*<0.01) | GRP78 hyperexpression is correlated with  increasing malignant potential of oral lesions, suggesting this molecule participates in early steps of oral oncogenesis. |
| Lin et al., 2016  China | Cross-sectional | 60 OSCC (16/44)  45 OL (18/27)  10 Normal | *IL37* | IL-37 | IHC | OL and OSCC lesions show higher IL-37 expression than normal tissue (*p*<0.001). IL-37 expression was detected in OL patients with mild and moderate dysplasia, but also in patients without dysplasia, although much weaker (*p*<0.001). IL-37 expression is associated with lymph node status (*p* = 0.006). | The results indicate IL-37 as a possible diagnostic and prognostic marker of malignant transformation on the oral mucosa. |
| Liu et al., 2017  China | Cross-sectional | 86 HNSCC (47/39)  40 OL (20/20)  86 Normal | *GPRC5A* | GPRC5A | IHC | The mean score of GPRC5A was high in normal tissues (141.22 ± 66.975), whereas it was significantly lower in OL (76.00 ± 67.389), and greatly repressed in HNSCC (34.63 ± 41.389). GPRC5A expression was gradually suppressed during oncogenesis of HNSCC (*p* < 0.01). | Repression of GPRC5A correlated with activation of STAT3 contributes to the oncogenesis of HNSCC. Thus, suppression of GPRC5A may serve as a molecular biomarker for the oncogenesis of HNSCC. |
| Logeswari et al., 2014  India | Cross-sectional | 50 OSCC  32 OL  10 Normal | *PDPN* | Podoplanin | IHC | All control samples were podoplanin-negative. 59.4% of OL cases and 82% of OSCC samples showed podoplanin expression in the epithelium. The expression of podoplanin among three groups was statistically significant (*p* = 0.00). | Podoplanin has a valuable role as a prediction marker for assessment of malignant transformation of OL and prognosis of OSCC. |
| Lopes et al., 2018  Brazil | Cross-sectional | 15 OSCC (10/5)  26 OL (12/14)  10 Normal | *CDH1*  *SNAI1* | E-cadherin  SNAIL | IHC | There was a significant increase in cytoplasmic E-cadherin expression in OSCC compared to normal mucosa (*p*<0.0001), OL (*p*<0.01) and dysplastic OL (*p*< 0.0001). Also, significant increase in nuclear SNAIL expression from normal mucosa to dysplastic OL (*p*<0.05) followed by decreased nuclear expression and increased cytoplasmic expression in OSCC (*p*< 0.05). | Increased nuclear SNAIL expression may be characteristic of dysplastic OL, and their role should be better investigated in OSCC. Cytoplasmatic expression of E-cadherin may be a marker of malignant transformation of OL into OSCC. |
| Madan et al., 2015  India | Cross-sectional | 15 OSCC  30 OL  5 Normal | *PCNA* | PCNA | IHC | The mean±SD PCNA LI in normal, non-dysplastic OL, dysplastic OL and OSCC was 25.73±2.80%, 40.56±8.55%, 44.95±16.31% and 80.35±12.37%, respectively. There was a significant difference between normal and dysplasia (*p*=0.020), normal and OSCC, dysplasia and OSCC and non-dysplastic OL and OSCC (*p*< 0.001). | PCNA can differentiate between normal and OSCC but it does not seem a good marker in delineating non-dysplastic OL from dysplasia. Thus, PCNA absolute reliability is still questionable. |
| Mao et al., 2020  China | Cross-sectional | 30 OSCC (27/3)  47 OL (44/3)  11 Normal | *BHLHE40* | DEC1 | IHC | The expression (mean ± SEM) of DEC1 in the OL group (15.14 ± 1.930) was significantly higher than in the normal group (0.1648 ± 0.09857) but was lower than that in the OSCC group (23.52 ± 3.051) (*p* < 0.0001). In the OL group there was a gradual increase from hyperkeratosis without dysplasia (8.561 ± 1.935), to mild dysplasia (16.97 ± 2.906) to severe dysplasia (17.43 ± 4.140) (*p=* 0.0628). | DEC1 protein expression sequentially increased in normal mucosa, OL and OSCC, and thus this protein may be used as a novel biomarker to detect the risk of malignant transformation from  OL to OSCC. |
| Markopoulos et al., 2009  Greece | Cross-sectional | 30 OSCC  10 OL  10 Normal (5/5) | *HSPBP1* | HSP70 | IHC | HSP70 expression was found to increase significantly  from normal oral mucosa to dysplastic OL (*p*= 0.000), and from dysplastic OL to OSCC (*p*= 0.000). | The expression of HSP70 in OSCC and in dysplastic OL points to HSP70 immunoreactivity as a marker of oral malignant potential. |
| Matsubara et al., 2011  Japan | Cross-sectional | 81 OSCC (58/23)  112 OL (72/40)  10 Normal | *TP63*  *MKI67*  *KRT14* | p63  Ki-67  Keratin 14 | IHC | The LIs of p63, Ki-67 and CK14 in the OL were significantly increased with the severity of epithelial dysplasia (*p*<0.0001). The p63-LI of the OL with malignant transformation was significantly higher than in OL with no malignant transformation (*p*<0.01). In OSCC patients, p63, Ki-67, and CK14 were overexpressed, compared to normal (*p*<0.0001). | These results suggested that increased p63 expression is a useful marker indicative of the degree of epithelial dysplasia and predictive of malignant transformation. |
| Matta et al., 2007  India | Cross-sectional | 120 OSCC (95/25)  89 OL (79/10)  66 Normal | *YWHAZ* | 14-3-3zeta | IHC  Immunoblot | Significant increase in 14-3-3ζ expression was observed in different stages of oral tumorigenesis (normal to hyperplasia, dysplasia and OSCC, *p*< 0.001). Increased expression of 14-3-3ζ was observed by immunoblotting in OL and OSCCs as compared to normal oral tissues. | Increased expression of 14-3-3ζ occurs in oral hyperplasia and across all the carcinogenic pathway. 14-3-3ζ is involved in cell signaling pathways of inflammation, cell proliferation and abrogation of apoptosis during oral carcinogenesis. |
| Matta et al., 2009  Canada/ India | Cross-sectional | 100 OSCC (75/25)  199 OL (159/40)  55 Normal | *HNRNPK* | hnRNP K | IHC | 51 normal cases (93%) showed weak or undetectable  hnRNP K immunostaining. 141 OL (71%) and 78 OSCC samples (78%) showed significant increase in nuclear hnRNP K immunostaining. Intense hnRNP K staining was also observed in the cytoplasm of 38% of OSCCs. Significant increase in nuclear hnRNP K staining was observed in different stages of tumorigenesis (normal, OL and OSCC) (*p*< 0.001). | It was observed that hnRNP K is expressed in early stages of oral lesions, even before onset of dysplasia and in tumors. Its predominant nuclear localization in OL, but increased cytoplasmic expression in OSCC, suggests that nuclear-cytoplasmic translocation may have an important role in malignant transformation of oral cancer. |
| Meng et al., 2011  China | Cross-sectional | 68 OSCC (42/26)  45 OL  25 Normal | *TGFBR1*  *TGFBR2*  *TGFBR3* | T𝛽RI  T𝛽RII  T𝛽RIII | IHC | Homogeneous, moderate, and intense cytoplasmic expression of T𝛽RI was observed in OSCC, OL and normal tissues. T𝛽RII and T𝛽RIII expression gradually decreased according to the progression of carcinogenesis (from normal specimens through OL to OSCC). | The loss of the T𝛽RII and T𝛽RIII expression in oral epithelium is a common event in OSCC patients. Therefore, we strongly believed that T𝛽RII and/or T𝛽RIII may be used as predictors of tumorigenesis and severity. |
| Moraes et al., 2019  Brazil | Cross-sectional | 72 OSCC (65/7)  32 OL (17/15)  10 Normal | *BDNF*  *NTRK2*  *AKT1*  *RPS6*  *BMI1* | BDNF  TrkB  Akt  RPS6  BMI-1 | TMA  IHC | Normal mucosa and OL showed similar expression of BDNF, TrkB, p‐Akt, and p‐RPS6 but differed on p‐TrkB expression. OL had a higher score compared to normal mucosa. Also, TrkB expression was increased in dysplastic OL compared to non‐dysplastic OL. OSCC presented increased expression of BNDF, TrkB, and p‐Akt compared to normal mucosa and OL. p‐RPS6 expression was decreased in OSCC compared to normal group and OL. | The results demonstrate that BDNF/TrkB/Akt signaling pathway is more expressed in OSCC compared to OL and normal mucosa. The increased availability of BDNF influences OSCC prognosis and shorter survival time presumably by activating downstream targets such as Akt. |
| Nakabayashi et al., 2014  Japan | Cross-sectional | 97 OSCC  106 OL  26 Normal | *PITX1* | PITX1 | IHC | The PITX1 LI was 72.8±6.5, 52.3±9.24 and 4.8±4.25 (mean±SD) in the normal oral mucosa, OL and OSCC samples, respectively. PITX1 expression levels were significantly decreased in OL compared with the normal oral mucosa (*p*<0.001), and were further decreased in OSCC (*p*<0.001). | PITX1 is considered a candidate tumor suppressor gene and its downregulation in oral epithelial cells may be involved in OSCC carcinogenesis. Also, PITX1 may serve as a novel biomarker for predicting prognosis in OL. |
| Nanda et al., 2012  India | Cross-sectional | 10 OSCC (7/3)  10 OL (9/1)  10 Normal (6/4) | *KRT8*  *KRT18* | Keratin 8  Keratin 18 | IHC | Expression of CK8 was seen in 20% of the OL and in 30% of OSCC. None of the normal mucosa stained for CK8 (*p =* 0.34). CK18 positivity was seen in 40% of OL, 50% of OSCC and in none of the normal mucosa (*p*= 0.08). | Alteration on CK8 and CK18 expression may represent an early event in the pathogenesis of OSCC. Thus, CK8 and CK18 may have potential use as markers of malignant transformation. |
| Narashiman et al., 2014  India | Cross-sectional | 20 OSCC (15/5)  15 OL (13/2)  5 Normal | *MUC4* | Mucin-4 | IHC | 7/15 cases of OL stained positive for MUC4. 9/10 cases of WDOSCC and 5/10 MDOSCC showed MUC4 positivity. The staining pattern among the study groups was statistically significant (*p* = 0.010). | MUC4 expression progressively increased from OL to OSCC, especially among the well-differentiated. MUC 4 plays a vital role in the pathogenesis of OSCC and may be a useful marker for OL and OSCC. |
| Nasser et al., 2011  Germany | Cohort | 8 OSCC  48 OL | *TP53*  *CDKN2A*  *MKI67*  *CCNDBP1*  *RB1* | p53  p16^INK4a^  Ki-67  Cyclin D1  pRb | IHC | Overexpression of Ki-67, p53 and Cyclin D1, and loss of expression of p16^INK4a^ linearly increased from the nondysplastic to dysplastic OL and to OSCC. Combined aberration in p53 ⁄ p16^INK4a^ and in p53 ⁄ p16^INK4a^ ⁄ Ki-67 also increased from the nondysplastic OL to OSCC. pRb remained normal. | Nondysplastic OL with dual p53⁄ p16^INK4a^ and triple p53 ⁄ p16^INK4a^ ⁄ Ki-67 aberration are at risk of progression, with promising positive predictive  value. |
| Negi et al., 2015  India | Cross-sectional | 15 OSCC  15 OL  15 Normal | *BIRC5* | Survivin | IHC | 20% of normal mucosa samples were survivin positive, while 53.33 of OL and 80% of OSCC cases were positive for survivin staining. Statistically significant difference on survivin positivity was observed among the three groups (𝑝<0.001). | The study demonstrated a high expression of survivin in OL and OSCC cases, which may occur as an early phenomenon in the beginning and progression of OSCC. |
| Nguyen et al., 2017  Japan | Cohort | 99 OSCC  93 OL  6 Normal | *LAMC2* | LAMC2 | IHC  cDNA Microarray | The presence of LAMC2-positive foci is a significant predictive factor with an approximately 11-fold increased risk of malignancy (*p* = 0.002). Cases of LAMC2-positive OL had an approximately 14-fold risk of malignant progression compared to the LAMC2-negative OL cases (*p* = 0.003). | LAMC2 is a protein directly linked to invasion, which discriminates dysplasia from cancer. LAMC2 evaluation may be of benefit to the diagnostic practice and contribute to a more objective and precise assessment of OL. |
| Nogami et al., 2003  Japan | Cross-sectional | 7 OSCC (2/5)  6 OL (3/3)  5 Normal (2/3) | *MKI67 TP53*  *BCL2*  *BAX* | Ki-67  p53  Bcl-2  Bax | IHC  TUNEL | Positive expression for Ki-67 was 4.7± 1.8% in normal mucosa, 6.4 ± 3.3% in OL, and 14.9±4.9% in OSCC (*p* < 0.05). All normal cases were p53 and Bcl-2 negative, a few positive cells were detected on OL and many positive cells were seen in OSCC. Control cells were weakly positive for Bax, OL were moderate to strong and OSCC were again weak. | The expression of p53, Ki-67 and Bcl-2 increased in direct relation to the grade of potential malignancy, whereas the opposite was observed for Bax and apoptotic cells. |
| Ohkura et al., 2005  Japan | Cross-sectional | 21 OSCC (14/7)  11 OL (6/5) | *KRT4*  *KRT13*  *KRT14*  *KRT17*  *TGM3* | Keratin 4  Keratin 13  Keratin 14  Keratin 17  Transglutaminase-3 | Differential display  Northern blotting  RT-qPCR  IHC | The expression of KRT4, KRT13 and TGM3 is significantly higher in hyperplastic and in mild to moderately dysplastic OL than in severe dysplasia and OSCC (*p* < 0.05). The levels of KRT-14 and KRT-17 were significantly lower in hyperplasia and in mild to moderate dysplasia than in severe dysplastic tissue and OSCC (*p* < 0.007 and *p* < 0.001). | The expression of the keratin and transglutaminase genes may provide an index of the growth, differentiation and/or histological features of oral malignancies. |
| Palani et al., 2011  India | Cross-sectional | 30 OSCC (16/14)  15 OL (13/2)  10 Normal (8/2) | *hTERT* | Telomerase reverse transcriptase | IHC | There was statistically significant difference between cellular localization of hTERT stain between OSCC and OL (*p*=0.020) and OSCC and normal (*p*=0.006). The mean LI increased from normal (28.3±12.3), to OL (44.06±14.6), to OSCC (47.56±21.30) (*p*=0.00) | It was observed increased expression of hTERT protein in OSCC and OL samples compared to normal mucosa. This finding must be explored to establish hTERT as a potential marker of malignant transformation. |
| Pande et al., 1998  India | Cross-sectional | 35 OSCC (31/4)  22 OL (18/4)  30 Normal | *RB1 CDKN2A* | pRb  p16 | IHC | 37% of OSCC, 41% of OL and 93% of normal oral mucosa exhibited p16 positive immunostaining. pRb positive staining was observed in 34.3% of OSCC cases, 36% of OL and in 93% of normal oral mucosa. There is strong correlation between the expression of p16 and pRb proteins in OSCCs (*p*=0.007) and in OL (*p*=0.0052). | Alterations of p16/pRb pathway may be involved in betel- and tobacco-related oral tumorigenesis. New biomarkers could aid in identifying patients with OL with higher risk of malignant transformation and could be preferred for chemoprevention and intervention strategies. |
| Patil et al., 2015  India | Cross-sectional | 40 OSCC  40 OL | *PDPN* | Podoplanin | IHC | There was an increase of the podoplanin expression scores from mild (1.32 ± 0.43) to severe dysplasia (3.03 ± 0.33), as well as from well (1.03 ± 0.74) to poorly differentiated (7.07 ± 0.86) OSCC (*p* < 0.01). | Podoplanin can be used as a biomarker for early oral tumorigenesis and for malignant transformation risk assessment of potentially malignant lesions and as a biomarker for advanced grades of OSCC. |
| Pontes et al., 2009  Brazil | Cross-sectional | 15 OSCC  30 OL  10 Normal  (33/22) | *MT2A AKT1* | Metallothionein  p-Akt | IHC | All samples were p-Akt and MT positive with statistically significant difference among the groups (*p*<0.0001). There was a significant increase in p-Akt and MT positivity comparing normal oral mucosa to severe dysplasia and OSCC (*p*<0.01). A significant correlation between p-Akt and MT immunoexpression was observed (*p*<0.0001). | p-Akt and MT may play an important role in the transformation of a potentially malignant oral lesion to OSCC. Also, high expression of p-Akt and MT in OSCC suggests that these proteins may be accurate targets for therapeutic approaches. |
| Pontes et al., 2013  Brazil | Cross-sectional | 15 OSCC (9/6)  28 OL (14/6)  6 Normal (3/3) | *NFKB1 PTGS2* | NF-κB COX-2 | IHC | There was a gradual increase in NF-κB expression among the groups, with statistically significant difference in the OSCC group compared with the others (*p<*0.05). COX-2 expression increased as the severity of lesion progressed from normal to invasive cancer, with a statistically significant difference between moderate dysplasia and control (*p* = 0.0163). | NF-κB may participate in the malignant phenotype acquisition process of OSCC in its late stages, whereas COX-2 may be involved in the early stages. Both proteins might be valid targets for therapeutic approaches in patients with OSCC and potentially malignant oral lesions. |
| Poomsawat et al., 2010  Thailand | Cross-sectional | 15 OSCC (7/6)  34 OL (18/16)  12 Normal (6/6) | *CDK4*  *CDK6* | Cyclin-dependent kinase 4  Cyclin-dependent kinase 6 | IHC | The number of cdk4-positive cases was significantly lower in normal mucosa than in dysplastic OL (*p*=0.0008) and OSCC (*p*=0.0213). The percentages of cdk4-positive cells in the normal mucosa group was significantly lower than in OSCC (*p*<0.05) and dysplastic OL (*p*< 0.05). The highest cdk6 expression was 60% in OSCC, statistically different from normal mucosa samples, which were 8.33% stained (*p*= 0.0140). | Overexpression of cdk4 and cdk6 were observed in OSCC, indicating that these proteins seems to play a crucial role in this cancer. However, aberrant expression of only cdk4 was found in OL with mild dysplasia. cdk4 may be involved in early stages of carcinogenesis of OSCC. |
| Poomsawat et al., 2014  Thailand | Cross-sectional | 41 OSCC (24/17)  63 OL (35/28)  10 Normal (4/6) | *BIRC5*  *CASP3* | Survivin  Caspase-3 | IHC | Survivin expression was significantly higher in the OSCC group than in the normal mucosa (*p*<0.0001), non-dysplastic and dysplastic OL (*p*<0.01). Normal mucosa showed a low rate of cytoplasmic survivin expression compared with non-dysplastic and dysplastic OL (*p*<0.01). Caspase 3 expression in OSCC was significantly higher than those of normal mucosa, non-dysplastic and dysplastic OL (*p*<0.01). | Survivin and caspase 3 seem to play a crucial role in oral carcinogenesis. The cytoplasmic and nuclear staining in the same cells of survivin or caspase 3 is common in OSCC, suggesting that this expression pattern may be a useful tool for treatment plan of premalignant lesions. |
| Priyanka et al., 2019  India | Cross-sectional | 15 OSCC  15 OL  10 Normal | *HSPBP1* | HSP70 | IHC | Weak cytoplasmic HSP70 staining in normal oral mucosa was observed. In OL, as the grade of dysplasia increased, the intensity and/or distribution of the staining progressively increased, suggesting a positive association between HSP70 expression and severity of dysplastic lesions (*p*= 0.87). In OSCC, there was a significant difference in the expression of HSP70 according to different histological grades (*p*< 0.01). Mean IID scores of normal and OL were 0.5% and 4% (*p* < 0.05). Mean IID scores of normal and OSCC were 0.5% and 9.73% (*p* < 0.01). Mean IID scores of OL and OSCC were 3.56%, 4.23% (*p* < 0.01). | HSP70 expression was significantly higher in OL and OSCC groups than in the control group. Significant increased expression of HSP70 was observed from control to OL and OSCC cases, supporting the critical role of HSP70 in the development of OSCC and also increased HSP70 expression is critical for tumor growth and might be important potential biomarker for evaluating the role in treatment and prognosis. |
| Ralhan et al., 1997  India | Cross-sectional | 81 OSCC (56/25)  32 OL (24/8)  41 Normal | *ABCB1* | Phospho-glycoprotein (P-gp) | IHC  Western Blotting | 100% of normal mucosa adjacent to cancerous lesions and none of normal mucosa from a contralateral site were P-gp positive. 50% of OL cases, 84% of primary OSCC and 100% of recurrent OSCC showed P-gp immunostaining. A significant increase in P-gp immunopositivity across normal, OL, primary OSCC and recurrent OSCC groups (*p*<0.01). A significant increase in P-gp positivity was observed in recurrent OSCCs compared to primary tumours (*p=*0.019). | Expression of P-gp proportionately increased according to severity of dysplasia. Also, markedly increased levels in primary OSCC and significantly higher levels in recurrent OSCCs were observed, indicating that alteration in P-gp expression may be an early event in oral oncogenesis. |
| Rathee et al., 2021  India | Cross-sectional | 24 OSCC (14/10)  24 OL (23/1)  12 Normal | *MUC1*  *MUC4* | Mucin-1  Mucin-4 | IHC | Normal oral mucosa, OL and OSCC groups showed positive immunoexpression in 3/12, 12/24 and 20/24 cases respectively for MUC1. Considering MUC4, normal oral mucosa, OL and OSCC showed positive immunoexpression in 4/12, 11/24 and 22/24 cases respectively. Higher frequency for positive expression in OSCC, while higher negative expression was seen in OL and normal groups with statistically high significant difference among groups (p = 0.002 (MUC1) and 0.000 (MUC4)). | MUC1 and MUC4 could act as markers of malignant progression given their rise in immunoexpression from normal mucosa to OL to OSCC. MUC1 and MUC4 could be a worthwhile prognostic marker in OSCC and their coexpression might be involved in the pathogenesis and disease progression of OSCC. |
| Ravi et al., 1996  India | Cross-sectional | 46 OSCC  42 OL | *TP53*  *BCL2* | p53  Bcl-2 | IHC | All OSCC tissues expressed p53 and Bcl-2, with the majority showing 60-90% positivity. All dysplastic OL showed expression of p53 and Bcl-2. 8 of 27 hyperplastic OL showed more than 10% p53 positive cells and none had more than 3% Bcl-2 positive cells. | The expression of p53 and bcl-2, genes with opposite but inter-related functions, may be critical in the process of carcinogenesis and tumor progression. |
| Renkonen et al., 2002  Finland | Cross-sectional | 73 OSCC  64 OL  38 Normal | *PTGS2* | COX-2 | IHC | Cox-2 expression increased gradually from normal epithelium (0.97) to hyperplasia (1.58), dysplasia (2.04) and OSCC (2.42). The difference between Cox-2 expression in the normal and hyperplasia, dysplasia and OSCC was statistically significant (*p*=0.0041, *p*=0.0011, and *p=*0.0001 respectively). | OSCC and precursor lesions express Cox-2 in progressive manner toward more malignant lesions, suggesting a role for Cox-2 in the carcinogenesis of these tumors. |
| Ries et al., 2021  Germany | Cross-sectional | 45 OSCC  99 OL  20 Normal | *PDCD1*  *CD274* | PD-1  PD-L1 | RT-qPCR  IHC | High PD-1 expression was detected in the epithelial (p = 0.001) and subepithelial layers (p = 0.005) of transformed OL (T-OL) compared to non-transformed OL (N-OL). The overexpression in both tissue layers was significantly associated to malignant transformation (pE = 0.0001, pS = 0.02). In T-OL a significant 60-fold overexpression (p = 0.04) and in OSCC a 99.4-fold increase of PD-L1 was detected. Overexpression was related to malignant transformation (p = 0.03) and malignancy (p = 0.003). | Upregulation of PD-L1 may be associated with disease progress in OL and OSCC. A high degree of inflammation in OL was associated with high expression of PD-L1, PD-1 and malignant transformation. PD-1/PD-L1 may represent a prognostic indicator to determine the risk of malignant progression of OL. Local epithelial immunosuppression may be induced by PD-L1 expression and trigger malignant transformation. |
| Routray et al., 2013  India | Cross-sectional | 40 OSCC (20/20)  40 OL (32/8)  20 Normal | *SPP1* | Osteopontin | IHC | 55% of mild dysplasia cases, 0% of moderately dysplastic cases and 80% of severely dysplastic OL were OPN positive with no statistically significant difference compared to normal epithelium. OPN intensity increased significantly with loss in degree of differentiation of tumor cells. There was a statistically significant difference between normal epithelium (0.6 ± 0.51) and OL (0.7 ± 0.73), with OSCC (1.45 ± 1.05). | The results indicate that OPN expression is not a relevant diagnostic and prognostic marker for oral mucosal dysplasia and malignant transformation, although OPN certainly plays a role as prognostic marker in OSCC. |
| Sabitha et al., 2018  India | Cross-sectional | 90 OSCC  90 OL  30 Normal | *TP53*  *BAX*  *hTERT* | p53  Bax  Telomerase reverse transcriptase | IHC | p53 expression between normal epithelium, OL and OSCC was statistically significant (*p*<0.001). There was a statistically significant expression of BAX between normal mucosa and OL and OSCC (*p*<0.05). Also, significant differences among normal epithelium, OL and OSCC in hTERT expression (*p*<0.005). | It was observed a significantly altered expression of p53, Bax and hTERT in histological grades of dysplastic OL and OSCC. The significant expression of these markers suggests their role as possible markers of malignant transformation. |
| Saileela et al., 2018  India/ Malaysia | Cross-sectional | 12 OSCC  8 OL  9 Normal | *HSPB1*  *(HSP27)* | Heat shock protein beta-1 | IHC | Comparing all samples, significant difference in intensity and percentage of positively stained cells was observed (*p*=0.001). The percentage and intensity of positively stained cells were significantly higher in OSCC than in normal mucosa (*p*=0.002), but no significant difference was observed between dysplasia and normal tissues (*p*=0.504). | HSP27 may be used as a prognostic marker of OSCC but further studies with larger sample size may be necessary to obtain a correlation between oral potentially malignant disorders and normal tissues. |
| Sakthivel et al., 2020  India | Cross-sectional | 10 OSCC  10 OL  5 Normal | *BIRC5* | Survivin | IHC | The expression of survivin was positive in 70% of OSCC and in 50% of OL samples. All samples of normal oral mucosa were negative for the expression of survivin. | The up-regulation of survivin in OL and OSCC demonstrates its role in carcinogenesis and indicates that survivin may be potentially used as a reliable marker to identify the high-risk group for malignant transformation. |
| Santhi et al., 2006  India | Cross-sectional | 54 OSCC  54 OL  40 Normal | *NFKB1*  *PTGS2* | NF-kB (p50, p65 and IκB)  COX-2 | IHC  EMSA  ELISA  RT-qPCR | The intensity of p50 expression increased with histological progression (*p*<0.001). The immunoreactivity of NF-κB inhibitor protein IκB-α also showed a significant association with worsening histology (*p*<0.001). OSCC tissues expressed significantly more COX-2 than OL. COX-2 expression was very low in normal oral tissues | The samples had an increase in the expression and translocation of NF-κB and  increase in the expression of COX-2, suggesting the validation of NF-κB and COX-2 as participants of oral  carcinogenesis. |
| Sawant et al., 2018  India/ Norway/ USA | Cross-sectional | 213 OSCC  68 OL  34 Normal | *DSP*  *JUP*  *DSG2* | Desmoplakin Plakoglobin Desmoglein 2 | IHC | The staining intensity of desmoplakin, plakoglobin, and desmoglein 2 were high in hyperplastic epithelium, and progressively decreased in the subsequent stages of the disease, with significant difference from normal oral epithelium (*p*<0.05). Low expression of desmoplakin (*p*=0.022) and plakoglobin (*p*=0.011) was associated with lower survival in patients with OSCC. | Alterations in desmosomal proteins are one of the important and main steps during the process of human oral tumorigenesis. These alterations may be useful indicators of early identification of high-risk potentially malignant lesions. |
| Servato et al., 2019  Brazil | Cross-sectional | 100 OSCC (64/21)  62 OL  22 Normal | *NOS2* | iNOS | RT-qPCR  IHC | The lower NOS2 relative quantitative expression was found in normal mucosa, and progressively increased to intermediate and high levels in the OL and OSCC groups, respectively (*p* = 0.0003). It was observed a significant difference in the iNOS immune staining between the normal and OSCC groups (*p* = .0280). | According to the results, it can be concluded that iNOS plays a vital role in the development and progression of human oral dysplasias and neoplasias. |
| Shyam et al., 2014  India/ USA | Cross-sectional | 30 OSCC  15 OL  7 Normal | *CDK4* | Cyclin-dependent kinase 4 | IHC | Expression of nuclear and cytoplasmatic CDK4 gradually increased from mild to severe dysplasias and from well to poorly differentiated OSCC (*p=*0.001). However, CDK4 staining intensity decreased from well to poorly differentiated OSCCs and increased from mild to severe dysplasia. | The immunoreactivity and the staining intensity progressively increased from normal to OL and OSCC. Further studies are still needed to understand the CDK4 function in the process of oral carcinogenesis. |
| Singh et al., 2019  India | Cross-sectional | 30 OSCC (16/14)  26 OL (18/8)  10 Normal | *NES* | Nestin | IHC  Western Blotting | 50% of normal samples were nestin negative and 50% presented mild staining. 35% of OL samples were nestin negative, 42% presented mild staining and 23% were moderately stained. Statistically significant difference among the OL groups was found (*p*<0.05). Among the 30 cases of OSCC, 2 cases (7%) showed moderate expression, 10 cases (33%) showed mild expression, and the remaining 18 cases (60%) failed to express nestin. | Nestin plays a brief but pivotal role in oral carci-nogenesis and may be a valuable marker for neo-angiogenesis in OSCC. Nestin expression in OL samples suggests that it could be an early event in the multistage carcinogenesis of oral epithelium. |
| Singh et al., 2020  India and Kingdom of Saudi Arabia | Cross-sectional | 30 OSCC (18/12)  30 OL (22/8)  30 Normal (23/7) | *NOS2* | iNOS | IHC | A significantly higher percentage (83.3%) of patients with OSCC showed iNOS expression compared to patients with OL (73.3%) (p < 0.001). None of the control samples showed iNOS expression. A significant increase in the number of cases with iNOS expression was seen along with the deteriorating grades of dysplasia, and in advancing histopathological grades and clinical TNM stages (p < 0.01). | A proportional increase was observed in iNOS expression as the clinical stages and grades progressed in OL group. Similarly, in OSCC 83.3% of the cases have shown significantly high levels of iNOS expression compared to OL and normal epithelium. iNOS may be considered a candidate for OL early diagnostic marker and OSCC prognostic marker. |
| Singla et al., 2018  India | Cross-sectional | 40 OSCC (32/8)  40 OL (33/7)  40 normal | *TP53*  *EGFR*  *ERBB2* | p53  EGFR  c-erbB2 | IHC | The expression of p53 and EGFR were correlated between OL, OSCC, and control (*p* ≤ 0.001), which means the expression of both proteins increased with progression toward malignancy. C‑erbB2 was found to be negative. | Both p53 and EGFR play an important role in identifying OL which may progress to OSCC. |
| Soares et al., 2006  Brazil | Cross-sectional | 28 OSCC  40 OL  27 Normal | *PCNA*  *TP53*  *MKI67* | PCNA  p53  Ki-67 | IHC | p53 expression increased progressively from normal, to non-dysplastic OL, to dysplastic OL and OSCC (*p*<0.05). No significant difference in PCNA expression was observed among the groups. Ki-67 was more efficient than PCNA in indicating progressive proliferation among the groups, but with no difference between normal and OL (*p*>0.05). | The overexpression and immunostaining patterns of p53, and increased cell proliferation detected by Ki-67, could be used to predict malignant transformation. However, PCNA does not seem to be useful for this purpose. |
| Soni et al., 2005  India | Cross-sectional | 220 OSCC (167/53)  90 OL (72/18)  81 normal | *CCNDBP1*  *CDKN2A*  *RB1* | Cyclin D1  p16  pRb | IHC | Cyclin D1 expression in OSCCs was significantly higher than in OL and normal tissues (*p*=0.013). A significant loss of pRb was observed in transition from hyperplasia to dysplasia (*p*=0.005). p16 positivity was detected in 45% normal tissues, 41% of OL and in 40% of OSCCs. Transition of OL to OSCC was associated with loss of pRb and overexpression of cyclin D1 (OR = 2.938, *p*=0.001). | Dysplastic lesions harboring concomitant loss of pRb and increased cyclin D1 expression may be at high risk for transition to malignancy. |
| Sravya et al., 2016  India | Cross-sectional | 30 OSCC  30 OL  30 Normal | *BUB1B* | BUBR1 | IHC | The mean±SD of BUBR1 expression among normal, dysplasia and OSCC groups were 0.00±0.00, 2.13±1.63 and 2.67±1.57, respectively (*p*=0.00001). It was observed a significant increase on BUBR1 staining intensity scores according to histological grades of dysplasia and OSCC (*p*=0.00001). | Statistical significance in the expression of BUBR1 was observed in normal subjects, patients with OL and OSCC. Higher IHC scores were detected according to increasing grades of dysplasia and OSCC, suggesting its role as a prognostic indicator. |
| Srinivasan & Jewell, 2001  India/ USA | Cross-sectional | OSCC  17 OL (16/1)  10 Normal | *TGFA EGFR* | TGF-α EGFR | Quantitative IHC | The area and intensity of staining of TGF-α and EGFR increased significantly in oral epithelium, stratum germinativum and in the stratum spinosum of OL and OSCC compared to normal mucosa (*p*< 0.05). | Altered expression of TGF-α in the basal and parabasal cells may provide an early marker for the onset of epithelial dysplasia preceding OSCC. |
| Sudha & Hemavathy, 2011  India | Cross-sectional | 30 OSCC  10 OL | *BCL2* | Bcl-2 | IHC | 86.7% of OSCC cases showed positive Bcl-2 staining, mainly in the basal layer. All OL cases (100%) showed Bcl-2 positive staining, especially in the basal layer. It was observed a statistically significant difference on Bcl-2 positive topographic expression between OSCC and OL (*p=* 0.0139). | There was an increased Bcl-2 expression in oral potentially malignant disorders, such as OL, and OSCC. However, the role of bcl-2 during the development and progression of oral neoplasia, should be further investigated. |
| Sun et al., 2016  China | Cross-sectional | 21 OSCC (8/13)  44 OL (25/16) | *CD4*  *FOXP3*  *CD68*  *IL10*  *IL4*  *TGFB1*  *IFNG*  *CCL2* | CD4  Foxp3  CD68  IL-10  IL-4  TGF-𝛽1  IFN-𝛾  MCP-1 | IHC | CD4, Foxp3, CD68 and IL-10 expression increased gradually according to oral dysplasia, with statistically significant difference from OL to OSCC (*p*=0.0017, *p*=0.012, *p*<0.0001 and *p*<0.001, respectively). Significantly fewer IL-4+, TGF-𝛽1 and IFN-𝛾+ cells were seen in OSCC than in OL (*p*= 0.0001). No significant differences were seen in the number of MCP-1+ cells between OL and OSCC. | The study adds to what is known about how chronic inflammation can induce immune suppression and contribute to the occurrence of OSCC. |
| Sundar et al., 2021  India | Cross-sectional | 21 OSCC  21 OL | *POSTN* | Periostin | IHC | Among the 21 cases of OSCC, 19 cases (90.5%) showed positive periostin expression. Hyperkeratosis with mild dysplasia showed significantly higher periostin expression compared to moderate and severe dysplasia (p = 0.055). Periostin expression was significantly higher in OSCC compared with OL (p = 0.003) | The increased expression of periostin resulting from stromal and carcinoma cells may be a marker of aggressive behavior, as it correlates with poor prognosis. However, periostin is not a conceivable marker to predict the malignant transformation of OL. |
| Sutariya & Manjunatha, 2016  India/ Saudi Arabia | Cross-sectional | 30 OSCC  15 OL | *CDKN1A*  *BCL2* | p21  Bcl-2 | IHC | The Bcl-2 positivity was observed in all cases (100%) of OL and in 83.33% of OSCC cases. p21 positivity was seen in 80% of cases of OL and in 80% of OSCC cases. There was no statistical significance in the expression of p21 and Bcl-2 between OL and OSCC. | The expression of the p21 and Bcl-2 proteins in premalignant lesions and OSCC supports their role in early stages of oral carcinogenesis. |
| Tashiro et al., 2020  Japan | Cross-sectional | 132 OSCC (70/62)  45 OL (23/22) | *KRAS HRAS NRAS*  *BRAF*  *MAPK3 MAPK1*  *AKT1*  *MTOR*  *STAT3* | KRAS HRAS NRAS  BRAF  pERK1/2 pAkt pmTOR pSTAT3 | IHC | KRAS and NRAS expression was significantly decreased in OSCC compared to non-dysplastic OL, mild and severe dysplasia (*p*<0.001). pERK1/2 and pmTOR expression was significantly enhanced in severe dysplasia compared to non-dysplastic OL (*p*<0.05). pAkt and pmTOR expression was significantly enhanced in OSCC compared to non-dysplastic OL (*p*<0.01). | The expression levels of several growth factors including MAPK, Akt, and STAT3 were analyzed in OSCC and OLs. There were differences of expression in each signaling pathways in progressive stages of carcinogenesis, suggesting that each signaling pathway might play difference roles in the conversion of OLs into OSCC. |
| Thiem et al., 2017  Germany/ India | Cross-sectional | 36 OSCC  95 OL  (90/41) | *VEGFA* | VEGF-A | IHC | VEGF-A expression was significantly increased from OL through dysplasia (mild and moderate; all *p*< 0.05) to OSCC. However, no significant difference was found between non-dysplastic OL and mild dysplasia OL (*p* > 0.05). | The study supports the idea of upregulated VEGF-A expression in OL compared to other sites. It was observed increased VEGF-A expression in OL through dysplastic OL to OL-derived OSCC. |
| Thorup et al., 1998  Denmark/ Norway/ USA | Cross-sectional | 18 OSCC  21 OL  11 Normal | *ITGA2*  *ITGA3*  *ITGA6*  *LAMA3* | Integrin receptors  α2𝛽1, α3𝛽l and α6𝛽4 Laminin-  5 | Immunofluorescence | In OSCCs, there was patchy loss of α3𝛽l, α6𝛽4 and laminin-5 expression and a tendency to increased α2𝛽1 expression. Similarly, OLs and normal tissues showed alterations of the expression of α3𝛽l, α6𝛽4 and laminin-5. OLs presented loss of α2𝛽1 and α3𝛽l in the suprabasal cells. There was no unequivocal expression of the adhesion molecules distinguishing between inflammatory tissue, OL, and OSCC. | Integrin and laminin-5 expression may not be used as histologic markers of imminent malignancy in OL, because alterations found in normal tissues were similar to those in OSCCs and OL, which were all associated with inflammatory reactions in  the surrounding stroma. |
| Tripathi et al., 2010  India / Canada | Cross-sectional | 100 HNSCC (75/25)  166 OL  100 Normal | *S100A7* | S100-A7 | IHC  RT-qPCR  Western Blotting | There was significant increase in S100A7 expression in normal, hyperplasia, dysplasia and HNSCC tissues (*p<*0.001). Significant increase in nuclear S100A7 expression was observed in HNSCC compared to dysplasia (*p*= 0.005). It was also observed increased levels of S100A7 transcripts in hyperplasia, dysplasia, and HNSCC compared to normal tissues. | S100A7 was shown to be expressed in early stages of oral lesions, prior to onset of dysplasia and HNSCC. Nuclear S100A7 may be associated with increased transformation risk of oral premalignant  lesions and recurrence in HNSCC. |
| Turatti et al., 2005  Brazil | Cross-sectional | 24 OSCC  41 OL  15 Normal | *JUN*  *FOS*  *CCNDBP1* | c-Jun  c-Fos  Cyclin D1 | IHC | The number of c-Jun positive cells was significantly higher in mild dysplasia (*p*=0.031), moderate to severe dysplasia and OSCC(*p<*0.001), compared to normal mucosa. The expression of c-Fos in mild dysplasia was significantly lower than in moderate to severe dysplasia (*p*=0.018), normal mucosa and OSCC (*p=*0.001). Cyclin D1 expression in OSCC was significantly higher than in normal mucosa, mild dysplasia (*p*=0.014), and moderate to severe dysplasia (*p=*0.043). | c-Jun, c-Fos and cyclin D1 are altered in dysplastic epithelium and OSCC. Probably, the damage occurring in some of these proteins could influence other genes function, especially those involved in the cell cycle. Their participation on malignant transformation should be further elucidated. |
| Vadla et al., 2020  India | Cross-sectional | 30 OSCC  30 OL  30 Normal | *STMN1* | Stathmin | IHC | Statistically significant increased expression of stathmin was observed in OSCC group (2.50 ±1.33) compared to the OL group (2.11 ± 1.54) and the normal tissue (0.00) (p = 0.0001). A statistically significant difference was also observed between various histopathological grades of dysplasia and grades of OSCC with respect to IHC scores (p = 0.0001). | Statistical correlation between increased grades of the disease with expression levels of stathmin could be found, suggesting that stathmin expression contribute to disease progression and that stathmin might have a potential role as an early diagnostic biomarker. |
| Varghese et al., 2011  India | Cross-sectional | 15 OSCC  10 OL  5 Normal | *NOS2* | iNOS | IHC | None of the normal tissues were iNOS positive. 40% of OL cases were iNOS positive with nuclear and cytoplasmic staining in basal and supra basal cells. 10 of 15 OSCC cases (66,6%) were positively stained for iNOS. | The early expression of iNOS in potentially malignant lesions may suggests that it could possibly play a role in the transformation of normal epithelium to dysplasia and further to OSCC. |
| Varun et al., 2014  India | Cross-sectional | 20 OSCC (16/4)  20 OL (20/0)  10 Normal | *TP53*  *TP63* | p53  p63 | IHC | There was a significant increase in the mean p53 LI in OSCC compared to OL and normal mucosa (*p* < 0.01). There was a significant increase in the mean p63 LI of OSCC compared to OL (*p* < 0.05), and normal mucosa (*p* < 0.01). Mean LI of p53 and p63 was significantly increased in OL compared to normal mucosa (*p* < 0.01). | There is significantly higher expression of p53 and p63 proteins in OSCC and OL when compared to normal oral mucosa. Further mRNA studies by RT-qPCR will ascertain the exact role of p63 in oral carcinogenesis and also its relationship to the p53 protein. |
| Vigneswaran et al., 2007  USA | Cross-sectional | 42 OSCC (29/13)  9 Metastatic OSCC  25 OL | *TNFSF10*  *TNFRSF10A*  *TNFRSF10B*  *TNFRSF10C*  *TNFRSF10D* | TRAIL (Apo2L)  DR4, DR5, DcR1 and DcR2 | IHC | The expression of TRAIL and DcR2 were reduced in OL compared to normal mucosa. Mean expression of DR4, DR5 and DcR1 did not differ significantly between normal mucosa and OL. Expression levels of TRAIL-R in normal mucosa adjacent to OSCC demonstrated a strong correlation in the expression patterns (*p*<0.001). TRAIL was completely negative or weak stained in OSCC samples. | TRAIL is constitutively expressed in normal oral mucosa but its expression is gradually lost in OL and OSCC. Thus, loss of TRAIL expression is an early event during oral carcinogenesis. |
| Vijayakumar et al., 2020  India | Cross-sectional | 20 OSCC (18/2)  20 OL (20/0)  25 Normal (17/8) | *SOX2*  *POU5F1*  *WNT5A* | SOX2  OCT4  WNT5A | IHC | SOX2 expression percentage showed statistically significant difference among study groups (OSCC x OL: *p* = 0.024; OSCC x Normal: *p=* 0.028; OL x Normal: *p=* 0.000). Differentially expression of OCT4 between OSCC and OL was non-significant, while OSCC/Normal and OL/Normal were significant (*p=* 0.020 and 0.001 respectively). Expression of WNT5A among groups showed significant difference (*p=* 0.000). Normal tissue had significantly lower expression of WNT5A compared to OSCC and OL. | SOX2 is significantly associated with cancer stem cells in OSCC, increasing its expression from OL to OSCC, suggesting its use as a potential marker of proliferation. WNT5A positivity also significantly increased from OL to OSCC, with higher expression in peripheral tumor cells of tumor islands aiding in invasion, suggesting its use as a marker of tumor invasion and transformation from OL to OSCC. OCT4 expression was sparse and insignificant. |
| Von Zeidler et al., 2014  Brazil | Retrospective cohort | 12 OSCC (10/2)  31 OL (14/17)  9 Normal | *CDH1* | E-cadherin | IHC | In non-dysplastic OL, there was a decrease in the E-cadherin expression compared to normal mucosa (*p* = 0.006). A significant decrease in E-cadherin expression was observed in dysplastic OL compared to low risk OL (*p*=0.019). Further decreased E-cadherin expression was observed in OSCC compared to high risk OL (*p*=0.0001). | According to dysplastic changes, there was a reduction in E-cadherin expression. Thus, E-cadherin could be suggested as a novel biomarker to identify OL lesions with higher risk for malignant transformation. |
| Vora et al., 2006  India | Cross-sectional | 70 OSCC (65/5)  24 OL (24/0) | *TP53* | p53 | IHC | Accumulation of p53 was present in 88% of hyperplasia, 62% of dysplasia and 19% OSCC. P53 positivity was significantly higher in patients with OL than in OSCC patients (*p* = 0.0001). OL patients who show p53 expression have a higher risk of developing OSCC than those who do not show p53. | p53 accumulation is believed to be an early event in the neoplastic progression of disease in the tongue. P53 may thus play an important role in the initiation of OSCC. |
| Wagner et al., 2017  Brazil | Cross-sectional | 87 OSCC (78/9)  24 OL (16/8)  10 Normal | *TGFB1* | TGF-𝛽1 | IHC | Negative TGF-𝛽1 expression was detected in all normal mucosa samples. 69.6% of OL and 93.4% of OSCC samples exhibited high expression of TGF-𝛽1. Increasing levels of TGF-𝛽1 were observed from normal mucosa, to OL, to OSCC samples (*p*<0.00). | The results of the present study suggest that modifications in TGF-𝛽1expression may play a role in the acquisition of a malignant phenotype in the early stages of oral carcinogenesis. |
| Wang et al., 2009  China | Cross-sectional | 6 OSCC  6 OL |  | 85 differentially expressed proteins | 2-DE followed by ESI-Q-TOF-LCMS/  MS | 730 protein spots (85%) in OSCC tissues and 708 (84%) in OL tissues were reproducibly detected in all 12 runs. A total of 85 differentially expressed proteins (2-fold change and *p*<0.05) were identified, including 52 up-regulated and 33 down-regulated proteins. | Varying levels of differentially expressed proteins may play an important role in the malignant transformation of OL, especially the three homologs of PA28. These proteins may also be useful candidate markers of OSCC. |
| Wang et al., 2014  China | Cross-sectional | 60 OSCC (32//28)  30 OL (13/17)  15 Normal | *NOD1*  *RIPK2*  *CASP12*  *DEFB1*  *DEFB4A*  *DEFB103A* | NOD1  RIP2  Caspase 12  hBD1  hBD2  hBD3 | IHC | The expression of NOD1, RIP2, Caspase12 and hBD1, 2, 3 decreased gradually with the progression of OSCC. In the normal and OL tissues, moderate/strong staining intensity was observed, while no or weak staining was noted in OSCC. NOD1, RIP2, Caspase12 and hBD1, 2, 3 expression correlated significantly to diagnostic category. | hBDs may be useful markers of OSCC. NOD1, RIP2, and Caspase12 may serve as novel and potential biomarkers for development and progression of OSCC. |
| Wang et al., 2018  China | Cross-sectional | 17 OSCC  50 OL  (26/41) | *SLPI* | SLPI | IHC | It was observed a negative association between SLPI expression and the pathological differentiation of oral tissues from OL to OSCC (r=-0.4922, *p*<0.05). Also, the expression of SLPI between OL and OSCC was significantly different (*p*<0.05). | The overexpression of SLPI protein is negatively associated with the grade of OL  and it may have a potential role in prediction of malignant transformation of oral potentially malignant lesions. |
| Wang et al., 2019  China | Cross-sectional | 137 OSCC  101 OL | *PIWIL2* | PIWIL2 | IHC | Positive expression of PIWIL2 was detected in 75.2% of OSCC cases. High expression of PIWIL2 in OSCC was significantly associated with shorter cancer-free survival (*p* = 0.018). Expression of PIWIL2 in patients with OL was found to be strongly prognostic of malignant transformation (*p<* 0.001), suggesting that PIWIL2 might play a role in the early steps of oral cancer tumorigenesis. | PIWIL2, a novel cancer stem cell marker, is expressed predominantly in OSCC and OL and may be a predictive biomarker for OSCC free survival and risk of malignant transformation of OL. |
| Wilkman et al., 1998  Finland | Cross-sectional | 11 OSCC (5/6)  20 OL (11/9)  6 Normal (2/4) | *ERBB2* | c-erbB-2 | IHC | The expression of c-erbB-2 varied considerably between the three groups with an important correlation with the differentiation degree. The mean score was 1.0 in the normal group, 1.3 in the epithelial hyperplasias, 1.5 in the epithelial dysplasias, and 2.7 in the OSCC group. | The lower the degree of epithelial differentiation, the higher the expression of c-erbB-2 oncoprotein. c-erbB-2 might form a useful marker for such dynamic changes, with c-erbB-2 becoming positive upon malignant transformation. |
| Winter et al., 2011  Germany | Cross-sectional | 15 OSCC  15 OL  15 Normal | *CDK2AP1*  *S100A7* | DOC-1  S100-A7 | RT-qPCR  IHC | Compared to normal tissues, the expression of Doc-1 was decreased and of S100A7 was elevated in OL and OSCC (*p*<0.001) and correlated with the malignancy of these lesions. The relative gene expression of Doc-1 was 0.2 in OL, and not traceable in OSCCs. Whereas S100A7 gene expression was 24.4 in OL, and 356.0 in OSCC. | The changes in the expression of S100A7 and Doc-1 are traceable even in benign lesions, making them sensitive markers. Both genes seem to be correlated with the biological behavior of the investigated lesions, making them useful in the potential risk estimation of a suspicious oral lesion. |
| Xia et al., 2011  China | Cross-sectional | 35 OSCC  20 OL  13 Normal | *ACKR3* | CXCR7 | RT-qPCR  IHC | mRNA levels of chemokines and receptors in OL tissues were increased compared to normal tissues. CXCR7 was significantly upregulated in OL samples compared to normal epithelia. CXCR7 was expressed in 86% of OSCC, in 85% of OL and in 8% of normal tissues. The expression in OL (*p*=0.005) and OSCC (*p*=0.006) tissues respectively, were significantly higher than in normal epithelia. | These results support the hypothesis that chemokines and chemokine receptors are involved in oral carcinogenesis. CXCR7-CXCL12/CXCL11 axis might play an important role in oral carcinogenesis. |
| Xia et al., 2012  China | Cross-sectional | 40 OSCC  24 OL  13 Normal | *CXCL12*  *CXCR4* | CXCL12  CXCR4 | IHC | CXCR4 was expressed in 15.4% of normal tissues, in 37.5% of OL and in 60% of OSCC samples, with significant difference between normal tissue and OSCC (*p*=0.005). Expression of CXCL12 was observed in 50% of OL and in 62.5% OSCC cases, with significant difference from normal epithelia (7.7%) (*p=*0.01 and *p=*0.001, respectively). | The result support the hypothesis that CXCL12/CXCR4 may play an important role in oral premalignant stages, contributing to the oral carcinogenesis progression. |
| Xia et al., 2013  China/ USA | Retrospective Cohort | 22 OSCC (6/16)  66 OL (31/35) | *SMAD4* | SMAD4 | IHC | Expression of SMAD4 in OSCC was significantly lower than in the paired malignant transformed OL (*p*<0.001). 77.3% of malignant transformed OL and 39.4% of untransformed OL presented strong SMAD4 expression (*p*=0.002). Strong SMAD4 expression and high grade of dysplasia predicted the malignant transformation of OL better than either single factor (*p* = 0.007). | Patients whose OL lesions presented higher SMAD4 expression displayed a significantly higher rate of malignant transformation. The combination of SMAD4 expression and histological grade of dysplasia was a better predictor for the malignant transformation of OL. |
| Xu et al., 1995  USA | Cross-sectional | 29 HNSCC  31 OL  15 Normal | *KRT1*  *KRT8*  *KRT13*  *KRT19*  *IVL* | Keratin 1  Keratin 8  Keratin 13  Keratin 19  Involucrin | IHC | The expression of CK8 was significantly different between normal and dysplasia (*p*=0.024) and between normal and OSCC (*p=*0.0001). CK19 expression significantly differed between normal and hyperplasia, dysplasia and OSCC (*p=*0.001, *p*=0.002 and *p=*0.0001, respectively). | The present study demonstrated novel expression patterns of cytokeratins in HN tissues and suggests that CKI9 and CK8 may be useful markers of histopathological  progression of HNSCC carcinogenesis. |
| Yamada et al., 2015  Japan | Cross-sectional | 31 OSCC  22 OL  15 Normal | *SPARC* | Osteonectin | IHC | OL, carcinoma in situ, and OSCC, SPARC-positive cell ratio was much higher than in normal mucosa. However, there were no significant differences among OL, carcinoma in situ, and OSCC. There was a tendency that the stronger potential the lesion had, the higher ratio of SPARC they had. PL had the highest SPARC-positive cell ratio of leukoplakia. | SPARC seems to be associated with the early stages of the multi-step cancerization mechanism. Although SPARC may be useful to detect epithelial mutative changes, it is unsuitable as a prognosis predictive biomarker. |
| Ye et al., 2018  China | Cross-sectional | 10 OSCC  36 OL  10 Normal | *CD47* | CD47 | IHC | The mean expression score of CD47 in normal mucosa was 1.56±0.93, and its expression was significantly lower compared with dysplastic OL (5.67±2.50; *p*<0.0001) and OSCC (7.10±3.76; *p*=0.0057). | CD47 may be a potential biomarker for predicting the progression of OL to OSCC, and may serve as an important molecular target for novel therapies for oral cancer. |
| Yoshida et al., 2015  Japan | Cross-sectional | 12 OSCC  17 OL  15 Normal | *KRT14*  *KRT19* | Keratin 14  Keratin 19 | IHC  RT-qPCR | CK 14 expression in mild dysplasia, severe dysplasia and OSCC were 70.3±34.7%, 19.6±13.1% and 6.4±7.2%, respectively. CK 19 expression in mild dysplasia, severe dysplasia and OSCC were 0.8±1.3%, 27.6±34.4% and 48.0±34.62%, respectively. There was a significant difference between mild dysplasia and OSCC in both CK expressions (*p* < 0.01). | Decreased expression of CK 14 in normal tissue and elevated expression of CK 19 in association with increased dysplasia, may serve as an indicator of potential malignant transformation. |
| Zhang et al., 2017  South Korea/ China | Retrospective Cohort | 22 OSCC  160 OL (100/60)  18 Normal | *PTGS2*  *MET*  *CTNNB1*  *JUN IMP3*  *CA9*  *MKI67*  *CDKN2A*  *TP53*  *PDPN* | COX2  c-Met  β-catenin  c-Jun  IMP3  CA9  Ki-67  p16  p53  Podoplanin | IHC | Comparing the expression between normal mucosa and OL, all biomarkers except for c-Jun and β-integi showed a significant difference. Analyzing each biomarker in terms of malignant progression, all assessed markers showed statistical significance. The set of p53 and CA9 showed the highest accuracy (0.881) combined with age and dysplasia. | This study was the first prognostic nomogram developed to predict the malignant transformation in OL patients. The nomogram may be useful for an accurate prediction of the transformation and for planning intensive follow-up and treatment. |
| Zhu et al., 2018  China | Cross-sectional/ Retrospective cohort | 33 OSCC (11/12)  61 OL (34/27)  15 Normal (4/11) | *ATM*  *CHEK2*  *H2AX* | ATM  CHEK2  γH2AFX | IHC | ATM and γH2AFX expression gradually increased in normal tissue, OL and OSCC groups during carcinogenesis (*p*=0.005 and *p*=0.001, respectively). There was no difference in CHEK2 expression among the groups (*p*=0.074). Also, significant difference on high expression rate of ATM and γH2AFX in untransformed compared to transformed OL was observed (*p*=0.037 and *p*=0.01, respectively). | ATM and γH2AFX expression in OL tissue was associated with OSCC progression. Detection of ATM and γH2AFX may be a promising method for an early identification and risk evaluation of OSCC in patients with OL. |

Abbreviations: 2-DE - Two dimensional electrophoresis; CLIA - Chemiluminescent Enzyme Linked Immunoassay; ELISA - Enzyme-linked Immunosorbent Assay; HNSCC – Head and Neck Squamous Cell Carcinoma; IHC – Immunohistochemistry; LI- Labeling Index; OL – Oral Leukoplakia; OSCC – Oral Squamous Cells Carcinoma; SD – Standard Deviation; TMA – Tissue Microarray;

Appendix 6. Summary of descriptive characteristics of included studies assessing proteins in biofluids (n= 21)

| Author, year, country | Study design | Sample size (M/F) | Specimen | Gene | Protein | Method | Main results | Conclusion |
| --- | --- | --- | --- | --- | --- | --- | --- | --- |
| Ameena & Rathy, 2019  India | Cross-sectional | 30 OSCC (21/9)  30 OL (19/11)  30 Normal (22/8) | Saliva | *TNF* | TNF-α | ELISA | A significant difference was found in TNF-α level between OSCC and OL, OSCC and controls, and OL and controls (*p* = 0.000). Significant difference in TNF‑α level between well‑differentiated and moderate /poorly differentiated OSCC was observed (*p* = 0.000) | TNF-α may be a potential monitoring molecule for the transformation of premalignancy to malignancy. |
| Ankita et al., 2019  India | Cross-sectional | 15 OSCC (8/7)  15 OL (15/0)  15 Normal (7/8) | Saliva | *EDN1* | Endothelin-1 | ELISA | Salivary Endothelin-1 level in study groups was: 82.78 ± 5.9 pg/ml in OSCC, 57.76 ± 4.1 pg/ml in OL and 29.72 ± 14.1 pg/mL in healthy controls (*p* < 0.001). | This study demonstrates potential utility of  salivary analysis for ET-1 levels to monitor patients at risk for OSCC. |
| Babiuch et al., 2020  Poland | Cross-sectional | 9 OSCC  7 dysplastic OL  16 non-dysplastic OL | Saliva | *IL1A*  *IL6*  *CXCL8*  *TNF* | IL-1α  IL-6  IL-8  TNF-α | ELISA | Concentrations of IL-1α, IL-6, IL-8, and TNF-α were higher in saliva of OSCC patients than in controls (*p* = 0.017, 0.0012, 0.0001, and 0.0012, respectively). IL-8 levels were significantly higher in saliva of OED patients than in controls (*p* = 0.0492) and in OSCC patients compared to OED patients (*p* = 0.0345). Concentrations of IL-6, IL-8, and TNF-α were also higher in OSCC compared to non-dysplastic OL (*p* =0.0012, 0.0000, and 0.0492, respectively). | The increase in salivary levels of IL-6, IL-8, and TNF-α could be a useful indicator of malignant transformation. The higher levels of IL-8 and TNF-α in saliva of OSCC or OL patients may be caused by increased expression of these cytokines in pathological tissues. |
| Brailo et al., 2012  Croatia | Cross-sectional | 28 OSCC (22/6)  29 OL (13/16)  31 Normal (19/12) | Saliva  Serum | *IL1B*  *IL6*  *TNF* | IL-1β  IL-6  TNF-α | CLIA | Saliva: IL-1β and IL-6 were significantly higher in OSCC than in OL and control groups (*p*≤0.05), but there was not difference in TNF-α. Serum: IL-1β concentrations were not detected, there was not difference in IL-6 level, and TNF-α was significantly higher in control than in OL and OSCC (p≤0.05). | Increase in proinflammatory cytokines concentrations in saliva might reflect the development of oral cancer from oral leukoplakia. |
| Chang et al., 2013  Taiwan | Cross-sectional | 151 OSCC (142/9)  46 OL (44/2)  111 Normal (101/10) | Serum | *CRP*  *SELE*  *ICAM1*  *IL6*  *CSF1*  *MMP2*  *MMP9*  *TGFB1* | CRP  E-selectin  ICAM-1  IL-6  M-CSF  MMP-2  MMP-9  TGF-β1 | ELISA | TGF-β1, E-selectin, CRP, MMP-2, and MMP-9 were significantly elevated in OL, and all markers, except M-CSF, were further increased in OSCC compared to controls. MMP-9, CRP and TGF-β1 were highly correlated with disease progression (*p*<0.001). Logistic regression revealed MMP-9, MMP-2, TGF-β1, and CRP as best markers for distinguishing OL and OSCC patients from healthy individuals. | Inflammation plays a crucial role in the pathogenesis process of OSCC. By assessing the inflammation markers, physicians could potentially identify patients at risk of cancer transformation or relapse. |
| Deepthi et al., 2019  India | Cross-sectional | 30 OSCC (26/4)  30 OL (26/4)  30 Normal (20/10) | Saliva | *TNF* | TNF-α | ELISA | The mean and standard deviation of the salivary levels of TNF-α in OSCC, OL and control groups were 63.94 ± 56.05, 28.96 ± 20.94 and 5.75 ±3.98, respectively (*p*<0.01). Salivary TNF-α could serve as valuable biomarker for differentiating OL and OSCC from healthy controls with an AUC of 0.968 and 0.997, respectively (*p*<0.001). | The increasing levels of salivary TNF-α from control to OL to OSCC, supports the utility of saliva in biomarker estimation and in evaluating TNF-α as a prognostic marker, also as an indicator for the neoplastic transformation from OL to cancer. |
| Dikova et al., 2021  Spain | Cross-sectional | 66 OSCC (33/33)  33 OL (10/23)  25 Normal (9/16) | Saliva | *IL1A*  *IL6*  *CXCL8*  *CXCL10*  *CCL2*  *TNF*  *CCL14* | IL-1α  IL-6  IL-8  IP-10  MCP-1  TNF-α  HCC-1  PF-4 | Multiplex Immunoassay | Patients with OL have significantly higher salivary IL-6, IL-8, MCP-1, TNF-α, and HCC-1 than healthy controls (p<0.01). Notable elevations of IL-6, IL-8, TNF-α, HCC-1, and PF-4 levels in OSCC collated to OL sample cohorts (p<0.0001). The levels of six biomarkers including IL-6, IL-8, MCP-1, TNF-α, HCC-1, and PF-4 considerably increased in OSCC compared to control saliva (p<0.01). | Saliva-derived IL-6, IL-8, TNF-α, HCC-1, and PF-4 may discriminate between OSCC, OL patients, and healthy controls. These non-invasive biomarkers may serve a useful role in early disease detection, as well as for screening of patients at risk of developing oral cancer. Putative biomarkers used in combination may enhance their accuracy. |
| Ding et al., 2014  China | Cross-sectional | 98 OSCC (47/51)  14 OL  27 Normal | Serum | *CCL2*  *CCL3* | MCP-1  CCL3 | ELISA | CCL2 concentration was significantly lower in OSCC patients (67.81 pg/ml) than in OL (86.15 pg/ml, *p*<0.001) and healthy subjects (108.1 pg/ml *p*<0.0001). CCL3 level was significantly lower in healthy controls (118.3 pg/ml) than in the OL group (201.9 pg/ml, *p*<0.001) but no significantly different from OSCC group (153.9 pg/ml). | CCL2 and CCL3 are potential tumor biomarkers and participate in the progression of OSCC. The level of CCL2 and the ratio of CCL2/CCL3 seems to be useful prognostic parameters for OSCC. |
| Ding et al., 2015  China | Cross-sectional | 85 OSCC (41/44)  15 OL  28 Normal | Serum | *IL17F*  *VEGFA* | IL-17F  VEGF | ELISA  PCR | The mean±SEM level (pg/ml) of IL-17F was gradually downregulated from healthy (394.3 ±96.42), OL (169.6±50.58), to OSCC individuals (82.96±11.19) (*p*<0.05). Conversely, the mean±SEM level of VEGF was gradually upregulated from healthy (70.35±4.931), OL (101.4±10.82), to OSCC individuals (158.8±10.03) (*p*<0.05). | IL-17F seems to be a diagnostic biomarker that discriminate OSCC patients from healthy individuals. Also, the ratio of serum IL-17F/VEGF was a good diagnostic marker to discriminate OL and OSCC patients from healthy individuals. |
| Ding et al., 2020  China | Cross-sectional | 136 OSCC  68 OL  64 Normal | Serum  PBMC | *IL18*  *IL37* | IL-18  IL-37 | ELISA  RT-qPCR | IL-18 concentration (pg/mL) was significantly elevated in OSCC patients serum (179.7) than in OL (144.1, *p* = 0.0016) and controls (118.0, *p* < 0.0001). IL-37 concentration (pg/mL) was decreased in OSCC patients (75.02) than in OL (83.51, *p=*0.033) and controls (95.52, *p* < 0.0001). The ratio IL-18/IL-37 was higher in OSCC patients (2.643) than in OL (1.743, *p* < 0.0001) and controls (1.198, *p* < 0.0001). The relative expression of IL-18 was increased in OSCC compared with controls (*p =*0.006) and OL (*p=* 0.0176). IL-37 was much higher in controls than in OSCC (*p=* 0.0001) and OL individuals (*p=* 0.0069). | A differential expression pattern of IL-18 and IL-37 in serum and tumor tissues was observed. The ratio of IL-18/IL-37 may be used as a biomarker for OSCC and risk assessment of malignant transformation of OL. |
| Garg et al., 2017  India | Cross-sectional | 24 OSCC  24 OL  24 Normal | Saliva | *BIRC5* | Survivin | ELISA | The mean levels (pg/mL) of survivin were 0.199 in control group, 0.312 in OL group, and 0.430 in OSCC group. There was statistically significant difference between normal and OL groups, normal and OSCC, and OL and OSCC (*p*=0.00001). | Survivin may be considered as a marker for the increased risk of OL to undergo malignant transformation as well as demonstrate its role in the early process of carcinogenesis. |
| Gonçalves et al., 2017  Brazil | Cross-sectional | 20 OSCC (14/6)  80 OL (40/60)  20 Normal | Saliva | *HLA-G*  *IL10*  *TGFB1* | HLA-G  IL-10  TGF-β1 | ELISA | OL presented a significantly higher expression of HLA-G, -E, IL-10, TGF-β2 and -β3 than the control group (*p*<0.05). The expression of these proteins in OL was similar than the OSCC group (*p*>0.05). There was no significant difference between salivary sHLA-G, IL-10 and TGF-β levels in OL patients and those of the control group (*p*>0.05). | OL is characterized by high expression of HLA-G, -E, PD-L1, IL-10, TGF-β2 and -β3. Overexpression of these mediators in OL indicates the immune evasion potential of this lesion, which seems independent of cytological and proliferation/apoptosis status. |
| Guan et al., 2019  China | Cross-sectional | 45 OSCC (25/20)  40 OL  30 Normal | Serum | *POSTN* | Periostin | ELISA | Periostin expression in OSCC was significantly higher than in OL (*p*<0.01) and was correlated with tumor differentiation, TNM staging and lymph node metastasis (*p*< 0.05). Compared to normal mucosa, mRNA expression of periostin in OL and OSCC significantly increased (*p*<0.01). The serum periostin levels in OL and OSCC patients were significantly higher than in healthy subjects (*p* < 0.05). | Aberrant expression of periostin occurs in early stages of morphological changes of OL, suggesting that the increase in periostin expression may be an early event in the carcinogenesis of OL. |
| Hoffmann et al., 2011  Brazil | Cross-sectional | 20 OSCC (14/6)  14 OL (5/9)  20 Normal (14/6) | Saliva | *EDN1* | Endothelin-1 | ELISA | Salivary levels of ET-1 (range in fmol/ml): normal - from 0 to 9.629; OL - from 0 to 9.453; OSCC - from 0 to 7.554 (*p>*0.05). Also, there was no significant difference after excluding patients with hypertension history or patients with > 65 years, nor comparing males and females. | Although ET-1 might display an important role in OSCC, its salivary levels do not seem to be a good biomarker of OSCC grade or malignant transformation. |
| Nayyar & Khan, 2012  India | Cross-sectional | 30 OSCC  10 OL  25 Normal | Serum | *ALB* | Total protein  Albumin | Biuret test  Spectrophotometry | The level of serum total protein was 8.236 ± 1.5025 g/dL in controls, 9.85 ± 3.6788 g/dL in OL, 7.8 ±  3.1500 g/dL in OSCC (*p*=0.1305). The serum albumin level was 4.956 ± 1.0579 g/dL in controls, 3.79 ± 0.9410 g/dL in OL, 3.6933 ±1.2177 g/dL in OSCC (*p*<0.001). | There is still a need for further studies assessing sera levels of total protein and albumin to confirm their utility and to assess their role in the pathogenesis and their impact on the prognosis of OSCC. |
| Paneer Selvam & Sadaksharam, 2015  India | Cross-sectional | 25 OSCC (21/4)  25 OL (21/4)  25 Normal | Saliva | *IL6* | IL-6 | ELISA | The mean salivary IL-6 concentration among control, OL and OSCC groups were 9.68 ± 12.838, 43.00 ± 52.143, and 132.88 ± 59.098 pg/mL, respectively. There was statistically significant difference in IL-6 concentration among the groups (*p*<0.001). | The increase in salivary IL-6 in OL and OSCC might indicate the local production by tumor cells. The difference in its levels between the two lesions might also suggest the progression of precancer to cancer. |
| Pereira et al., 2015  India | Cross-sectional | 30 OSCC (19/11)  30 OL (25/5)  20 Normal (8/12) | Serum | *LDHD* | Lactate Dehydrogenase | Spectrophotometry | The mean serum LDH level was 339.90 IU/L in the control group while in OL group the mean serum LDH level was 488.67 IU/L (477.76 IU/L on mild dysplastic lesions and 502.92 IU/L on lesions with moderate dysplasia). The mean serum LDH level was 743.30 IU/L on well differentiated OSCC and 988.50 IU/L in moderately differentiated cases (*p*<0.001). | Serum LDH levels increase in oral premalignant lesions, such as OL, and OSCC. Serum LDH level assessment may be a valuable biochemical marker due to its simplicity and may be easily accepted by the patient. |
| Saddiwal et al., 2017  India | Cross-sectional | 15 OSCC (11/4)  15 OL (13/2)  15 Normal (10/5) | Serum | *B2M* | B2-microglobulin | CLIA | The b2-microglobulin levels were increased in moderately differentiated OSCC (2851.33 ± 514.00 ug/ml) compared to well differentiated (2709.78 ± 394.48 ug/ml) (*p*>0.05). The serum b2-microglobulin level progressively increased from mild dysplasia to moderate dysplasia to severe dysplasia (*p*<0.001). | This progressively increasing of b2-microglobulin level positively correlates with the degree of cellular atypia suggesting that this protein may serve as biochemical tool in assessing the malignant potential of premalignant lesions. |
| Shah et al., 2017  India | Cross-sectional | 50 OSCC (29/21)  50 OL (47/3)  50 Normal (22/28) | Serum | *CP* | Ceruloplasmin | Diagnostic kit – SensIT  ERBA CHEM. 5 PLUS | The mean ceruloplasmin serum level was 73.42± 24.37 for normal group, 80.05±27.52 for OL and 92.59 ± 26.51 mg/dl on OSCC group. It was observed significant difference comparing normal and OSCC groups (Mean difference=-19.17; *p=*0.004). | The serum levels of ceruloplasmin are associated with progression of the carcinogenesis process, thus, it could be a used as prediction biomarker of malignant transformation of oral precancer. |
| Shetty et al., 2012  India | Cross-sectional | 25 OSCC (20/5)  25 OL (20/5)  25 Normal (20/5) | Saliva | *LDHD* | Lactate Dehydrogenase | Spectrophotometry | Mean salivary LDH level (IU/L) in normal, OL and OSCC groups were 79.50± 4.67, 136.46 ± 3.36 and 148.77 ± 4.83, respectively. There was a statistically significant difference between normal and OL (*p*=0.001), OL and OSCC (*p*=0.001), and between normal and OSCC (*p*=0.0001). | Salivary LDH levels could be a reliable maker for oral cancer; thus, alterations in salivary LDH levels seem to be an important factor in pathogenesis of oral cancer. |
| Sivadasan et al., 2020  India | Cross-sectional | 30 OSCC (18/12)  15 OL (9/6)  15 Normal (11/4) | Saliva |  | 179 differentially abundant proteins | Mass spectrometry (LC-MS/MS)  ELISA | 179 proteins were found as differentially abundant from the patient groups compared to the healthy control. 73 proteins were identified as differentials in OL and 85 N0 and N+ OSCC cases. 8 proteins (CD44, COL5A1, S100A7, S100P, COL1A1, a1AT/SERPINA1, S100A11, and S100A15) were randomly selected for verification by ELISA and S100A7, S100P, CD44 and COL5A1 showed progressively increased expression in OL and OSCC (N0, N+) as compared to healthy controls (*p* < 0.05). | A set of 93 potential protein biomarkers to be used for early detection of OL and OSCC were identified with elevated levels in the saliva of OSCC patients. 30 candidates of this list were prioritized for clinical applications and 8 of them were further verified by ELISA. CD44, S100A7 and S100P showed significant potential for use as early detection markers in patients with dysplastic OL and OSCC. |

Abbreviations: Ck – Cytokeratin; CLIA - Chemiluminescent Enzyme Linked Immunoassay; ELISA - Enzyme-linked Immunosorbent Assay; HNSCC – Head and Neck Squamous Cell Carcinoma; IHC – Immunohistochemistry; LI- Labeling Index; OL – Oral Leukoplakia; OSCC – Oral Squamous Cells Carcinoma; PBMC - peripheral blood mononuclear cells; SD – Standard Deviation; TMA – Tissue Microarray;

Appendix 7. Risk of Bias assessed by the Joanna Briggs Institute Critical Appraisal Tools for Cohort (A) and Cross-sectional (B) studies. Risk of bias was categorized as High when the study reached up to 49% score “yes”, Moderate when the study reached between 50% and 69% score“yes”, and Low when the study reached more than 69% score“yes”.

**A.** Checklist for Cohort Studies

| **Authors** | **Q.1** | **Q.2** | **Q.3** | **Q.4** | **Q.5** | **Q.6** | **Q.7** | **Q.8** | **Q.9** | **Q.10** | **Q.11** | **% yes / risk** |
| --- | --- | --- | --- | --- | --- | --- | --- | --- | --- | --- | --- | --- |
| Ding et al., 2018 | N | Y | Y | Y | N | Y | U | Y | Y | NA | Y | 70%/ L |
| Fernández-Valle et al., 2016a | U | Y | Y | Y | N | Y | U | Y | Y | Y | Y | 81%/ L |
| Fernández-Valle et al., 2016b | U | Y | Y | Y | N | Y | U | Y | Y | Y | Y | 81%/ L |
| Hamidi et al., 2000 | N | Y | U | Y | N | Y | U | Y | Y | NA | N | 50%/ M |
| Kouketsu et al., 2019 | N | Y | U | N | N | Y | U | Y | Y | NA | Y | 50%/ M |
| Lin et al., 2010 | Y | Y | U | Y | Y | Y | U | Y | Y | NA | Y | 80%/ L |
| Nasser et al., 2011 | U | Y | Y | N | N | Y | U | Y | Y | NA | Y | 60%/ M |
| Nguyen et al., 2017 | N | Y | Y | N | N | Y | Y | Y | Y | N | Y | 63%/ M |
| Nogami et al., 2003 | Y | Y | U | N | N | Y | Y | Y | Y | Y | Y | 72%/ L |
| von Zeidler et al., 2014 | Y | Y | N | Y | N | Y | Y | N | Y | NA | Y | 70%/ L |
| Xia et al., 2013 | Y | Y | Y | Y | N | Y | U | Y | Y | NA | Y | 80%/ L |
| Zhang et al., 2017 | Y | Y | U | N | N | Y | Y | Y | Y | NA | Y | 70%/ L |
| Zhu et al., 2018 | Y | Y | Y | Y | Y | Y | U | Y | Y | NA | Y | 90%/ L |

Q1. Were the two groups similar and recruited from the same population? Q2. Were the exposures measured similarly to assign people to both exposed and unexposed groups? Q3. Was the exposure measured in a valid and reliable way? Q4. Were confounding factors identified? Q5. Were strategies to deal with confounding factors stated? Q6. Were the groups/participants free of the outcome at the start of the study (or at the moment of exposure)? Q7. Were the outcomes measured in a valid and reliable way? Q8. Was the follow up time reported and sufficient to be long enough for outcomes to occur? Q9. Was follow up complete, and if not, were the reasons to loss to follow up described and explored? Q10. Were strategies to address incomplete follow up utilized? Q11. Was appropriate statistical analysis used?

Y - Yes; N - No; U – Unclear; NA – Not applicable; H – High, M – Moderate; L – Low.

**B.** Checklist for Analytical Cross-Sectional Studies

| **Authors** | **Q.1** | **Q.2** | **Q.3** | **Q.4** | **Q.5** | **Q.6** | **Q.7** | **Q.8** | **% yes / risk** |
| --- | --- | --- | --- | --- | --- | --- | --- | --- | --- |
| Agarwal et al., 1998 | N | Y | Y | Y | N | N | Y | N | 50%/M |
| Agarwal et al., 1999 | N | Y | N | Y | N | N | Y | N | 37.5%/H |
| Aiswarya et al., 2019 | N | N | Y | Y | N | N | Y | Y | 50%/M |
| Ambatipudi et al., 2013 | N | Y | N | Y | Y | N | Y | Y | 62.5%/M |
| Ameena & Rathy, 2019 | Y | Y | N | N | Y | Y | N | Y | 62.5%/M |
| Angelin et al., 2020 | Y | N | Y | Y | Y | N | Y | N | 62.5%/M |
| Ankita et al., 2019 | Y | Y | U | Y | Y | Y | Y | Y | 87.5%/L |
| Aruldoss et al., 2016 | Y | Y | U | Y | Y | N | U | Y | 62.5%/ M |
| Babiuch et al., 2020 | Y | Y | Y | Y | Y | N | Y | Y | 87.5%/L |
| Bavle et al., 2020 | N | N | Y | N | N | N | N | N | 12.5%/H |
| Bernardes et al., 2014 | U | Y | Y | Y | N | N | U | N | 37.5%/ H |
| Brailo et al., 2012 | Y | Y | Y | Y | Y | N | U | Y | 75%/ L |
| Buajeeb et al., 2009 | N | Y | Y | Y | N | N | U | Y | 50%/ M |
| Chamorro-Petronacci et al., 2021 | Y | Y | Y | Y | Y | Y | Y | Y | 100% / L |
| Chang et al., 2013 | N | Y | Y | Y | Y | Y | U | Y | 75%/ L |
| Chaudhari et al., 2016 | Y | Y | U | Y | U | N | Y | Y | 62.5%/M |
| Chen et al., 2019 | N | Y | Y | Y | Y | N | Y | Y | 75%/L |
| De Freitas et al., 2014 | N | N | Y | Y | N | N | Y | Y | 50%/ M |
| De Vicente et al., 2019 | Y | Y | Y | Y | N | N | Y | Y | 75%/L |
| Deepthi et al., 2019 | Y | Y | Y | Y | U | N | Y | Y | 75%/L |
| Dikova et al., 2021 | U | Y | Y | Y | Y | Y | Y | Y | 87.5%/ L |
| Ding et al., 2012 | N | N | Y | Y | N | N | Y | Y | 50%/M |
| Ding et al., 2014 | Y | U | Y | U | N | N | U | Y | 37.5%/H |
| Ding et al., 2015 | Y | N | Y | Y | Y | N | Y | Y | 75%/ L |
| Ding et al., 2020 | Y | N | Y | Y | Y | N | Y | Y | 75%/L |
| Dong et al., 2014 | N | Y | Y | U | N | N | U | Y | 37.5%/ H |
| Duś-Ilnicka et al., 2020 | N | Y | Y | Y | Y | N | U | N | 50%/ M |
| Dwivedi et al., 2020 | N | N | Y | Y | N | N | Y | N | 37.5%/H |
| Eversole & Sapp, 1993 | N | N | U | Y | N | N | Y | Y | 37.5%/H |
| Fan et al., 2006 | N | N | Y | Y | N | N | U | Y | 37.5%/H |
| Feng et al., 2008 | N | Y | U | U | N | N | U | Y | 25%/ H |
| Fillies et al., 2007 | N | Y | Y | Y | N | N | U | N | 37.5%/H |
| Foki et al., 2020 | N | N | Y | U | N | N | Y | Y | 37.5%/H |
| Garg et al., 2017 | Y | N | U | Y | N | N | U | Y | 37.5%/H |
| Girod et al., 1998 | N | N | U | Y | N | N | U | Y | 25%/ H |
| Gonçalves et al., 2017 | Y | Y | Y | Y | U | N | Y | Y | 75%/ L |
| Guan et al., 2019 | Y | N | Y | Y | N | N | U | Y | 50%/ M |
| He et al., 2008 | Y | Y | Y | Y | N | N | Y | Y | 75%/ L |
| Herrera Costa et al., 2019 | N | Y | U | Y | Y | N | U | Y | 50%/ M |
| Hoffmann et al., 2011 | N | Y | Y | U | Y | Y | U | Y | 62.5%/ M |
| Hu et al., 2015 | N | N | Y | Y | N | N | Y | Y | 50%/ M |
| Humayun & Prasad, 2011 | Y | Y | U | Y | Y | N | N | Y | 62.5%/ M |
| Jing et al., 2019 | N | N | Y | U | Y | N | Y | Y | 50%/ M |
| Juneja et al., 2015 | N | Y | N | Y | Y | N | U | Y | 50%/ M |
| Kannan et al., 1994 | N | N | U | Y | N | N | Y | Y | 37.5%/ H |
| Khan et al., 2009 | N | Y | Y | Y | N | N | N | Y | 50%/ M |
| Kitamura et al., 2012 | N | Y | U | Y | Y | Y | Y | Y | 75%/ L |
| Klein et al., 2020 | N | Y | Y | Y | Y | N | U | Y | 62.5%/ M |
| Lameira et al., 2014 | N | Y | Y | Y | Y | U | Y | Y | 75%/ L |
| Lin et al., 2016 | Y | Y | Y | Y | Y | Y | Y | Y | 100%/ L |
| Liu et al., 2017 | N | Y | Y | N | Y | N | N | Y | 50%/ M |
| Logeswari et al., 2014 | N | N | Y | U | Y | U | U | Y | 37.5%/ H |
| Lopes et al., 2018 | N | Y | U | Y | Y | U | Y | Y | 62.5%/ M |
| Madan et al., 2015 | N | N | N | Y | N | N | U | Y | 25%/ H |
| Mao et al., 2020 | N | Y | Y | Y | U | U | Y | Y | 62.5%/ M |
| Markopoulos et al., 2009 | N | Y | Y | U | N | N | U | Y | 37.5%/ H |
| Matsubara et al., 2011 | N | Y | U | Y | N | N | Y | Y | 50%/M |
| Matta et al., 2007 | N | Y | Y | Y | Y | U | U | Y | 50%/M |
| Matta et al., 2009 | N | Y | Y | Y | Y | U | Y | Y | 75%/ L |
| Meng et al., 2011 | N | N | Y | Y | N | N | U | Y | 37.5%/ H |
| Moraes et al., 2019 | N | Y | Y | Y | Y | U | Y | Y | 75%/ L |
| Nakabayashi et al., 2014 | N | N | U | Y | N | N | U | Y | 25%/ H |
| Nanda et al., 2012 | N | N | Y | Y | N | N | U | Y | 37.5%/ H |
| Narashiman et al., 2014 | N | Y | Y | N | Y | N | U | Y | 50%/ M |
| Nayyar & Khan, 2012 | Y | N | U | Y | N | N | U | Y | 37.5%/ H |
| Negi et al., 2015 | N | N | U | Y | N | N | N | Y | 25%/ H |
| Ohkura et al., 2005 | N | Y | U | U | N | N | N | Y | 25%/ H |
| Palani et al., 2011 | N | Y | Y | Y | N | N | N | Y | 50%/ M |
| Pande et al., 1998 | N | Y | U | Y | Y | N | Y | Y | 62.5%/ M |
| Paneer Selvam & Sadaksharam, 2015 | Y | Y | Y | U | Y | Y | U | Y | 75%/ L |
| Patil et al., 2015 | N | N | Y | U | N | N | N | Y | 25%/ H |
| Pereira et al., 2015 | Y | N | U | Y | Y | Y | U | Y | 62.5%/ M |
| Pontes et al., 2009 | N | N | Y | Y | Y | U | Y | Y | 62.5%/ M |
| Pontes et al., 2013 | N | N | N | Y | N | N | Y | Y | 37.5%/ H |
| Poomsawat et al., 2010 | N | Y | Y | Y | N | N | U | Y | 50%/ M |
| Poomsawat et al., 2014 | N | Y | Y | Y | N | N | U | Y | 50%/ M |
| Priyanka et al., 2019 | N | N | Y | Y | N | N | U | U | 25%/ H |
| Ralhan et al., 1997 | Y | Y | Y | U | N | N | U | Y | 50%/ M |
| Rathee et al., 2021 | Y | Y | Y | U | Y | N | Y | Y | 75%/ L |
| Ravi et al., 1996 | N | N | U | Y | Y | Y | U | Y | 50%/ M |
| Renkonen et al., 2002 | N | N | Y | Y | N | N | Y | U | 37.5%/ H |
| Ries et al., 2021 | U | Y | Y | U | Y | U | Y | Y | 62.5%/ M |
| Routray et al., 2013 | N | N | Y | Y | N | N | Y | Y | 50%/ M |
| Sabitha et al., 2018 | N | N | U | Y | N | N | U | Y | 25%/ H |
| Saddiwal et al., 2017 | Y | Y | Y | Y | Y | U | U | Y | 75%/ L |
| Saileela et al., 2018 | N | N | Y | U | N | N | U | Y | 25%/ H |
| Sakthivel et al., 2020 | N | N | U | Y | N | N | U | Y | 25%/ H |
| Santhi et al., 2006 | Y | N | Y | U | N | N | U | Y | 37.5%/ H |
| Sawant et al., 2018 | N | N | Y | Y | N | N | Y | Y | 50%/ M |
| Servato et al., 2019 | N | Y | Y | Y | Y | Y | U | Y | 75%/ L |
| Shah et al., 2017 | Y | Y | U | Y | Y | Y | Y | Y | 87.5%/ L |
| Shetty et al., 2012 | Y | N | N | U | Y | Y | U | Y | 50%/ M |
| Shyam et al., 2014 | N | N | Y | Y | N | N | Y | Y | 50%/ M |
| Singh et al., 2019 | Y | Y | Y | Y | N | N | U | Y | 62.5%/ M |
| Singh et al., 2020 | Y | Y | Y | U | Y | Y | Y | Y | 87.5%/ L |
| Singla et al., 2018 | Y | Y | U | Y | N | N | U | Y | 50%/ M |
| Sivadasan et al., 2020 | N | Y | Y | U | Y | U | Y | Y | 62.5%/ M |
| Soares et al., 2006 | N | N | Y | Y | N | N | U | Y | 37.5%/ H |
| Soni et al., 2005 | N | Y | Y | Y | N | N | Y | Y | 62.5%/ M |
| Sravya et al., 2016 | N | N | Y | Y | N | N | U | Y | 37.5%/ H |
| Srinivasan & Jewell, 2001 | N | Y | Y | Y | Y | N | Y | Y | 75%/ L |
| Sudha & Hemavathy, 2011 | N | N | Y | U | N | N | U | Y | 25%/ H |
| Sun et al., 2016 | N | Y | Y | Y | N | N | N | Y | 50%/ M |
| Sundar et al., 2021 | N | N | N | U | N | N | Y | Y | 25%/ H |
| Sutariya & Manjunatha, 2016 | N | N | Y | Y | N | N | Y | U | 37.5%/ H |
| Tashiro et al., 2020 | N | Y | Y | Y | N | N | U | Y | 50%/ M |
| Thiem et al., 2017 | Y | N | Y | Y | N | N | Y | Y | 62.5%/ M |
| Thorup et al., 1998 | N | Y | U | Y | N | N | U | N | 25%/ H |
| Tripathi et al., 2010 | N | Y | Y | Y | Y | N | U | Y | 62.5%/ M |
| Turatti et al., 2005 | N | N | N | Y | N | N | U | Y | 25%/ H |
| Vadla et al., 2020 | N | N | Y | Y | N | N | N | N | 25%/ H |
| Varghese et al., 2011 | N | N | U | Y | N | N | U | Y | 37.5%/ H |
| Varun et al., 2014 | N | Y | N | Y | Y | N | U | Y | 50%/ M |
| Vigneswaran et al., 2007 | N | N | Y | Y | N | N | Y | Y | 50%/ M |
| Vijayakumar et al., 2020 | Y | Y | Y | U | N | N | U | Y | 50%/ M |
| Vora et al., 2006 | N | N | Y | Y | Y | N | U | Y | 50%/ M |
| Wagner et al., 2017 | Y | Y | Y | Y | Y | N | Y | Y | 87.5%/ L |
| Wang et al., 2009 | Y | N | Y | Y | N | N | Y | Y | 62.5%/ M |
| Wang et al., 2014 | N | Y | Y | U | Y | N | U | Y | 50%/ M |
| Wang et al., 2018 | N | Y | Y | Y | N | N | Y | Y | 62.5%/ M |
| Wang et al., 2019 | N | Y | Y | U | N | N | Y | Y | 50%/ M |
| Wilkman et al., 1998 | N | Y | Y | Y | N | N | U | N | 37.5%/ H |
| Winter et al., 2011 | Y | N | U | U | Y | Y | U | Y | 50%/ M |
| Xia et al., 2011 | N | N | Y | Y | N | N | U | Y | 37.5%/ H |
| Xia et al., 2012 | N | N | Y | Y | N | N | U | Y | 37.5%/ H |
| Xu et al., 1995 | N | N | Y | Y | N | N | U | Y | 37.5%/ H |
| Yamada et al., 2015 | N | N | Y | N | N | N | U | Y | 25%/ H |
| Ye et al., 2018 | N | N | Y | Y | Y | Y | U | Y | 62.5%/ M |
| Yoshida et al., 2015 | N | Y | U | Y | N | N | U | Y | 37.5%/ H |
| Zhu et al., 2018 | Y | Y | Y | Y | Y | Y | U | Y | 87.5%/ L |

Q1. Were the criteria for inclusion in the sample clearly defined? Q2. Were the study subjects and the setting described in detail? Q3. Was the exposure measured in a valid and reliable way? Q4. Were objective, standard criteria used for measurement of the condition? Q5. Were confounding factors identified? Q6. Were strategies to deal with confounding factors stated? Q7. Were the outcomes measured in a valid and reliable way? Q8. Was appropriate statistical analysis used?

Y - Yes; N- No; U – Unclear; H – High, M – Moderate; L – Low.

Appendix 8. Characteristics of assessed proteins among included studies (n=173).

| Gene | Protein | Uniprot ID | Biological function | Subcellular location | Number of studies |
| --- | --- | --- | --- | --- | --- |
| *ABCB1* | ATP-dependent translocase ABCB1 | P08183 | Translocates drugs and phospholipids across the membrane. Catalyzes the flop of phospholipids from the cytoplasmic to the exoplasmic leaflet of the apical membrane. | Plasma membrane | 1 |
| *ACKR3* | Atypical chemokine receptor 3 | P25106 | Controls chemokine levels and localization via high-affinity chemokine binding, resulting in chemokine sequestration, degradation, or transcytosis. Promotes cell growth and survival. | Plasma membrane Endosome | 1 |
| *AKT1* | RAC-alpha serine/threonine-protein kinase | P31749 | Regulate many processes including metabolism, proliferation, cell survival, growth and angiogenesis. | Nucleus Plasma Membrane | 3 |
| *ALB* | Albumin | P02768 | Binds water, Ca2+, Na+, K+, fatty acids, hormones, bilirubin and drugs. Its main function is the regulation of the colloidal osmotic pressure of blood. Major zinc transporter in plasma. | ERS | 1 |
| *ALDH1A1* | Retinal dehydrogenase 1 | P00352 | Can convert/oxidize retinaldehyde to retinoic acid. Binds free retinal and cellular retinol-binding protein-bound retinal | Cytosol | 1 |
| *ALDH2* | Aldehyde dehydrogenase, mitochondrial | P05091 | Involved in step 2 of the subpathway that synthesizes acetate from ethanol. | Mitochondrion | 1 |
| *ATM* | Serine-protein kinase ATM | Q13315 | Serine/threonine protein kinase which activates checkpoint signaling upon double strand breaks (DSBs), apoptosis and genotoxic stresses, thereby acting as a DNA damage sensor. | Nucleus | 2 |
| *AURKA* | Aurora kinase A | O14965 | Mitotic serine/threonine kinase that contributes to the regulation of cell cycle progression. Associates with the centrosome and the spindle microtubules during mitosis and plays a critical role in various mitotic events. | Cytoskeleton | 1 |
| *B2M** | Beta-2-microglobulin | P61769 | Component of the class I major histocompatibility complex (MHC). Involved in the presentation of peptide antigens to the immune system. | ERS | 1 |
| *BAX* | Apoptosis regulator BAX | Q07812 | Under stress conditions, undergoes a conformation change that causes translocation to the mitochondrion membrane, leading to the release of cytochrome c that then triggers apoptosis. Promotes activation of CASP3, and thereby apoptosis. | Mitochondrion outer membrane Cytoplasm | 2 |
| *BCL2* | Apoptosis regulator Bcl-2 | P10415 | Suppresses apoptosis in a variety of cell systems including factor-dependent lymphohematopoietic and neural cells. Regulates cell death by controlling the mitochondrial membrane permeability. | Nucleus membrane ERM Mitochondrion outer membrane | 6 |
| *BDNF* | Brain-derived neurotrophic factor | P23560 | Growth factor that participates in the differentiation, proliferation, and survival of neural cells and neurogenesis in the central and peripheral nervous systems. | ERS | 1 |
| *BHLHE40* | Class E basic helix-loop-helix protein 40 (DEC1) | O14503 | Member of the basic helix-loophelix (bHLH) transcription factor family, plays an important role in the development of chondrocytes and in the regulation of circadian rhythms. | Nucleus Cytoplasm | 1 |
| *BIRC5* | Baculoviral IAP repeat-containing protein 5 (Survivin) | O15392 | Dual roles in promoting cell proliferation and preventing apoptosis.  May play a role in neoplasia. | Nucleus Cytoskeleton Cytoplasm | 6 |
| *BMI1* | Polycomb complex protein BMI-1 | P35226 | Component of a Polycomb group multiprotein PRC1-like complex, which is required to maintain the transcriptionally repressive state of many genes throughout development. | Nucleus Cytoplasm | 2 |
| *BRAF* | Serine/threonine-protein kinase B-raf | P15056 | Protein kinase involved in the transduction of mitogenic signals from the cell membrane to the nucleus | Nucleus Plasma Membrane Cytoplasm | 1 |
| *BUB1B* | Mitotic checkpoint serine/threonine-protein kinase BUB1 beta | O60566 | Essential component of the mitotic checkpoint. Required for normal mitosis progression. | Cytoskeleton  Nucleus Cytoplasm | 1 |
| *CA9* | Carbonic anhydrase 9 | Q16790 | Participates in pH regulation. May be involved in the control of cell proliferation and transformation. Appears to be a novel specific biomarker for a cervical neoplasia. | Nucleus Plasma Membrane | 1 |
| *CASP12* | Inactive caspase-12 | Q6UXS9 | Has no protease activity. May reduce cytokine release in response to bacterial lipopolysaccharide during infections. Reduces activation of NF-kappa-B in response to TNF. | Cytosol Endoplasmic reticulum | 1 |
| *CASP3* | Caspase-3 | P42574 | Involved in the activation cascade of caspases responsible for apoptosis execution | Cytoplasm | 1 |
| *CCL14* | C-C motif chemokine 14 | Q16627 | Has weak activities on human monocytes and acts via receptors that also recognize MIP-1 alpha. It induces intracellular Ca2+ changes and enzyme release. Enhances the proliferation of CD34 myeloid progenitor cells. | ERS | 1 |
| *CCL2* | C-C motif chemokine 2 | P13500 | Signals through binding and activation of CCR2 and induces a strong chemotactic response and mobilization of intracellular calcium ions, especially in monocytes and basophils | ERS | 1 |
| *CCL3* | C-C motif chemokine 3 | P10147 | Monokine with inflammatory and chemokinetic properties. Binds to CCR1, CCR4 and CCR5. | ERS | 1 |
| *CCNDBP1* | Cyclin-D1-binding protein 1 | O95273 | May negatively regulate cell cycle progression. May act at least in part via inhibition of the cyclin-D1/CDK4 complex, thereby preventing phosphorylation of RB1 and blocking E2F-dependent transcription. | Nucleus Cytoplasm | 3 |
| *CD274* | Programmed cell death 1 ligand 1 (PD-L1) | Q9NZQ7 | The PDCD1-mediated inhibitory pathway is exploited by tumors to attenuate anti-tumor immunity and escape destruction by the immune system, thereby facilitating tumor survival. | Plasma membrane Endosome membrane | 3 |
| *CD4* | T-cell surface glycoprotein CD4 | P01730 | Integral membrane glycoprotein that plays an essential role in the immune response and serves multiple functions in responses against both external and internal offenses. | Plasma membrane | 1 |
| *CD47* | Leukocyte surface antigen CD47 | Q08722 | Has a role in both cell adhesion by acting as an adhesion receptor for THBS1 on platelets, and in the modulation of integrins. Receptor for SIRPA, binding to which prevents maturation of immature dendritic cells and inhibits cytokine production by mature dendritic cells. Interaction with SIRPG mediates cell-cell adhesion, enhances superantigen-dependent T-cell-mediated proliferation and costimulates T-cell activation. | Plasma membrane | 1 |
| *CD68* | Macrosialin | P34810 | Could play a role in phagocytic activities of tissue macrophages, both in intracellular lysosomal metabolism and extracellular cell-cell and cell-pathogen interactions. | Plasma membrane Lysosome membrane  Endosome membrane | 1 |
| *CDC6* | Cell division control protein 6 homolog | Q99741 | Involved in the initiation of DNA replication. Also participates in checkpoint controls that ensure DNA replication is completed before mitosis is initiated. | Nucleus Cytoplasm | 1 |
| *CDH1* | Cadherin-1 | P12830 | Involved in mechanisms regulating cell-cell adhesions, mobility and proliferation of epithelial cells. Has a potent invasive suppressor role. | Plasma membrane Endosome Golgi apparatus | 3 |
| *CDK2AP1* | Cyclin-dependent kinase 2-associated protein 1 | O14519 | Specific inhibitor of the cell-cycle kinase CDK2. | Cytosol Nucleus | 1 |
| *CDK4* | Cyclin-dependent kinase 4 | P11802 | Ser/Thr-kinase component of cyclin D-CDK4 (DC) complexes that phosphorylate and inhibit members of the retinoblastoma (RB) protein family including RB1 and regulate the cell-cycle during G1/S transition. | Nucleus  Nucleus membrane  Cytoplasm | 2 |
| *CDK6* | Cyclin-dependent kinase 6 | Q00534 | Serine/threonine-protein kinase involved in the control of the cell cycle and differentiation; promotes G1/S transition. Involved in initiation and maintenance of cell cycle exit during cell differentiation; prevents cell proliferation and regulates negatively cell differentiation, but is required for the proliferation of specific cell types. Delays senescence. | Nucleus Cytoskeleton Cytoplasm | 1 |
| *CDKN1A* | Cyclin-dependent kinase inhibitor 1 (p21) | P38936 | May be involved in p53/TP53 mediated inhibition of cellular proliferation in response to DNA damage. Inhibits cyclin-dependent kinase activity, preventing phosphorylation of critical cyclin-dependent kinase substrates and blocking cell cycle progression. Plays an important role in controlling cell cycle progression and DNA damage-induced G2 arrest | Nucleus Cytoplasm | 3 |
| *CDKN1B* | Cyclin-dependent kinase inhibitor 1B (p27) | P46527 | Important regulator of cell cycle progression. Inhibits the kinase activity of CDK2 bound to cyclin A. Involved in G1 arrest. | Nucleus Endosome Cytoplasm | 1 |
| *CDKN1C* | Cyclin-dependent kinase inhibitor 1C (p57kip2) | P49918 | Potent tight-binding inhibitor of several G1 cyclin/CDK complexes and, to lesser extent, of the mitotic cyclin B-CDC2. Negative regulator of cell proliferation. May play a role in maintenance of the non-proliferative state throughout life. | Nucleus | 1 |
| *CDKN2A** | Cyclin-dependent kinase inhibitor 2A (p16^INK4a^) | P42771 | Acts as a negative regulator of the proliferation of normal cells by interacting strongly with CDK4 and CDK6. This inhibits their ability to interact with cyclins D and to phosphorylate the retinoblastoma protein. | Nucleus Cytoplasm | 6 |
| *CHEK2* | Serine/threonine-protein kinase Chk2 | O96017 | Serine/threonine-protein kinase which is required for checkpoint-mediated cell cycle arrest, activation of DNA repair and apoptosis in response to the presence of DNA double-strand breaks. May also negatively regulate cell cycle progression during unperturbed cell cycles. | Nucleus | 1 |
| *CP* | Ceruloplasmin | P00450 | It is involved in iron transport across the cell membrane. | ERS | 1 |
| *CRP* | C-reactive protein | P02741 | Promotes agglutination, bacterial capsular swelling, phagocytosis and complement fixation through its calcium-dependent binding to phosphorylcholine. Can interact with DNA and histones and may scavenge nuclear material released from damaged circulating cells. | ERS | 1 |
| *CSF1* | Macrophage colony-stimulating factor 1 (M-CSF) | P09603 | Regulation of survival, proliferation and differentiation of hematopoietic precursor cells, especially mononuclear phagocytes, such as macrophages and monocytes. Promotes the release of proinflammatory chemokines, and thereby plays an important role in innate immunity and in inflammatory processes. Promotes reorganization of the actin cytoskeleton, regulates formation of membrane ruffles, cell adhesion and cell migration. | Plasma membrane ERS | 1 |
| *CTNNB1* | Catenin beta-1 | P35222 | Key downstream component of the canonical Wnt signaling pathway. Involved in the regulation of cell adhesion, as component of an E-cadherin:catenin adhesion complex. Acts as a negative regulator of centrosome cohesion. | Cytoskeleton Nucleus Plasma membrane Cytoplasm | 1 |
| *CXCL10* | C-X-C motif chemokine 10 | P02778 | Pro-inflammatory cytokine involved in a wide variety of processes such as chemotaxis, differentiation, and activation of peripheral immune cells, regulation of cell growth, apoptosis and modulation of angiostatic effects | ERS | 1 |
| *CXCL12* | Stromal cell-derived factor 1 | P48061 | Chemoattractant active on T-lymphocytes and monocytes. Activates the receptor CXCR4 to induce a rapid and transient rise in the level of intracellular calcium ions and chemotaxis. | ERS | 1 |
| *CXCL8* | Interleukin-8 | P10145 | Chemotactic factor that attracts neutrophils, basophils, and T-cells and activates neutrophils. It is released from several cell types in response to an inflammatory stimulus. | ERS | 1 |
| *CXCR4* | C-X-C chemokine receptor type 4 | P61073 | Receptor for CXCL12 that transduces a signal by increasing intracellular calcium ion levels and enhancing MAPK1/MAPK3 activation. Involved in the AKT signaling cascade. Plays a role in regulation of cell migration. Acts as a receptor for extracellular ubiquitin; leading to enhanced intracellular calcium ions and reduced cellular cAMP levels | Lysosome Endosome  Plasma membrane | 1 |
| *DEFB1* | Beta-defensin 1 (hBD-1) | P60022 | Has bactericidal activity. May act as a ligand for C-C chemokine receptor CCR6. | ERS | 1 |
| *DEFB103A* | Beta-defensin 103 (hBD-3) | P81534 | Exhibits antimicrobial activity against Gram-positive bacteria S.aureus and S.pyogenes, Gram-negative bacteria P.aeruginosa and E.coli and the yeast C.albicans. | ERS | 1 |
| *DEFB4A* | Beta-defensin 4A (hBD-2) | O15263 | Exhibits antimicrobial activity against Gram-negative bacteria and Gram-positive bacteria. May act as a ligand for C-C chemokine receptor CCR6. | ERS | 1 |
| *DSG2* | Desmoglein-2 | Q14126 | Component of intercellular desmosome junctions. Involved in the interaction of plaque proteins and intermediate filaments mediating cell-cell adhesion. | Plasma membrane | 1 |
| *DSP* | Desmoplakin | P15924 | Involved in the organization of the desmosomal cadherin-plakoglobin complexes into discrete plasma membrane domains and in the anchoring of intermediate filaments to the desmosomes. | Plasma membrane Cytoskeleton | 1 |
| *EDN1* | Endothelin-1 | P05305 | Probable ligand for G-protein coupled receptors EDNRA and EDNRB which activates PTK2B, BCAR1, BCAR3 and, GTPases RAP1 and RHOA cascade in glomerular mesangial cells | ERS | 2 |
| *EGFR* | Epidermal growth factor receptor | P00533 | Activates several signaling cascades (RAS-RAF-MEK-ERK, PI3 kinase-AKT, PLCgamma-PKC, STATs and NF-kappa-B) to convert extracellular cues into appropriate cellular responses. Positively regulates cell migration via interaction with CCDC88A/GIV. | Plasma, Nucleus and Golgi apparatus membrane ERM Endosome  ERS (isoform) | 3 |
| *ERBB2* | Receptor tyrosine-protein kinase erbB-2 | P04626 | Protein tyrosine kinase that is part of several cell surface receptor complexes, but that apparently needs a coreceptor for ligand binding. | Endosome Nucleus  Plasma membrane | 2 |
| *FOS* | Proto-oncogene c-Fos | P01100 | It is thought to have an important role in signal transduction, cell proliferation and differentiation | Nucleus Cytosol  Endoplasmic reticulum | 1 |
| *FOXP3* | Forkhead box protein P3 | Q9BZS1 | Transcriptional regulator crucial for the development and inhibitory function of regulatory T-cells. Plays an essential role in maintaining homeostasis of the immune system by allowing the acquisition of full suppressive function and stability of the Treg lineage, and by directly modulating the expansion and function of conventional T-cells. | Nucleus Cytoplasm | 1 |
| *GPRC5A* | Retinoic acid-induced protein 3 | Q8NFJ5 | Could be involved in modulating differentiation and maintaining homeostasis of epithelial cells. Functions as a negative modulator of EGFR signaling. May act as a lung tumor suppressor. | Plasma and Cytoplasmic vesicle membrane | 1 |
| *H2AX* | Histone H2AX | P16104 | Play a central role in transcription regulation, DNA repair, DNA replication and chromosomal stability. Required for checkpoint-mediated arrest of cell cycle progression in response to low doses of ionizing radiation and for efficient repair of DNA double strand breaks. | Nucleus Chromosome | 1 |
| *HLA-E* | HLA class I histocompatibility antigen, alpha chain E | P13747 | Non-classical major histocompatibility class Ib molecule involved in immune self-nonself discrimination. | Golgi apparatus membrane  Plasma membrane ERS | 1 |
| *HLA-G* | HLA class I histocompatibility antigen, alpha chain G | P17693 | Non-classical major histocompatibility class Ib molecule involved in immune regulatory processes at the maternal-fetal interface. Reprograms B cells toward an immune suppressive phenotype via LILRB1. May induce immune activation/suppression via intercellular membrane transfer. Through interaction with the inhibitory receptor CD160 on endothelial cells may control angiogenesis in immune privileged sites | Endosome membrane  ERM Plasma membrane ERS | 1 |
| *HNRNPK* | Heterogeneous nuclear ribonucleoprotein K | P61978 | Likely to play a role in the nuclear metabolism of hnRNAs. Plays an important role in p53/TP53 response to DNA damage, acting at the level of both transcription activation and repression. | Nucleus Cytoplasm | 1 |
| *HRAS** | GTPase Hras | P01112 | Involved in the activation of Ras protein signal transduction. Ras proteins bind GDP/GTP and possess intrinsic GTPase activity | Plasma membrane Golgi apparatus membrane | 1 |
| *HSPA5* | Endoplasmic reticulum chaperone BiP | P11021 | Endoplasmic reticulum chaperone involved in the correct folding of proteins and degradation of misfolded proteins. May also play a role in apoptosis and cell proliferation | Endoplasmic reticulum  Melanosome  Cytoplasm | 1 |
| *HSPB1* | Heat shock protein beta-1 | P04792 | Functions as a molecular chaperone probably maintaining denatured proteins in a folding-competent state. Plays a role in stress resistance and actin organization. May regulate phosphorylation and the axonal transport of neurofilament proteins | Cytoskeleton Nucleus Cytoplasm | 1 |
| *HSPBP1* | Hsp70-binding protein 1 | Q9NZL4 | Inhibits HSPA1A chaperone activity by changing the conformation of the ATP-binding domain of HSPA1A and interfering with ATP binding. Interferes with ubiquitination mediated by STUB1 and inhibits chaperone-assisted degradation of immature CFTR. | Endoplasmic reticulum Cytoplasm | 2 |
| *HSPD1* | 60 kDa heat shock protein, mitochondrial | P10809 | Facilitates the correct folding of imported proteins. May also prevent misfolding and promote the refolding and proper assembly of unfolded polypeptides generated under stress conditions in the mitochondrial matrix | Mitochondrion | 1 |
| *hTERT* | Telomerase reverse transcriptase | O94807 | Telomerase is a ribonucleoprotein enzyme essential for the replication of chromosome termini in most eukaryotes. Active in progenitor and cancer cells. | Nucleus | 2 |
| *ICAM1* | Intercellular adhesion molecule 1 | P05362 | Ligand for the leukocyte adhesion protein LFA-1 (integrin alpha-L/beta-2). During leukocyte trans-endothelial migration, promotes the assembly of endothelial apical cups | ERS Plasma Membrane | 1 |
| *IFNG* | Interferon gamma | P01579 | IFN-gamma, in addition to having antiviral activity, has important immunoregulatory functions. It is a potent activator of macrophages, it has antiproliferative effects on transformed cells and it can potentiate the antiviral and antitumor effects of the type I interferons. | ERS | 1 |
| *IL10* | Interleukin-10 | P22301 | Major immune regulatory cytokine that acts on many immune cells where it has profound anti-inflammatory functions, limiting excessive tissue disruption caused by inflammation. | ERS | 2 |
| *IL17F* | Interleukin-17F | Q96PD4 | Involved in stimulating the production of other cytokines and the proliferation of peripheral blood mononuclear cells and T-cells and in inhibition of angiogenesis | ERS | 1 |
| *IL18* | Interleukin-18 | Q14116 | A proinflammatory cytokine primarily involved in polarized T-helper 1 cell and natural killer cell immune responses. Activates NF-kappa-B, triggering synthesis of inflammatory mediators. | ERS Cytoplasm | 1 |
| *IL1A* | Interleukin-1 alpha | P01583 | Stimulates thymocyte proliferation by inducing IL-2 release, B-cell maturation and proliferation, and fibroblast growth factor activity. Involved in the inflammatory response, identified as endogenous pyrogens. | ERS Cytoplasm | 1 |
| *IL1B* | Interleukin-1 beta | P01584 | Potent proinflammatory cytokine. Induces prostaglandin synthesis, neutrophil influx and activation, T and B-cell activation and cytokine and antibody production, and fibroblast proliferation and collagen production. | ERS  Cytosol Lysosome | 1 |
| *IL37* | Interleukin-37 | Q9NZH6 | Suppressor of innate inflammatory and immune responses involved in curbing excessive inflammation. Suppresses proinflammatory cytokine production, but spares anti-inflammatory cytokines. Inhibits dendritic cell activation. | ERS  Cytosol Nucleus | 2 |
| *IL4* | Interleukin-4 | P05112 | Participates in at least several B-cell activation processes as well as of other cell types. It is a costimulator of DNA-synthesis. Stimulates autophagy in dendritic cells. | ERS | 1 |
| *IL6* | Interleukin-6 | P05231 | It is a potent inducer of the acute phase response. Plays an essential role in the final differentiation of B-cells. Acts on B-cells, T-cells, and hematopoietic progenitor cells. It induces myeloma and plasmacytoma growth and induces nerve cells differentiation. | ERS | 5 |
| *IMP3* | U3 small nucleolar ribonucleoprotein protein IMP3 | Q9NV31 | Required for the early cleavages during pre-18S ribosomal RNA processing. | Nucleus | 1 |
| *ITGA2* | Integrin alpha-2 (α2𝛽1 integrin) | P17301 | Receptor for laminin, collagen, collagen C-propeptides, fibronectin and E-cadherin. Responsible for adhesion of platelets and other cells to collagens, modulation of collagen and collagenase gene expression, and organization of newly synthesized extracellular matrix. | Nucleus Plasma Membrane | 1 |
| *ITGA3* | Integrin alpha-3 (α3𝛽l integrin) | P26006 | Receptor for fibronectin, laminin, collagen, epiligrin, thrombospondin and CSPG4. May participate in the adhesion, and matrix degradation processes, promoting cell invasion. | ERS Plasma Membrane | 1 |
| *ITGA6* | Integrin alpha-6 (α6𝛽4 integrin) | P23229 | Receptor for laminin in epithelial cells and it plays a critical structural role in the hemidesmosome | Plasma membrane | 1 |
| *ITGB6* | Integrin beta-6 (avb6 integrin) | P18564 | Receptor for fibronectin and cytotactin. Internalisation of integrin alpha-V/beta-6 via clathrin-mediated endocytosis promotes carcinoma cell invasion | Cytoskeleton Nucleus Plasma Membrane | 1 |
| *IVL* | Involucrin | P07476 | Part of the insoluble cornified cell envelope of stratified squamous epithelia. | Cytoskeleton Cytosol ERS Nucleus Plasma Membrane | 1 |
| *JUN* | Transcription factor AP-1 | P05412 | Involved in activated KRAS-mediated transcriptional activation of USP28 in colorectal cancer (CRC) cells. Binds to the USP28 promoter in colorectal cancer (CRC) cells. | Nucleus | 2 |
| *JUP* | Junction plakoglobin | P14923 | Common junctional plaque protein. The presence of plakoglobin in both the desmosomes and in the intermediate junctions suggests that it plays a central role in the structure and function of submembranous plaques. | Cytoskeleton adherens junction  desmosome  Membrane | 1 |
| *KCNC4* | Potassium voltage-gated channel subfamily C member 4 | Q03721 | This protein mediates the voltage-dependent potassium ion permeability of excitable membranes. | Plasma membrane | 1 |
| *KCNH2* | Potassium voltage-gated channel subfamily H member 2 | Q12809 | Pore-forming (alpha) subunit of voltage-gated inwardly rectifying potassium channel. Mediates the rapidly activating component of the delayed rectifying potassium current in heart | Plasma membrane | 1 |
| *KRAS* | GTPase Kras | P01116 | Plays an important role in the regulation of cell proliferation. Promotes oncogenic events by inducing transcriptional silencing of tumor suppressor genes in colorectal cancer cells. | Plasma membrane Cytosol | 1 |
| *KRT1* | Keratin, type II cytoskeletal 1 | P04264 | May regulate the activity of kinases such as PKC and SRC via binding to integrin beta-1 and the receptor of activated protein C kinase 1 | Plasma membrane | 2 |
| *KRT10* | Keratin, type I cytoskeletal 10 | P13645 | Plays a role in the establishment of the epidermal barrier on plantar skin. | ERS | 1 |
| *KRT13* | Keratin, type I cytoskeletal 13 | P13646 | There are two types of cytoskeletal and microfibrillar keratin: I (acidic; 40-55 kDa) and II (neutral to basic; 56-70 kDa). | Cytoskeleton Cytosol ERS Nucleus | 3 |
| *KRT14* | Keratin, type I cytoskeletal 14 | P02533 | The nonhelical tail domain is involved in promoting KRT5-KRT14 filaments to self-organize into large bundles and enhances the mechanical properties involved in resilience of keratin intermediate filaments | Nucleus Cytoplasm | 4 |
| *KRT17* | Keratin, type I cytoskeletal 17 | Q04695 | Regulates protein synthesis and epithelial cell growth by stimulating Akt/mTOR pathway. Involved in tissue repair. May be a marker of basal cell differentiation in complex epithelia. Acts as a promoter of epithelial proliferation by acting a regulator of immune response | Cytoplasm | 2 |
| *KRT18* | Keratin, type I cytoskeletal 18 | P05783 | When phosphorylated, plays a role in filament reorganization. Together with KRT8, is involved in interleukin-6 (IL-6)-mediated barrier protection | Nucleus | 2 |
| *KRT19* | Keratin, type I cytoskeletal 19 | P08727 | Involved in the organization of myofibers. Together with KRT8, helps to link the contractile apparatus to dystrophin at the costameres of striated muscle. | Cytoskeleton Cytosol ERS Plasma Membrane | 3 |
| *KRT4* | Keratin, type II cytoskeletal 4 | P19013 | There are two types of cytoskeletal and microfibrillar keratin: I (acidic; 40-55 kDa) and II (neutral to basic; 56-70 kDa). | Cytoskeleton Cytosol Nucleus | 1 |
| *KRT76* | Keratin, type II cytoskeletal 2 oral | Q01546 | Probably contributes to terminal cornification. | Cytoskeleton Cytosol ERS Nucleus | 1 |
| *KRT8* | Keratin, type II cytoskeletal 8 | P05787 | Together with KRT19, helps to link the contractile apparatus to dystrophin at the costameres of striated muscle. | Nucleus | 3 |
| *KRTAP5-6* | Keratin-associated protein 5-6 | Q6L8G9 | Keratinization | Cytoskeleton Cytosol | 1 |
| *LAMA3* | Laminin subunit alpha-3 (Laminin-5) | Q16787 | Is thought to mediate the attachment, migration and organization of cells into tissues during embryonic development. Laminin-5 is thought to be involved in cell adhesion, signal transduction and, differentiation of keratinocytes. | ERS | 1 |
| *LAMC2* | Laminin subunit gamma-2 | Q13753 | Binding to cells via a high affinity receptor, laminin is thought to mediate the attachment, migration and organization of cells into tissues during embryonic development by interacting with other extracellular matrix components. | ERS | 1 |
| *LDHD* | Probable D-lactate dehydrogenase, mitochondrial (LDH) | Q86WU2 | Catalytic activity | Mitochondrion | 3 |
| *MAP2K1* | Dual specificity mitogen-activated protein kinase kinase 1 | Q02750 | Depending on the cellular context, this pathway mediates diverse biological functions such as cell growth, adhesion, survival and differentiation, predominantly through the regulation of transcription, metabolism and cytoskeletal rearrangements. | Nucleus Cytoskeleton Cytoplasm | 1 |
| *MAP2K2* | Dual specificity mitogen-activated protein kinase kinase 2 | P36507 | Catalyzes the concomitant phosphorylation of a threonine and a tyrosine residue in a Thr-Glu-Tyr sequence located in MAP kinases. Activates the ERK1 and ERK2 MAP kinases | Cytoplasm  Membrane | 1 |
| *MAPK1** | Mitogen-activated protein kinase 1 | P28482 | Serine/threonine kinase which acts as an essential component of the MAP kinase signal transduction pathway. The MAPK/ERK cascade mediates cell growth, adhesion, survival and differentiation through the regulation of transcription, translation, cytoskeletal rearrangements. | Cytoskeleton  Nucleus Cytoplasm | 2 |
| *MAPK3* | Mitogen-activated protein kinase 3 | P27361 | Serine/threonine kinase which acts as an essential component of the MAP kinase signal transduction pathway. The MAPK/ERK cascade mediates cell growth, adhesion, survival and differentiation through the regulation of transcription, translation, cytoskeletal rearrangements. | Nucleus Cytoplasm | 2 |
| *MCM3* | DNA replication licensing factor MCM3 | P25205 | Acts as component of the MCM2-7 complex which is the putative replicative helicase essential for 'once per cell cycle' DNA replication initiation and elongation in eukaryotic cells. Required for DNA replication and cell proliferation. | Nucleus | 1 |
| *MCM7* | DNA replication licensing factor MCM7 | P33993 | Acts as component of the MCM2-7 complex which is the putative replicative helicase essential for 'once per cell cycle' DNA replication initiation and elongation in eukaryotic cells. Required for S-phase checkpoint activation upon UV-induced damage. | Nucleus | 1 |
| *MDM2* | E3 ubiquitin-protein ligase Mdm2 | Q00987 | Mediates ubiquitination of p53/TP53, leading to its degradation by the proteasome. Inhibits p53/TP53- and p73/TP73-mediated cell cycle arrest and apoptosis | Nucleus Cytoplasm | 2 |
| *MET* | Hepatocyte growth factor receptor | P08581 | Regulates many physiological processes including proliferation, scattering, morphogenesis and survival. | ERS | 1 |
| *MKI67* | Proliferation marker protein Ki-67 | P46013 | To maintain individual mitotic chromosomes dispersed in the cytoplasm following nuclear envelope disassembly; play a key role in cell proliferation. | Nucleus | 10 |
| *MLH1* | DNA mismatch repair protein Mlh1 | P40692 | Heterodimerizes with PMS2 to form MutL alpha, a component of the post-replicative DNA mismatch repair system (MMR). | Nucleus Chromosome | 1 |
| *MMP1* | Interstitial collagenase | P03956 | Cleaves collagens of types I, II, and III at one site in the helical domain | ERS | 1 |
| *MMP2* | 72 kDa type IV collagenase | P08253 | Ubiquitinous metalloproteinase that is involved in diverse functions such as remodeling of the vasculature, angiogenesis, tissue repair, tumor invasion, inflammation, and atherosclerotic plaque rupture. | ERS Nucleus Mitochondrion Cytoplasm | 1 |
| *MMP9* | Matrix metalloproteinase-9 | P14780 | May play an essential role in local proteolysis of the extracellular matrix and in leukocyte migration. | ERS | 1 |
| *MT2A* | Metallothionein-2 | P02795 | Metallothioneins have a high content of cysteine residues that bind various heavy metals; these proteins are transcriptionally regulated by both heavy metals and glucocorticoids. | Cytosol Nucleus Cytoplasm | 1 |
| *MTOR* | Serine/threonine-protein kinase mTOR | P42345 | Serine/threonine protein kinase which is a central regulator of cellular metabolism, growth and survival in response to hormones, growth factors, nutrients, energy and stress signals | Lysosome Nucleus ERM  Golgi apparatus and Mitochondrion outer membrane Cytoplasm | 1 |
| *MTUS1* | Microtubule-associated tumor suppressor 1 | Q9ULD2 | Cooperates with AGTR2 to inhibit ERK2 activation and cell proliferation. Isoform 1 inhibits breast cancer cell proliferation, delays the progression of mitosis by prolonging metaphase and reduces tumor growth. | Nucleus Plasma membrane Golgi apparatus Mitochondrion Cytoskeleton | 1 |
| *MUC1* | Mucin-1 | P15941 | Can act both as an adhesion and an anti-adhesion protein. May provide a protective layer on epithelial cells against bacterial and enzyme attack. | Plasma membrane  ERS | 1 |
| *MUC4* | Mucin-4 | Q99102 | May play a role in tumor progression. Ability to promote tumor growth may be mainly due to repression of apoptosis as opposed to proliferation. Has anti-adhesive properties. Plays an important role in cell proliferation and differentiation of epithelial cells. | ERS Plasma Membrane | 1 |
| *MYC* | Myc proto-oncogene protein | P01106 | Activates the transcription of growth-related genes. Binds to the VEGFA promoter, promoting VEGFA production and subsequent sprouting angiogenesis. Regulator of somatic reprogramming, controls self-renewal of embryonic stem cells. | Nucleus | 1 |
| *NES* | Nestin | P48681 | Promotes the disassembly of phosphorylated vimentin intermediate filaments (IF) during mitosis and may play a role in the trafficking and distribution of IF proteins and other cellular factors to daughter cells during progenitor cell division. Required for survival, renewal and mitogen-stimulated proliferation of neural progenitor cells. | Cytoskeleton Cytoplasm | 1 |
| *NFKB1* | Nuclear factor NF-kappa-B p105 subunit (NF-kB) | P19838 | Pleiotropic transcription factor present in almost all cell types and is the endpoint of a series of signal transduction events that are initiated by stimuli related to many biological processes such as inflammation, immunity, differentiation, cell growth, tumorigenesis and apoptosis. | Nucleus Cytoplasm | 2 |
| *NOD1* | Nucleotide-binding oligomerization domain-containing protein 1 | Q9Y239 | Enhances caspase-9-mediated apoptosis. Induces NF-kappa-B activity via RIPK2 and IKK-gamma. | Plasma membrane Cytoplasm | 1 |
| *NOS2* | Nitric oxide synthase, inducible (iNOS) | P35228 | Produces nitric oxide which is a messenger molecule with diverse functions throughout the body. Involved in inflammation, enhances the synthesis of proinflammatory mediators such as IL6 and IL8 | Cytosol | 2 |
| *NOTCH1** | Neurogenic locus notch homolog protein 1 | P46531 | Affects the implementation of differentiation, proliferation and apoptotic programs. Involved in angiogenesis; negatively regulates endothelial cell proliferation and migration and angiogenic sprouting. Involved in the maturation of both CD4+ and CD8+ cells in the thymus. | Plasma membrane Nucleus | 1 |
| *NRAS* | GTPase Nras | P01111 | Ras proteins bind GDP/GTP and possess intrinsic GTPase activity. | Plasma membrane Golgi apparatus | 1 |
| *NTRK2* | BDNF/NT-3 growth factors receptor | Q16620 | Involved in the development and maturation of the central and peripheral nervous systems through regulation of neuron survival, proliferation, migration, differentiation, and synapse formation and plasticity. | Endosome Plasma membrane | 1 |
| *PCNA* | Proliferating cell nuclear antigen | P12004 | Auxiliary protein of DNA polymerase delta and is involved in the control of eukaryotic DNA replication by increasing the polymerase's processibility during elongation of the leading strand | Nucleus | 3 |
| *PDCD1* | Programmed cell death protein 1 (PD-1) | Q15116 | Inhibitory receptor on antigen activated T-cells that plays a critical role in induction and maintenance of immune tolerance to self | Plasma membrane | 1 |
| *PDPN* | Podoplanin | Q86YL7 | Mediates effects on cell migration and adhesion through its different partners | Plasma membrane Cytosol | 4 |
| *PEBP1* | Phosphatidylethanolamine-binding protein 1 (RKIP) | P30086 | Binds ATP, opioids and phosphatidylethanolamine. Serine protease inhibitor which inhibits thrombin, neuropsin and chymotrypsin but not trypsin | Cytosol ERS Nucleus | 1 |
| *PITX1* | Pituitary homeobox 1 | P78337 | Sequence-specific transcription factor that binds gene promoters and activates their transcription. May play a role in the development of anterior structures, and in particular, the brain and facies and in specifying the identity or structure of hindlimb | Nucleus | 1 |
| *PIWIL2* | Piwi-like protein 2 | Q8TC59 | Plays a key role in germ cell maintenance in the testis and is widely expressed in colon, breast, prostate, gastrointestinal, ovarian, soft tissue, and endometrial cancers, but not in normal somatic cells and stem cells. | Nucleus Cytoplasm | 1 |
| *POSTN* | Periostin | Q15063 | Induces cell attachment and spreading and plays a role in cell adhesion. | Golgi apparatus ERS | 1 |
| *POU5F1* | POU domain, class 5, transcription factor 1 | Q01860 | Forms a trimeric complex with SOX2 or SOX15 on DNA and controls the expression of a number of genes involved in embryonic development. Critical for early embryogenesis and for embryonic stem cell pluripotency. | Nucleus Cytoplasm | 1 |
| *PTGS2* | Prostaglandin G/H synthase 2 (COX-2) | P35354 | Dual cyclooxygenase and peroxidase in the biosynthesis pathway of prostanoids with a particular role in the inflammatory response. Plays a role in the generation of resolution phase interaction products (resolvins) during both sterile and infectious inflammation. | Nucleus inner and outer membrane  Endoplasmic reticulum | 5 |
| *RAF1* | RAF proto-oncogene serine/threonine-protein kinase (Raf-1) | P04049 | Acts as a regulatory link between the membrane-associated Ras GTPases and the MAPK/ERK cascade, and this critical regulatory link functions as a switch determining cell fate decisions including proliferation, differentiation, apoptosis, survival and oncogenic transformation. | Mitochondrion Nucleus Plasma membrane | 1 |
| *RB1** | Retinoblastoma-associated protein (pRb) | P06400 | Key regulator of entry into cell division that acts as a tumor suppressor. | Nucleus | 4 |
| *RIPK2* | Receptor-interacting serine/threonine-protein kinase 2 | O43353 | Serine/threonine/tyrosine kinase that plays an essential role in modulation of innate and adaptive immune responses | Cytoskeleton Cytosol | 1 |
| *RPS6* | 40S ribosomal protein S6 | P62753 | May play an important role in controlling cell growth and proliferation through the selective translation of particular classes of mRNA | Cytosol Endoplasmic reticulum Nucleus | 1 |
| *S100A7* | Protein S100-A7 | P31151 | Plays a role in the pathogenesis of inflammatory skin disease, as a chemotactic factor for hematopoietic cells, and a role in early stages of breast tumor progression in association with the development of the invasive phenotype. | ERS Cytoplasm | 2 |
| *SELE* | E-selectin | P16581 | Cell-surface glycoprotein with a role in immunoadhesion. Mediates the adhesion of blood neutrophils in cytokine-activated endothelium through interaction with SELPLG/PSGL1. May have a role in capillary morphogenesis. | Plasma membrane | 1 |
| *SLPI* | Antileukoproteinase | P03973 | Plays a role in regulating the activation of NF-kappa-B and inflammatory responses. Required for normal wound healing, probably by preventing tissue damage by limiting protease activity. Required for normal differentiation and proliferation of bone marrow myeloid cells. | ERS | 1 |
| *SMAD4** | Mothers against decapentaplegic homolog 4 | Q13485 | Component of the multimeric SMAD3/SMAD4/JUN/FOS complex which forms at the AP1 promoter site; required for synergistic transcriptional activity in response to TGF-beta. May act as a tumor suppressor. | Nucleus Cytoplasm | 1 |
| *SNAI1* | Zinc finger protein SNAI1 (SNAIL) | O95863 | Involved in induction of the epithelial to mesenchymal transition (EMT), formation and maintenance of embryonic mesoderm, growth arrest, survival and cell migration. | Nucleus Cytoplasm | 1 |
| *SOX2* | Transcription factor SOX-2 | P48431 | Transcription factor that forms a trimeric complex with OCT4 on DNA and controls the expression of a number of genes involved in embryonic development | Nucleus | 2 |
| *SPARC* | SPARC (Osteonectin) | P09486 | Appears to regulate cell growth through interactions with the extracellular matrix and cytokines. | ERS | 2 |
| *SPP1* | Osteopontin | P10451 | Seems to form an integral part of the mineralized matrix. Probably important to cell-matrix interaction. Involved in enhancing production of interferon-gamma and IL-12 and reducing production of IL-10 and is essential in the pathway that leads to type I immunity. | ERS | 1 |
| *STAG2* | Cohesin subunit SA-2 | Q8N3U4 | Component of cohesin complex, a complex required for the cohesion of sister chromatids after DNA replication. | Nucleus Chromosome | 1 |
| *STAT3* | Signal transducer and activator of transcription 3 | P40763 | Signal transducer and transcription activator that mediates cellular responses. Involved in cell cycle regulation by inducing the expression of key genes for the progression from G1 to S phase. May play an apoptotic role by transctivating BIRC5 expression | Nucleus Cytoplasm | 1 |
| *STMN1* | Stathmin | P16949 | Involved in the regulation of the microtubule (MT) filament system by destabilizing microtubules. Prevents assembly and promotes disassembly of microtubules | Cytoskeleton | 1 |
| *TEK* | Angiopoietin-1 receptor | Q02763 | Tyrosine-protein kinase that acts as cell-surface receptor and regulates angiogenesis, endothelial cell survival, proliferation, migration, adhesion and cell spreading, reorganization of the actin cytoskeleton, and maintenance of vascular quiescence | ERS Cytoskeleton Plasma membrane | 1 |
| *TGFA* | Protransforming growth factor alpha | P01135 | TGF alpha is a mitogenic polypeptide that is able to bind to the EGF receptor/EGFR and to act synergistically with TGF beta to promote anchorage-independent cell proliferation | ERS Plasma membrane | 1 |
| *TGFB1* | Transforming growth factor beta-1 proprotein | P01137 | Regulates the growth and differentiation of various cells. Involved in various processes, such as normal development, immune function, microglia function and responses to neurodegeneration | ERS | 4 |
| *TGFB2* | Transforming growth factor beta-2 proprotein | P61812 | Multifunctional protein that regulates various processes such as angiogenesis and heart development | ERS | 1 |
| *TGFB3* | Transforming growth factor beta-3 proprotein | P10600 | Multifunctional protein that regulates embryogenesis and cell differentiation and is required in various processes such as secondary palate development | ERS | 1 |
| *TGFBR1* | TGF-beta receptor type-1 | P36897 | Transduces the TGFB1, TGFB2 and TGFB3 signal from the cell surface to the cytoplasm and regulates a plethora of physiological and pathological processes including cell cycle arrest in epithelial and hematopoietic cells, control of mesenchymal cell proliferation and differentiation, wound healing, extracellular matrix production, immunosuppression and carcinogenesis. | Plasma membrane | 1 |
| *TGFBR2** | TGF-beta receptor type-2 | P37173 | Transduces the TGFB1, TGFB2 and TGFB3 signal from the cell surface to the cytoplasm and regulates a plethora of physiological and pathological processes including cell cycle arrest in epithelial and hematopoietic cells, control of mesenchymal cell proliferation and differentiation, wound healing, extracellular matrix production, immunosuppression and carcinogenesis. | Plasma membrane | 1 |
| *TGFBR3* | TGF-beta receptor type-3 | Q03167 | Binds to TGF-beta. Could be involved in capturing and retaining TGF-beta for presentation to the signaling receptors. | Plasma membrane ERS | 1 |
| *TGM3* | Protein-glutamine gamma-glutamyltransferase E | Q08188 | Catalyzes the calcium-dependent formation of isopeptide cross-links between glutamine and lysine residues in various proteins, as well as the conjugation of polyamines to proteins. | ERS Plasma Membrane Cytoplasm | 1 |
| *TNF* | Tumor necrosis factor | P01375 | Cytokine that binds to TNFRSF1A/TNFR1 and TNFRSF1B/TNFBR. It is mainly secreted by macrophages and can induce cell death of certain tumor cell lines. | Plasma membrane ERS | 4 |
| *TNFRSF10A* | Tumor necrosis factor receptor superfamily member 10A | O00220 | Receptor for the cytotoxic ligand TNFSF10/TRAIL. The resulting death-inducing signaling complex performs caspase-8 proteolytic activation which initiates the subsequent cascade of caspases mediating apoptosis | Cytosol Plasma membrane | 1 |
| *TNFRSF10B* | Tumor necrosis factor receptor superfamily member 10B | O14763 | Receptor for the cytotoxic ligand TNFSF10/TRAIL. The resulting death-inducing signaling complex performs caspase-8 proteolytic activation which initiates the subsequent cascade of caspases mediating apoptosis | Plasma membrane | 1 |
| *TNFRSF10C* | Tumor necrosis factor receptor superfamily member 10C | O14798 | Receptor for the cytotoxic ligand TNFSF10/TRAIL. May protect cells against TRAIL mediated apoptosis by competing with TRAIL-R1 and R2 for binding to the ligand. | Plasma membrane | 1 |
| *TNFRSF10D* | Tumor necrosis factor receptor superfamily member 10D | Q9UBN6 | Receptor for the cytotoxic ligand TRAIL. Contains a truncated death domain and hence is not capable of inducing apoptosis but protects against TRAIL-mediated apoptosis. | Plasma membrane | 1 |
| *TNFSF10* | Tumor necrosis factor ligand superfamily member 10 | P50591 | Cytokine that binds to TNFRSF10A/TRAILR1, TNFRSF10B/TRAILR2, TNFRSF10C/TRAILR3, TNFRSF10D/TRAILR4 and possibly also to TNFRSF11B/OPG. Induces apoptosis. | Plasma membrane ERS | 1 |
| *TP53** | Cellular tumor antigen p53 | P04637 | Tumor suppressor in many tumor types; induces growth arrest or apoptosis depending on the physiological circumstances and cell type. Involved in cell cycle regulation as a trans-activator that acts to negatively regulate cell division by controlling a set of genes required for this process. | Nucleus, Mitochondrion matrix, Cytoskeleton Endoplasmic reticulum Cytoplasm | 15 |
| *TP63** | Tumor protein 63 (p63) | Q9H3D4 | Acts as a sequence specific DNA binding transcriptional activator or repressor. Plays a role in the regulation of epithelial morphogenesis. | Nucleus | 2 |
| *TWIST1* | Twist-related protein 1 | Q15672 | Acts as a transcriptional regulator. Also represses expression of proinflammatory cytokines such as TNFA and IL1B. | Nucleus | 1 |
| *VEGFA* | Vascular endothelial growth factor A | P15692 | Growth factor active in angiogenesis, vasculogenesis and endothelial cell growth. Induces endothelial cell proliferation, promotes cell migration, inhibits apoptosis and induces permeabilization of blood vessels. | ERS | 2 |
| *WNT5A* | Protein Wnt-5a | P41221 | Stimulates cell migration. Decreases proliferation, migration, invasiveness and clonogenicity of carcinoma cells and may act as a tumor suppressor | ERS | 1 |
| *YWHAZ* | 14-3-3 potein zeta/delta | P63104 | Adapter protein implicated in the regulation of a large spectrum of both general and specialized signaling pathways. | Cytoplasm,  Cytosol, ERS Mitochondrion Nucleus | 1 |

*HNSCC Driver Genes

Abbreviation: ERM = Endoplasmatic reticulum membrane; ERS = Extracellular region or secreted; NS = Not significant

# Appendix 9. Genes enriched in the Top-4 (FDR q-value) biological processes, molecular functions, and cellular components.

**a. Biological Processes**

| **Regulation of  programmed cell death** | | **Regulation of  cell death** | | **Regulation of cell  population proliferation** | | **Regulation of apoptotic  process** | |
| --- | --- | --- | --- | --- | --- | --- | --- |
| *ACKR3* | *KRT18* | *ACKR3* | *KRAS* | *ACKR3* | *ITGA2* | *ACKR3* | *KRT18* |
| *AKT1* | *MDM2* | *AKT1* | *KRT18* | *AKT1* | *JUN* | *AKT1* | *MDM2* |
| *ALB* | *MET* | *ALB* | *MDM2* | *ATM* | *JUP* | *ALB* | *MMP2* |
| *ATM* | *MMP2* | *ATM* | *MET* | *B2M* | *KRAS* | *ATM* | *MMP9* |
| *AURKA* | *MMP9* | *AURKA* | *MMP2* | *BAX* | *KRT4* | *AURKA* | *MTOR* |
| *BAX* | *MTOR* | *BAX* | *MMP9* | *BCL2* | *LAMC2* | *BAX* | *MUC1* |
| *BCL2* | *MUC1* | *BCL2* | *MTOR* | *BIRC5* | *MAP2K1* | *BCL2* | *MYC* |
| *BDNF* | *MYC* | *BDNF* | *MUC1* | *BMI1* | *MAPK1* | *BDNF* | *NES* |
| *BIRC5* | *NES* | *BIRC5* | *MYC* | *BRAF* | *MAPK3* | *BIRC5* | *NFKB1* |
| *BRAF* | *NFKB1* | *BRAF* | *NES* | *CASP3* | *MDM2* | *BRAF* | *NOD1* |
| *CASP12* | *NOD1* | *CASP12* | *NFKB1* | *CCL14* | *MMP2* | *CASP12* | *NOTCH1* |
| *CASP3* | *NOS2* | *CASP3* | *NOD1* | *CCL2* | *MMP9* | *CASP3* | *NTRK2* |
| *CCL2* | *NOTCH1* | *CCL2* | *NOS2* | *CD274* | *MYC* | *CCL2* | *PDCD1* |
| *CCL3* | *NTRK2* | *CCL3* | *NOTCH1* | *CD47* | *NES* | *CCL3* | *PDPN* |
| *CD274* | *PDCD1* | *CD274* | *NTRK2* | *CDC6* | *NOS2* | *CD274* | *PTGS2* |
| *CDKN1A* | *PDPN* | *CDKN1A* | *PDCD1* | *CDH1* | *NOTCH1* | *CDKN1B* | *RAF1* |
| *CDKN1B* | *PTGS2* | *CDKN1B* | *PDPN* | *CDK4* | *NRAS* | *CDKN2A* | *RB1* |
| *CDKN2A* | *RAF1* | *CDKN2A* | *PTGS2* | *CDK6* | *NTRK2* | *CHEK2* | *RIPK2* |
| *CHEK2* | *RB1* | *CHEK2* | *RAF1* | *CDKN1A* | *PDPN* | *CTNNB1* | *RPS6* |
| *CTNNB1* | *RIPK2* | *CSF1* | *RB1* | *CDKN1B* | *PTGS2* | *CXCL10* | *SMAD4* |
| *CXCL10* | *RPS6* | *CTNNB1* | *RIPK2* | *CDKN1C* | *RAF1* | *CXCL12* | *SNAI1* |
| *CXCL12* | *SMAD4* | *CXCL10* | *RPS6* | *CDKN2A* | *RB1* | *EDN1* | *TEK* |
| *CXCR4* | *SNAI1* | *CXCL12* | *SMAD4* | *CRP* | *RIPK2* | *EGFR* | *TGFA* |
| *EDN1* | *TEK* | *CXCR4* | *SNAI1* | *CSF1* | *RPS6* | *HLA-G* | *TGFB1* |
| *EGFR* | *TGFA* | *EDN1* | *TEK* | *CTNNB1* | *SMAD4* | *HNRNPK* | *TGFB2* |
| *HLA-G* | *TGFB1* | *EGFR* | *TGFA* | *CXCL10* | *SOX2* | *HRAS* | *TGFB3* |
| *HNRNPK* | *TGFB2* | *FOS* | *TGFB1* | *CXCL12* | *SPARC* | *HSPA5* | *TGFBR1* |
| *HRAS* | *TGFB3* | *HLA-G* | *TGFB2* | *CXCL8* | *STAT3* | *HSPB1* | *TNF* |
| *HSPA5* | *TGFBR1* | *HNRNPK* | *TGFB3* | *EDN1* | *TEK* | *HSPD1* | *TNFRSF10A* |
| *HSPB1* | *TNF* | *HRAS* | *TGFBR1* | *EGFR* | *TGFA* | *ICAM1* | *TNFRSF10B* |
| *HSPD1* | *TNFRSF10A* | *HSPA5* | *TNF* | *ERBB2* | *TGFB1* | *IFNG* | *TNFRSF10C* |
| *ICAM1* | *TNFRSF10B* | *HSPB1* | *TNFRSF10A* | *FOXP3* | *TGFB2* | *IL10* | *TNFRSF10D* |
| *IFNG* | *TNFRSF10C* | *HSPD1* | *TNFRSF10B* | *HLA-E* | *TGFB3* | *IL1A* | *TNFSF10* |
| *IL10* | *TNFRSF10D* | *ICAM1* | *TNFRSF10C* | *HLA-G* | *TGFBR1* | *IL1B* | *TP53* |
| *IL1A* | *TNFSF10* | *IFNG* | *TNFRSF10D* | *HRAS* | *TGFBR2* | *IL4* | *TP63* |
| *IL1B* | *TP53* | *IL10* | *TNFSF10* | *IFNG* | *TGFBR3* | *IL6* | *TWIST1* |
| *IL4* | *TP63* | *IL1A* | *TP53* | *IL10* | *TNF* | *ITGA6* | *VEGFA* |
| *IL6* | *TWIST1* | *IL1B* | *TP63* | *IL18* | *TP53* | *JUN* | *WNT5A* |
| *ITGA6* | *VEGFA* | *IL4* | *TWIST1* | *IL1A* | *TP63* | *KRAS* | *YWHAZ* |
| *JUN* | *WNT5A* | *IL6* | *VEGFA* | *IL1B* | *TWIST1* |  |  |
| *KRAS* | *YWHAZ* | *ITGA6* | *WNT5A* | *IL4* | *VEGFA* |  |  |
|  |  | *JUN* | *YWHAZ* | *IL6* | *WNT5A* |  |  |

**b. Molecular Functions**

| **Cytokine receptor  binding** | **Protein binding** | | | | **Protein-containing  complex binding** | | **Signaling receptor  binding** | |
| --- | --- | --- | --- | --- | --- | --- | --- | --- |
| *BDNF* | *ABCB1* | *CXCL8* | *KRAS* | *PDPN* | *ATM* | *KRAS* | *BDNF* | *ITGA2* |
| *CASP3* | *ACKR3* | *CXCR4* | *KRT1* | *PEBP1* | *B2M* | *KRT14* | *CASP3* | *ITGA3* |
| *CCL14* | *AKT1* | *DEFB1* | *KRT10* | *PITX1* | *CASP3* | *LAMA3* | *CCL14* | *ITGA6* |
| *CCL2* | *ALB* | *DEFB103A* | *KRT13* | *PIWIL2* | *CD4* | *MDM2* | *CCL2* | *ITGB6* |
| *CCL3* | *ALDH1A1* | *DEFB4A* | *KRT14* | *POSTN* | *CDH1* | *MLH1* | *CCL3* | *LAMA3* |
| *CSF1* | *ATM* | *DSG2* | *KRT17* | *POU5F1* | *CDKN1A* | *MMP9* | *CD4* | *LAMC2* |
| *CXCL10* | *AURKA* | *DSP* | *KRT18* | *PTGS2* | *CDKN1B* | *MTOR* | *CRP* | *MUC4* |
| *CXCL12* | *B2M* | *EDN1* | *KRT19* | *RAF1* | *CDKN1C* | *MYC* | *CSF1* | *NES* |
| *CXCL8* | *BAX* | *EGFR* | *KRT4* | *RB1* | *CRP* | *NES* | *CXCL10* | *NOTCH1* |
| *DEFB1* | *BCL2* | *ERBB2* | *KRT76* | *RIPK2* | *CXCL12* | *NOD1* | *CXCL12* | *PCNA* |
| *DEFB103A* | *BDNF* | *FOS* | *KRT8* | *RPS6* | *DSP* | *NRAS* | *CXCL8* | *PDPN* |
| *DEFB4A* | *BHLHE40* | *FOXP3* | *KRTAP5-6* | *S100A7* | *EGFR* | *PCNA* | *DEFB1* | *RIPK2* |
| *IFNG* | *BIRC5* | *GPRC5A* | *LAMA3* | *SELE* | *FOS* | *PIWIL2* | *DEFB103A* | *S100A7* |
| *IL10* | *BMI1* | *H2AX* | *LAMC2* | *SLPI* | *HLA-E* | *SMAD4* | *DEFB4A* | *SPP1* |
| *IL17F* | *BRAF* | *HLA-E* | *LDHD* | *SMAD4* | *HSPA5* | *SPARC* | *EDN1* | *STAT3* |
| *IL18* | *BUB1B* | *HLA-G* | *MAP2K1* | *SNAI1* | *HSPD1* | *SPP1* | *ERBB2* | *TGFA* |
| *IL1A* | *CA9* | *HNRNPK* | *MAP2K2* | *SOX2* | *ICAM1* | *TGFB1* | *HLA-E* | *TGFB1* |
| *IL1B* | *CASP3* | *HRAS* | *MAPK1* | *SPARC* | *IL1B* | *TGFB3* | *HLA-G* | *TGFB2* |
| *IL37* | *CCL14* | *HSPA5* | *MAPK3* | *SPP1* | *ITGA2* | *TGFBR1* | *ICAM1* | *TGFB3* |
| *IL4* | *CCL2* | *HSPB1* | *MCM3* | *STAG2* | *ITGA3* | *TGFBR2* | *IFNG* | *TGFBR1* |
| *IL6* | *CCL3* | *HSPBP1* | *MCM7* | *STAT3* | *ITGA6* | *TGFBR3* | *IL10* | *TGFBR2* |
| *NES* | *CCNDBP1* | *HSPD1* | *MDM2* | *STMN1* | *ITGB6* | *TP53* | *IL17F* | *TGFBR3* |
| *TGFB1* | *CD274* | *ICAM1* | *MET* | *TEK* | *JUP* |  | *IL18* | *TNF* |
| *TGFB2* | *CD4* | *IFNG* | *MKI67* | *TGFA* |  |  | *IL1A* | *TNFSF10* |
| *TGFB3* | *CD47* | *IL10* | *MLH1* | *TGFB1* |  |  | *IL1B* | *TP53* |
| *TGFBR1* | *CD68* | *IL17F* | *MMP2* | *TGFB2* |  |  | *IL37* | *VEGFA* |
| *TGFBR2* | *CDC6* | *IL18* | *MMP9* | *TGFB3* |  |  | *IL4* | *WNT5A* |
| *TGFBR3* | *CDH1* | *IL1A* | *MT2A* | *TGFBR1* |  |  | *IL6* |  |
| *TNF* | *CDK2AP1* | *IL1B* | *MTOR* | *TGFBR2* |  |  |  |  |
| *TNFSF10* | *CDK4* | *IL37* | *MTUS1* | *TGFBR3* |  |  |  |  |
| *VEGFA* | *CDK6* | *IL4* | *MUC1* | *TNF* |  |  |  |  |
|  | *CDKN1A* | *IL6* | *MUC4* | *TNFRSF10A* |  |  |  |  |
|  | *CDKN1B* | *IMP3* | *MYC* | *TNFRSF10B* |  |  |  |  |
|  | *CDKN1C* | *ITGA2* | *NES* | *TNFRSF10C* |  |  |  |  |
|  | *CDKN2A* | *ITGA3* | *NFKB1* | *TNFRSF10D* |  |  |  |  |
|  | *CHEK2* | *ITGA6* | *NOD1* | *TNFSF10* |  |  |  |  |
|  | *CP* | *ITGB6* | *NOS2* | *TP53* |  |  |  |  |
|  | *CRP* | *IVL* | *NOTCH1* | *TP63* |  |  |  |  |
|  | *CSF1* | *JUN* | *NRAS* | *TWIST1* |  |  |  |  |
|  | *CTNNB1* | *JUP* | *NTRK2* | *VEGFA* |  |  |  |  |
|  | *CXCL10* | *KCNC4* | *PCNA* | *WNT5A* |  |  |  |  |
|  | *CXCL12* | *KCNH2* | *PDCD1* | *YWHAZ* |  |  |  |  |

**c. Cellular Components**

| **Extracellular space** | | **Extracellular region** | | | **Cell surface** | **Vesicle** | |
| --- | --- | --- | --- | --- | --- | --- | --- |
| *ABCB1* | *IL37* | *ABCB1* | *HLA-E* | *MET* | *ABCB1* | *ABCB1* | *KRT14* |
| *ALB* | *IL4* | *ALB* | *HLA-G* | *MMP1* | *ACKR3* | *ACKR3* | *KRT18* |
| *ALDH1A1* | *IL6* | *ALDH1A1* | *HNRNPK* | *MMP2* | *B2M* | *AKT1* | *KRT19* |
| *ALDH2* | *ITGA3* | *ALDH2* | *HSPA5* | *MMP9* | *CD274* | *ALB* | *KRT76* |
| *B2M* | *IVL* | *B2M* | *HSPB1* | *MTUS1* | *CD4* | *ALDH1A1* | *KRT8* |
| *BAX* | *JUP* | *BAX* | *HSPD1* | *MUC1* | *CD47* | *ALDH2* | *LAMA3* |
| *BDNF* | *KRT1* | *BDNF* | *ICAM1* | *MUC4* | *CXCL10* | *ATM* | *LDHD* |
| *CCL14* | *KRT10* | *CCL14* | *IFNG* | *NFKB1* | *CXCL12* | *B2M* | *MAP2K1* |
| *CCL2* | *KRT13* | *CCL2* | *IL10* | *NOTCH1* | *CXCR4* | *BAX* | *MAP2K2* |
| *CCL3* | *KRT14* | *CCL3* | *IL17F* | *NRAS* | *DSG2* | *BDNF* | *MAPK1* |
| *CD274* | *KRT18* | *CD274* | *IL18* | *PCNA* | *EGFR* | *CD274* | *MAPK3* |
| *CD47* | *KRT19* | *CD47* | *IL1A* | *PEBP1* | *HLA-E* | *CD4* | *MDM2* |
| *CDH1* | *KRT76* | *CDC6* | *IL1B* | *POSTN* | *HLA-G* | *CD47* | *MMP9* |
| *CP* | *KRT8* | *CDH1* | *IL37* | *S100A7* | *HSPA5* | *CD68* | *MTOR* |
| *CRP* | *LAMA3* | *CP* | *IL4* | *SELE* | *HSPD1* | *CDH1* | *MUC1* |
| *CSF1* | *LAMC2* | *CRP* | *IL6* | *SLPI* | *ICAM1* | *CDKN1B* | *MUC4* |
| *CTNNB1* | *MMP2* | *CSF1* | *ITGA3* | *SPARC* | *IL1A* | *CP* | *NFKB1* |
| *CXCL10* | *MMP9* | *CTNNB1* | *IVL* | *SPP1* | *ITGA2* | *CRP* | *NOD1* |
| *CXCL12* | *MTUS1* | *CXCL10* | *JUP* | *STMN1* | *ITGA3* | *CTNNB1* | *NOS2* |
| *CXCL8* | *MUC1* | *CXCL12* | *KRT1* | *TEK* | *ITGA6* | *CXCL12* | *NOTCH1* |
| *CXCR4* | *MUC4* | *CXCL8* | *KRT10* | *TGFA* | *ITGB6* | *CXCR4* | *NRAS* |
| *DEFB1* | *NRAS* | *CXCR4* | *KRT13* | *TGFB1* | *KCNH2* | *DEFB1* | *NTRK2* |
| *DEFB103A* | *PCNA* | *DEFB1* | *KRT14* | *TGFB2* | *KRT10* | *DSG2* | *PCNA* |
| *DEFB4A* | *PEBP1* | *DEFB103A* | *KRT18* | *TGFB3* | *KRT4* | *DSP* | *PDPN* |
| *DSG2* | *POSTN* | *DEFB4A* | *KRT19* | *TGFBR3* | *MET* | *EDN1* | *PEBP1* |
| *DSP* | *S100A7* | *DSG2* | *KRT76* | *TGM3* | *NOTCH1* | *EGFR* | *RIPK2* |
| *EDN1* | *SELE* | *DSP* | *KRT8* | *TNF* | *PDCD1* | *ERBB2* | *S100A7* |
| *EGFR* | *SLPI* | *EDN1* | *LAMA3* | *TNFSF10* | *SELE* | *GPRC5A* | *SLPI* |
| *GPRC5A* | *SPARC* | *EGFR* | *LAMC2* | *VEGFA* | *SPARC* | *H2AX* | *SPARC* |
| *H2AX* | *SPP1* | *GPRC5A* | *MAP2K2* | *WNT5A* | *TEK* | *HLA-E* | *SPP1* |
| *HLA-E* | *STMN1* | *H2AX* | *MAPK1* | *YWHAZ* | *TGFA* | *HLA-G* | *STMN1* |
| *HLA-G* | *TGFA* |  |  |  | *TGFB1* | *HNRNPK* | *TGFA* |
| *HNRNPK* | *TGFB1* |  |  |  | *TGFB3* | *HSPA5* | *TGFB1* |
| *HSPA5* | *TGFB2* |  |  |  | *TGFBR1* | *HSPB1* | *TGFB2* |
| *HSPB1* | *TGFB3* |  |  |  | *TGFBR2* | *HSPD1* | *TGFB3* |
| *HSPD1* | *TGFBR3* |  |  |  | *TGFBR3* | *ICAM1* | *TGFBR1* |
| *ICAM1* | *TGM3* |  |  |  | *TNF* | *IL1B* | *TGFBR3* |
| *IFNG* | *TNF* |  |  |  | *TNFRSF10A* | *ITGA3* | *TGM3* |
| *IL10* | *TNFSF10* |  |  |  | *TNFRSF10B* | *IVL* | *TNF* |
| *IL17F* | *VEGFA* |  |  |  | *TNFRSF10C* | *JUP* | *TNFSF10* |
| *IL18* | *WNT5A* |  |  |  | *TNFRSF10D* | *KRT1* | *VEGFA* |
| *IL1A* | *YWHAZ* |  |  |  | *VEGFA* | *KRT10* | *WNT5A* |
| *IL1B* |  |  |  |  | *WNT5A* | *KRT13* | *YWHAZ* |

# Appendix 10. Characteristics of assessed proteins among included studies presenting clinical correlation in the TCGA comparing OSCC and normal samples (n=77).

| Gene | Protein | TCGA Feature | p-value^1^ | Number of studies |
| --- | --- | --- | --- | --- |
| *ACKR3* | Atypical chemokine receptor 3 | Margin Status | 0.042 | 1 |
|  |  | Pathologic N status | 0.047 |  |
| *ALDH1A1* | Retinal dehydrogenase 1 | Pathologic T | 0.019 | 1 |
|  |  | Pathologic T grouped | 0.006 |  |
| *ALDH2* | Aldehyde dehydrogenase, mitochondrial | Final vital status | 0.021 | 1 |
|  |  | Pathologic N status 1 | 0.039 |  |
|  |  | Pathologic T | 0.027 |  |
| *B2M** | Beta-2-microglobulin | Neoplasm histologic grade | 0.023 | 1 |
|  |  | Pathologic N status | 0.017 |  |
|  |  | Pathologic stage | 0.040 |  |
| *BAX* | Apoptosis regulator BAX | Pathologic T | 0.041 | 2 |
| *BCL2* | Apoptosis regulator Bcl-2 | Pathologic T | 0.035 | 6 |
|  |  | Pathologic T grouped | 0.035 |  |
| *BHLHE40* | Class E basic helix-loop-helix protein 40 (DEC1) | Neoplasm histologic grade | 0.017 | 1 |
| *BMI1* | Polycomb complex protein BMI-1 | Lymphovascular invasion present | 0.029 | 2 |
| *BRAF* | Serine/threonine-protein kinase B-raf | New tumor event after initial treatment follow-up | 0.012 | 1 |
|  |  | Primary therapy outcome success | 0.022 |  |
| *CASP12* | Inactive caspase-12 | HPV status by ISH testing | 0.031 | 1 |
|  |  | Lymphovascular invasion present | 0.029 |  |
| *CCL2* | C-C motif chemokine 2 | Neoplasm histologic grade | 0.018 | 3 |
|  |  | Pathologic N status 1 | 0.045 |  |
|  |  | Pathologic T grouped | 0.014 |  |
| *CCL3* | C-C motif chemokine 3 | Final Vital Status | 0.001 | 1 |
| *CCNDBP1* | Cyclin-D1-binding protein 1 | Neoplasm histologic grade | 0.016 | 3 |
|  |  | Pathologic N status | 0.035 |  |
| *CD274* | Programmed cell death 1 ligand 1 (PD-L1) | Neoplasm histologic grade | 0.008 | 4 |
| *CD47* | Leukocyte surface antigen CD47 | Final Vital Status | 0.010 | 1 |
|  |  | Neoplasm histologic grade | 0.016 |  |
|  |  | Pathologic stage | 0.004 |  |
|  |  | Pathologic stage grouped | 0.002 |  |
|  |  | Perineural invasion present | 0.004 |  |
| *CD68* | Macrosialin | New tumor event after initial treatment follow-up | 0.029 | 1 |
| *CDH1* | Cadherin-1 | Pathologic T grouped | 0.041 | 3 |
| *CDK6* | Cyclin-dependent kinase 6 | Lymphovascular invasion present | 0.043 | 1 |
|  |  | Primary therapy outcome success | 0.025 |  |
| *CDKN1C* | Cyclin-dependent kinase inhibitor 1C (p57kip2) | Perineural invasion present | 0.018 | 1 |
| *CDKN2A** | Cyclin-dependent kinase inhibitor 2A (p16^INK4a^) | Alcohol history documented | 0.009 | 5 |
|  |  | Final Vital Status | 0.047 |  |
| *CXCL12* | Stromal cell-derived factor 1 | Pathologic T grouped | 0.019 | 1 |
|  |  | Perineural invasion present | 0.045 |  |
| *CXCL8* | Interleukin-8 | Alcohol history documented | 0.004 | 2 |
| *DSP* | Desmoplakin | Lymphovascular invasion present | 0.007 | 1 |
| *EDN1* | Endothelin-1 | Margin status | 0.021 | 2 |
| *EGFR* | Epidermal growth factor receptor | Alcohol history documented | 0.010 | 3 |
| *ERBB2* | Receptor tyrosine-protein kinase erbB-2 | Lymphovascular invasion present | 0.045 | 2 |
|  |  | Pathologic T grouped | 0.043 |  |
| *FOS* | Proto-oncogene c-Fos | Margin status | 0.025 | 1 |
| *FOXP3* | Forkhead box protein P3 | Pathologic T grouped | 0.025 | 1 |
| *HLA-G* | HLA class I histocompatibility antigen, alpha chain G | Presence of pathological nodal extracapsular spread | 0.007 | 1 |
| *HSPB1* | Heat shock protein beta-1 | Neoplasm histologic grade | 0.005 | 1 |
| *HSPD1* | 60 kDa heat shock protein, mitochondrial | HPV status by ISH testing | 0.000 | 1 |
|  |  | Pathologic T | 0.017 |  |
| *hTERT* | Telomerase reverse transcriptase | HPV status by ISH testing | 0.046 | 2 |
| *IFNG* | Interferon gamma | Neoplasm histologic grade | 0.028 | 1 |
|  |  | Pathologic T grouped | 0.037 |  |
| *IL37* | Interleukin-37 | Pathologic stage | 0.029 | 2 |
|  |  | Pathologic stage grouped | 0.019 |  |
|  |  | Pathologic T grouped | 0.032 |  |
| *IL4* | Interleukin-4 | Final Vital Status | 0.023 | 1 |
|  |  | Neoplasm histologic grade | 0.019 |  |
| *ITGA3* | Integrin alpha-3 (α3𝛽l integrin) | Pathologic N status 1 | 0.038 | 1 |
| *ITGA6* | Integrin alpha-6 (α6𝛽4 integrin) | Tobacco smoking history | 0.040 | 1 |
| *ITGB6* | Integrin beta-6 (avb6 integrin) | Neoplasm histologic grade | 0.009 | 1 |
| *IVL* | Involucrin | Pathologic N status | 0.021 | 1 |
| *JUN* | Transcription factor AP-1 | Pathologic T | 0.016 | 2 |
|  |  | Pathologic T grouped | 0.031 |  |
| *KCNC4* | Potassium voltage-gated channel subfamily C member 4 | Margin status | 0.027 | 1 |
|  |  | Presence of pathological nodal extracapsular spread | 0.029 |  |
| *KRT1* | Keratin, type II cytoskeletal 1 | Neoplasm histologic grade | 0.037 | 2 |
| *KRT10* | Keratin, type I cytoskeletal 10 | Neoplasm histologic grade | 0.011 | 1 |
| *KRT13* | Keratin, type I cytoskeletal 13 | Pathologic N status | 0.007 | 3 |
|  |  | Pathologic stage grouped | 0.020 |  |
| *KRT18* | Keratin, type I cytoskeletal 18 | Final Vital Status | 0.050 | 2 |
| *KRT19* | Keratin, type I cytoskeletal 19 | HPV status by ISH testing | 0.039 | 3 |
|  |  | Pathologic T grouped | 0.039 |  |
| *KRT4* | Keratin, type II cytoskeletal 4 | Pathologic N status | 0.005 | 1 |
| *KRT76* | Keratin, type II cytoskeletal 2 oral | Pathologic N status | 0.009 | 1 |
| *KRT8* | Keratin, type II cytoskeletal 8 | Presence of pathological nodal extracapsular | 0.049 | 3 |
| *LAMA3* | Laminin subunit alpha-3 (Laminin-5) | Final Vital Status | 0.028 | 1 |
|  |  | Neoplasm histologic grade | 0.021 |  |
| *LAMC2* | Laminin subunit gamma-2 | Neoplasm histologic grade | 0.039 | 1 |
|  |  | Pathologic N status | 0.034 |  |
|  |  | Pathologic stage | 0.020 |  |
|  |  | Pathologic stage grouped | 0.005 |  |
| *MAP2K1* | Dual specificity mitogen-activated protein kinase kinase 1 | Final vital status | 0.027 | 1 |
| *MAP2K2* | Dual specificity mitogen-activated protein kinase kinase 2 | Lymphovascular invasion present | 0.01 | 1 |
|  |  | Primary therapy outcome success | 0.026 |  |
| *MAPK3* | Mitogen-activated protein kinase 3 | Margin status | 0.007 | 2 |
| *MDM2* | E3 ubiquitin-protein ligase Mdm2 | Pathologic T | 0.047 | 2 |
|  |  | Pathologic T grouped | 0.015 |  |
| *MET* | Hepatocyte growth factor receptor | Alcohol history documented | 0.008 | 1 |
|  |  | Pathologic stage grouped | 0.038 |  |
| *MLH1* | DNA mismatch repair protein Mlh1 | Neoplasm histologic grade | 0.004 | 1 |
| *MMP9* | Matrix metalloproteinase-9 | HPV status by p16 testing | 0.033 | 1 |
|  |  | New tumor event after initial treatment follow-up | 0.045 |  |
| *MTUS1* | Microtubule-associated tumor suppressor 1 | HPV status by p16 testing | 0.014 | 1 |
| *PDCD1* | Programmed cell death protein 1 (PD-1) | Neoplasm histologic grade | 0.005 | 2 |
|  |  | Pathologic T grouped | 0.009 |  |
| *PDPN* | Podoplanin | Pathologic N status | 0.017 | 4 |
|  |  | Pathologic stage grouped | 0.037 |  |
| *PEBP1* | Phosphatidylethanolamine-binding protein 1 (RKIP) | Tobacco smoking history | 0.021 | 1 |
| *POSTN* | Periostin | Margin status | 0.045 | 2 |
|  |  | Perineural invasion present | 0.036 |  |
| *RB1** | Retinoblastoma-associated protein (pRb) | Tobacco smoking history | 0.042 | 4 |
| *RIPK2* | Receptor-interacting serine/threonine-protein kinase 2 | Lymphovascular invasion present | 0.025 | 1 |
| *SMAD4** | Mothers against decapentaplegic homolog 4 | HPV status by p16 testing | 0.030 | 1 |
| *SPARC* | SPARC (Osteonectin) | Margin status | 0.018 | 1 |
| *STAG2* | Cohesin subunit SA-2 | HPV status by ISH testing | 0.027 | 1 |
| *TGFA* | Protransforming growth factor alpha | Perineural invasion present | 0.045 | 1 |
| *TGFB1* | Transforming growth factor beta-1 proprotein | Pathologic N status | 0.005 | 4 |
| *TGFBR1* | TGF-beta receptor type-1 | Alcohol history documented | 0.040 | 1 |
|  |  | HPV status by p16 testing | 0.023 |  |
| *TGFBR3* | TGF-beta receptor type-3 | Pathologic T | 0.009 | 1 |
|  |  | Pathologic T grouped | 0.003 |  |
| *TGM3* | Protein-glutamine gamma-glutamyltransferase E | Pathologic N status | 0.004 | 1 |
|  |  | Pathologic stage grouped | 0.008 |  |
| *TNF* | Tumor necrosis factor | Final Vital Status | 0.048 | 5 |
|  |  | Neoplasm histologic grade | 0.008 |  |
| *TNFSF10* | Tumor necrosis factor ligand superfamily member 10 | Neoplasm histologic grade | 0.019 | 1 |
|  |  | Pathologic stage | 0.046 |  |
| *TP53** | Cellular tumor antigen p53 | Neoplasm histologic grade | 0.019 | 15 |
| *TWIST1* | Twist-related protein 1 | Perineural invasion present | 0.043 | 1 |

^1^ The normality of gene expression data was assessed with Shapiro-Wilk test (α=0.05). Student’s t-test or Welch’s test (two group comparisons), and ANOVA with post-hoc Tukey test (>2 groups) were used for parametric data. Non-parametric data were analysed with Wilcoxon (two groups) or Kruskal-Wallis (>2 groups) tests. Significance threshold p-value ≤ 0.05.

* HNSCC Driver Gene

# Appendix 11. Grading of Recommendation, Assessment, Development, and Evaluation (GRADE) evidence profile.

Proteins expression assessed by odds ratio among OL and OSCC.

| **Certainty assessment** | | | | | | | **№ of patients** | | **Effect** | | **Certainty** | **Importance** |
| --- | --- | --- | --- | --- | --- | --- | --- | --- | --- | --- | --- | --- |
| **№ of studies** | **Study design** | **Risk of bias** | **Inconsistency** | **Indirectness** | **Imprecision** | **Other considerations** | **OL** | **OSCC** | **Relative (95% CI)** | **Absolute (95% CI)** |  |  |
| **TP53** | | | | | | | | | | | | |
| 5 | observational studies | serious ^a^ | serious ^b^ | not serious | not serious | strong association | 92/181 (50.8%) | 179/318 (56.3%) | **OR 1.00** (0.26 to 3.82) | **0 fewer per 1.000** (from 312 fewer to 268 more) | ⨁◯◯◯ VERY LOW | NOT IMPORTANT |
| **BIRC5** | | | | | | | | | | | | |
| 5 | observational studies | serious ^a^ | not serious | not serious | not serious | strong association | 89/117 (76.1%) | 92/115 (80.0%) | **OR 0.57** (0.29 to 1.13) | **105 fewer per 1.000** (from 263 fewer to 19 more) | ⨁⨁◯◯ LOW | IMPORTANT |
| **RB1** | | | | | | | | | | | | |
| 3 | observational studies | serious ^a^ | not serious | not serious | not serious | very strong association | 150/202 (74.3%) | 213/358 (59.5%) | **OR 1.72** (1.14 to 2.60) | **121 more per 1.000** (from 31 more to 198 more) | ⨁⨁⨁◯ MODERATE | CRITICAL |
| **p21** | | | | | | | | | | | | |
| 3 | observational studies | serious ^a^ | not serious | not serious | not serious | strong association | 65/100 (65.0%) | 79/111 (71.2%) | **OR 0.81** (0.43 to 1.50) | **45 fewer per 1.000** (from 191 fewer to 76 more) | ⨁⨁◯◯ LOW | IMPORTANT |
| **Keratins** | | | | | | | | | | | | |
| 4 | observational studies | serious ^a^ | very serious ^b^ | not serious | serious ^c^ | strong association | 391/603 (64.8%) | 379/717 (52.9%) | **OR 1.84** (0.33 to 10.32) | **145 more per 1.000** (from 259 fewer to 392 more) | ⨁◯◯◯ VERY LOW | NOT IMPORTANT |
| **CCNDBP1** | | | | | | | | | | | | |
| 2 | observational studies | serious ^a^ | very serious ^b^ | not serious | serious ^c^ | strong association | 43/131 (32.8%) | 150/244 (61.5%) | **OR 0.13** (0.01 to 2.61) | **443 fewer per 1.000** (from 599 fewer to 192 more) | ⨁◯◯◯ VERY LOW | NOT IMPORTANT |
| **CDH1** | | | | | | | | | | | | |
| 2 | observational studies | not serious | not serious | not serious | serious ^c^ | very strong association | 61/61 (100.0%) | 21/32 (65.6%) | **OR 33.66** (4.10 to 275.62) | **328 more per 1.000** (from 229 more to 342 more) | ⨁⨁⨁◯ MODERATE | CRITICAL |
| **EGFR** | | | | | | | | | | | | |
| 2 | observational studies | serious ^a^ | not serious | not serious | not serious | strong association | 89/100 (89.0%) | 66/70 (94.3%) | **OR 0.59** (0.18 to 1.98) | **36 fewer per 1.000** (from 195 fewer to 27 more) | ⨁⨁◯◯ LOW | IMPORTANT |
| **MDM2** | | | | | | | | | | | | |
| 2 | observational studies | serious ^d^ | not serious | not serious | not serious | very strong association | 48/123 (39.0%) | 101/168 (60.1%) | **OR 0.44** (0.24 to 0.81) | **192 fewer per 1.000** (from 304 fewer to 71 fewer) | ⨁⨁⨁◯ MODERATE | CRITICAL |
| **p16** | | | | | | | | | | | | |
| 3 | observational studies | not serious | not serious | not serious | not serious | strong association | 50/142 (35.2%) | 105/271 (38.7%) | **OR 1.02** (0.66 to 1.58) | **5 more per 1.000** (from 93 fewer to 112 more) | ⨁⨁⨁◯ MODERATE | IMPORTANT |
| **CD274** | | | | | | | | | | | | |
| 2 | observational studies | not serious | not serious | not serious | serious ^c^ | very strong association | 34/100 (34.0%) | 112/147 (76.2%) | **OR 0.12** (0.04 to 0.40) | **484 fewer per 1.000** (from 648 fewer to 201 fewer) | ⨁⨁⨁◯ MODERATE | CRITICAL |
| **PDPN** | | | | | | | | | | | | |
| 2 | observational studies | serious ^a^ | not serious | not serious | not serious | very strong association | 38/57 (66.7%) | 71/80 (88.8%) | **OR 0.21** (0.04 to 1.04) | **243 fewer per 1.000** (from 472 fewer to 72 fewer) | ⨁⨁⨁◯ MODERATE | CRITICAL |
| **BCL2** | | | | | | | | | | | | |
| 4 | observational studies | serious ^a^ | not serious | not serious | not serious | strong association | 75/97 (77.3%) | 0.0% | **OR 1.22** (0.45 to 3.32) | **0 fewer per 1.000** (from 0 fewer to 0 fewer) | ⨁⨁◯◯ LOW | IMPORTANT |
| **MUC4** | | | | | | | | | | | | |
| 2 | observational studies | not serious | not serious | not serious | not serious | strong association | 18/39 (46.2%) | 36/44 (81.8%) | **OR 0.18** (0.04 to 0.86) | **371 fewer per 1.000** (from 666 fewer to 24 fewer) | ⨁⨁⨁◯ MODERATE | CRITICAL |
| **POSTN** | | | | | | | | | | | | |
| 2 | observational studies | serious ^a^ | not serious | not serious | not serious | very strong association | 42/61 (68.9%) | 63/66 (95.5%) | **OR 0.10** (0.03 to 0.37) | **277 fewer per 1.000** (from 568 fewer to 69 fewer) | ⨁⨁⨁◯ MODERATE | CRITICAL |

**CI:** Confidence interval; **OR:** Odds ratio

#### Explanations

a. Moderate to high risk of bias

b. High heterogeneity (I2)

c. Large confidence interval

d. High risk of bias
